# Insights into Heterocycle Biosynthesis in the Cytotoxic Polyketide Alkaloid Janustatin A from a Plant-Associated Bacterium

**DOI:** 10.1021/acs.biochem.4c00542

**Published:** 2025-01-09

**Authors:** Stefan Leopold-Messer, Pornsuda Chawengrum, Jörn Piel

**Affiliations:** † Institute of Microbiology, Eidgenössische Technische Hochschule (ETH) Zurich, Vladimir-Prelog-Weg 4, 8093 Zurich, Switzerland; ‡ Chemical Biology Program, Chulabhorn Graduate Institute, Chulabhorn Royal Academy, Kamphaeng Phet 6 Road, Laksi, Bangkok 10210, Thailand

## Abstract

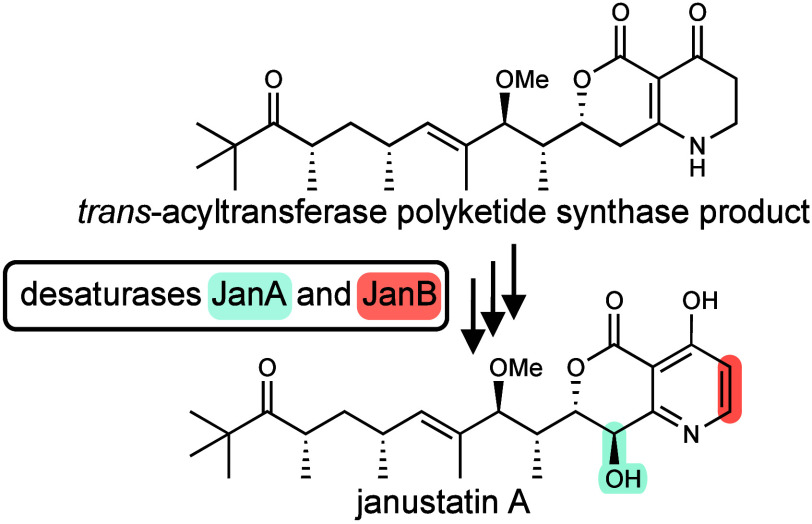

Janustatin A is a potently cytotoxic polyketide alkaloid produced at trace amounts by the marine bacterial plant symbiont *Gynuella sunshinyii*. Its biosynthetic terminus features an unusual pyridine-containing bicyclic system of unclear origin, in which polyketide and amino acid extension units appear reversed compared to the order of enzymatic modules in the polyketide synthase (PKS)-nonribosomal peptide synthetase (NRPS) assembly line. To elucidate unknown steps in heterocycle formation, we first established robust genome engineering tools in *G*. *sunshinyii*. A combination of gene deletion, complementation, production improvement, and NMR experiments then demonstrated that two desaturase homologues, JanA and JanB, are involved in hydroxylation and pyridine formation by desaturation, respectively. Structure–activity relationship studies showed that these modifications substantially increase the cytotoxicity and that the fully functionalized heterocyclic system is crucial for sub-nanomolar cytotoxicity. Isolation of the early post-PKS intermediate janustatin D with an already reversed heterocycle topology supports a noncanonical rearrangement process occurring on the PKS-NRPS assembly line.

Bacterial *trans*-acyltransferase polyketide synthases (*trans*-AT PKSs) are a major family of natural product biosynthetic enzymes with largely untapped potential for drug discovery due to their prevalence in bacteria from lesser-studied taxa and environments. An example is the marine bacterium *Gynuella sunshinyii* (order Oceanospirillales), a plant symbiont associated with monocotyledonous halophytes^1,2^ that contains six *trans*-AT PKS biosynthetic gene clusters (BGCs) for diverse bioactive polyketides.^3^ Of these, janustatin A (**1**, Figure 1) isolated from *G. sunshinyii* YC6258^T^ stands out as an exceptionally potent cytotoxin that displays an unusual delayed cytotoxic effect on cancer cells at subnanomolar concentrations.^3^

**Figure 1:**
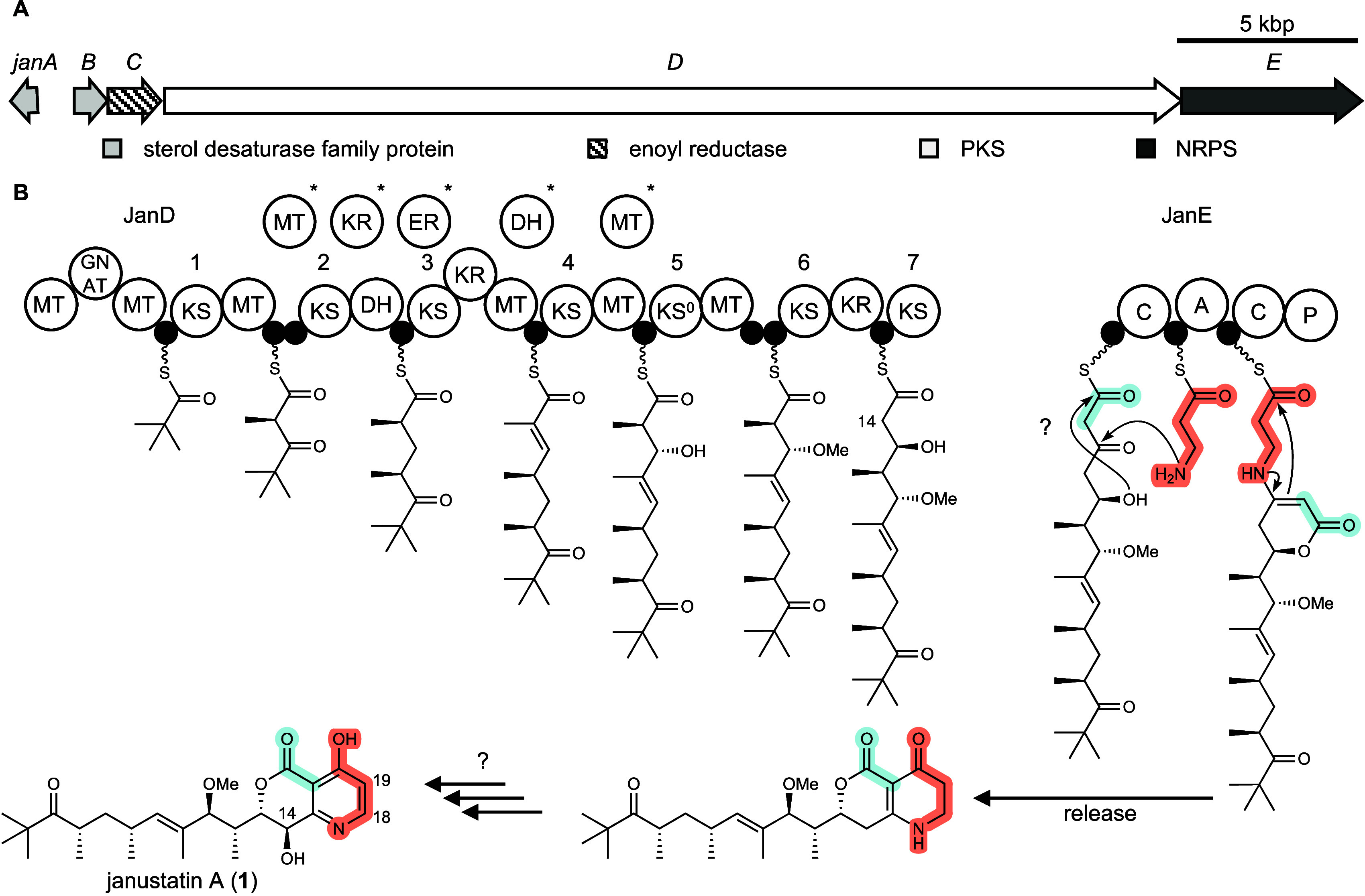
Janustatin A (**1**) and proposed biosynthesis. **A**, Organization of the *jan* BGC. In addition to the PKS (*janCD*) and NRPS (*janE*) genes, the BGC contains *janA* and *janB* of unknown function that encode homologues of desaturases. **B**, Architecture of the *jan* PKS-NRPS system and hypothetical biosynthetic intermediates based on *trans*-AT PKS collinearity rules.^3,9,10^ The NRPS module JanE was shown to install β-alanine (position highlighted in **1**). Based on the characterized structure of **1**, a reversed building block order, in which the amino group attacks the β-keto position instead of the thioester, might occur,^3^ but a rearrangement after release from the PKS-NRPS assembly line could not be excluded. KS, ketosynthase domain; KS^0^, nonelongating KS; GNAT, GCN5-related *N*-acetyltransferase family; KR, ketoreductase; DH, dehydratase; ER, enoyl reductase JanC; MT, methyltransferase; C, condensation domain; A adenylation domain; P, NRPS-para261 domain; small black circles in the assembly line denote acyl or peptidyl carrier protein domains; ?, unresolved biosynthetic steps;*, hypothetical *trans*-acting domains, perhaps from other modules in JanD, speculated to complement missing domains in some PKS modules. Adapted with permission from ref (3). Copyright 2022 Springer Nature.

**1** is a polyketide alkaloid that features an unusual pyridine-containing bicyclic moiety linked to an exocyclic, polymethylated chain. Knockout studies linked one of the six *trans*-AT PKS BGCs in the producer genome, termed the *jan* cluster, to janustatin biosynthesis.^3^ This BGC (Figure 1) encodes a multimodular enzymatic assembly line featuring eight *trans*-AT PKS modules and one nonribosomal peptide synthetase (NRPS) module. Feeding experiments established that the NRPS module incorporates the nitrogen and three carbon atoms of β-alanine into the pyridine ring.^3^ This result was unexpected based on PKS-NRPS biosynthetic logic, since it suggested that the order of the last two biosynthetic modules (PKS followed by an NRPS module) is reversed as compared to the order of building blocks in the janustatin chain (β-alanine followed by a C2 unit at the ester terminus, Figure 1). Considering that the *jan* BGC harbors further, non-PKS-NRPS genes with unknown function, this observation raised questions about the identity of the compound released from the assembly line and how it is converted to the fully functionalized heterocyclic system. In addition, the importance of the unusual alkaloid moiety for bioactivity remained unknown.

Here we characterized the final steps of janustatin biosynthesis by assigning roles of the desaturase homologues JanA and JanB as post-PKS heterocycle processing enzymes. The data provide insights into structure–activity relationships for the alkaloid moiety and suggest that the noncanonical reversal of biosynthetic units occurs on the PKS-NRPS assembly line.

In previous work, bioinformatic analyses and feeding studies had assigned all PKS and NRPS units to building blocks in janustatin A (**1**) but left the biosynthetic steps for heterocycle formation and the biochemical functions of the JanE terminus and accessory enzymes JanA (NCBI accession number AJQ97476) and JanB (AJQ97474) unclear. The C-terminus of the NRPS protein JanE features a NRPS-para261 domain (TIGR01720), in the following referred to as P domain.^3−5^ The P domain is a 171 amino acid long protein family model with conserved RxxPxxGxxYGxL and FNYLGxxD motifs, which are also present in JanE (Figure S1).^4^ While P domains are present in several NRPS systems, no reports on their function exist to our knowledge. As other unassigned features, the *jan* BGC encodes the desaturase-like proteins JanA and JanB, that share about 29% mutual sequence identity (Figure S2). JanA and JanB structures predicted by AlphaFold^6,7^ suggest a very similar fold to that of sphingolipid α-hydroxylase (Scs7p, PDB: 4ZR0, Figure S3). The two metal-binding HxxHH motifs^8^ that are typical for desaturases and hydroxylases are present in JanA and JanB and align well with those of characterized members of these enzyme families (Figure S2, S3).

To study the janustatin pathway, a robust method for gene deletions in *G*. *sunshinyii* had to be developed. For this purpose, 500 bp fragments homologous to genome regions up- and downstream of genes of interest were cloned into the suicide vector pSW8197 (Supporting Information, Table S1).^11^ Counterselection in this system is based on the toxin gene *ccdB* controlled by the pBAD promoter. Arabinose can therefore be used to induce CcdB production. Unfortunately, conjugative transfer of suicide plasmids into *G*. *sunshinyii* and tests for plasmid integration by PCR revealed deletions and insertions in *ccdB* (Figure S4). Therefore, we changed to a different selection system based on the plasmid pEB17,^12^ which utilizes *sacB*-mediated counterselection. After assembly in *Escherichia coli* DH5α the suicide plasmids were sequenced and introduced into the auxotrophic *E*. *coli* donor strain ST18.^13^ Plasmids were subsequently conjugated into *G*. *sunshinyii*, and single crossover recombinants, with the plasmid integrated into the bacterial genome, were identified by antibiotic selection and PCR. After one day of further growth under nonselective conditions, double crossover mutants were obtained by sucrose counterselection. PCRs were used to differentiate wild-type revertants from desired mutants and to verify correct genetic modifications (see Supporting Information for details, Figure S5, Table S2). This conjugative genome engineering method proved to be more reproducible than the previously reported electroporation protocol,^3^ which suffered from a high amount of false positive clones. Previously, we have successfully used this updated protocol to engineer a *trans*-AT PKS.^14^

With this method at hand, we set out to study the unknown steps of janustatin biosynthesis. Based on the module architecture and feeding experiments, janustatin biosynthesis on the PKS/NRPS would terminate with the introduction of β-alanine by the NRPS JanE (Figure 1).^3^ To form the peptide bond with the polyketide chain, only one condensation (C) domain, catalyzing peptide bond formation, and one adenylation (A) domain, supplying the amino acid, would be necessary. JanE, however, contains an additional C domain, followed by the P domain of unknown function.^4^ We suspected these two domains might be involved in either the biosynthetic unit switch or chain release. To preliminarily study the role of the P domain, it was deleted and organic extracts of the resulting *G*. *sunshinyii* mutant were analyzed by liquid chromatography - mass spectrometry (LC-MS). The metabolic profiles showed that janustatin A (**1**) production was almost completely abolished (Figure 2, Figure S6, Table S3). This suggests that the P domain makes an essential contribution to janustatin biosynthesis, but its exact biosynthetic role remains elusive.

**Figure 2:**
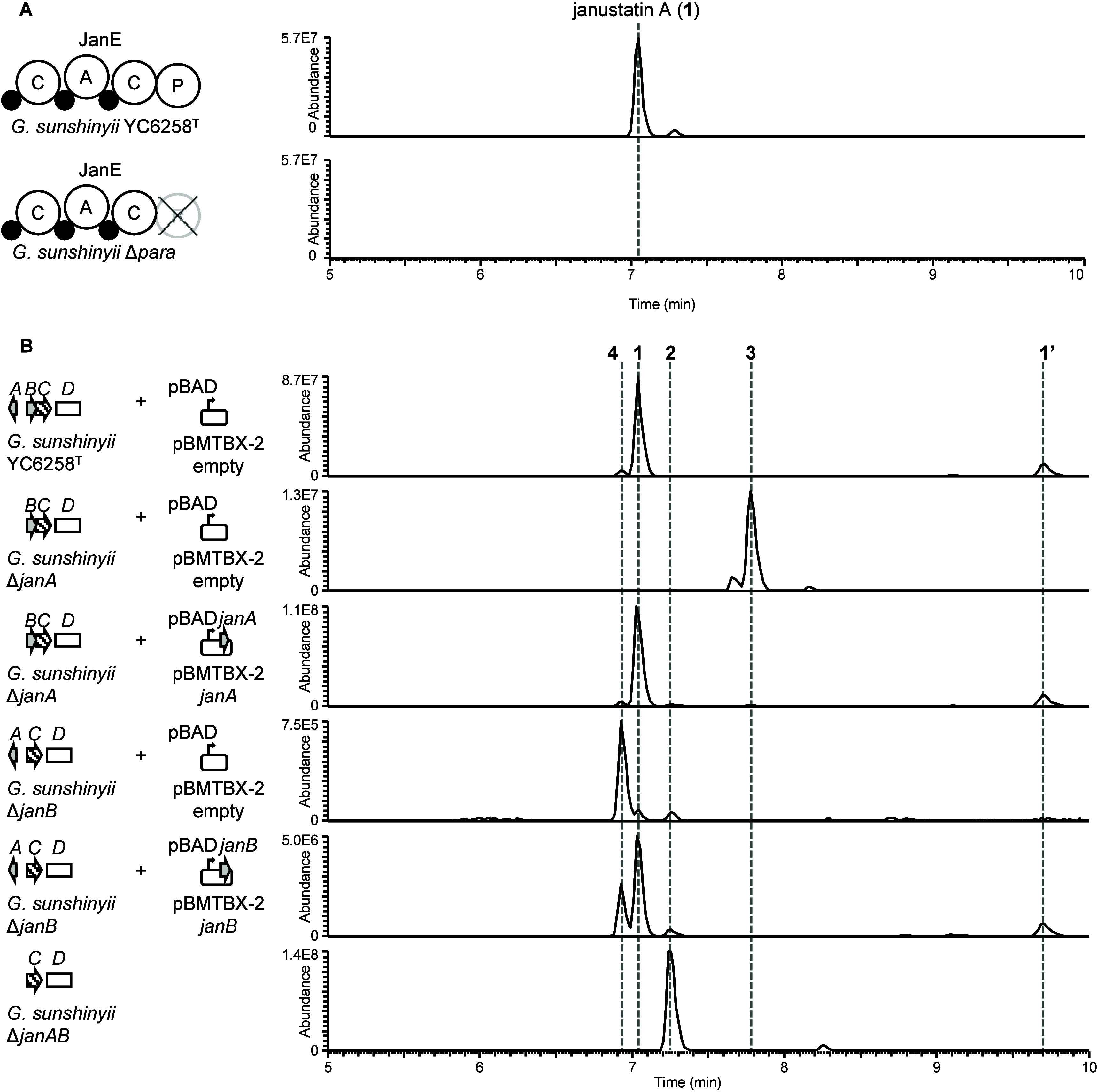
LC-MS profiles of *G. sunshinyii* deletion and complementation mutant extracts. **A**, Left: Domain organization of the NRPS protein JanE in the wild-type *G. sunshinyii* and a P domain deletion mutant. Right: Corresponding LC-MS profiles of organic extracts (scale fixed to 5.66E7). **B**, Left: Local *jan* BGC organization (compare Figure 1) of *G*. *sunshinyii* mutants complemented with the deleted genes on plasmids. Right: Corresponding LC-MS profiles of organic extracts. Plots show extracted ion chromatograms (*m*/*z* 432.2744, 434.2901, 448.2694, 450.2850). Experiments were performed in biological triplicates (Figure S6, and S8, Table S3, and S8). See Figure S10 for details on janustatin B (**1**′).

To investigate the function of the desaturase-like proteins JanA and JanB, we created three gene deletion mutants: *G*. *sunshinyii* Δ*janA*, Δ*janB*, and Δ*janAB* (Figure S6). After cultivation and extraction of these strains, organic extracts of the supernatant were analyzed by LC-MS (Figure S7) for the presence of ions related to janustatin A (**1**, molecular formula C_25_H_37_NO_6_). In all three mutants, production of **1** was either abolished (Δ*janA*, Δ*janAB*) or reduced to trace amounts (Δ*janB*) to give rise to new major compounds. For the double deletion mutant Δ*janAB*, the data suggested a compound missing one oxygen and having gained two hydrogens, named janustatin D (**2**, C_25_H_39_NO_5_, *m*/*z* 434.2903 [M + H]^+^, Δ + 0.18 mmu). The molecular ions of the Δ*janA* product janustatin E (**3**, C_25_H_37_NO_5_, *m*/*z* 432.2742 [M + H]^+^, Δ −0.25 mmu) suggested a missing oxygen atom as compared to **1**, while the Δ*janB* product janustatin F (**4**, C_25_H_39_NO_6_, *m*/*z* 450.2863 [M + H]^+^, Δ + 1.24 mmu) appeared to have gained two additional hydrogen atoms (Figure 2, Figure S7, and S8). For complementation experiments, the deleted genes were reintroduced into the respective mutants on the broad host range vector pBMTBX-2^15^ under the control of the pBAD promoter for arabinose-inducible expression, with empty plasmids as negative controls. In each case, the introduced genes recovered the production of **1** (Figure 2, Figure S8, and S9). Unexpectedly, we detected minor amounts of **1** in Δ*janB* mutants, which might form by spontaneous oxidation of the precursor **4** (Figure 2, and 3, Figure S8, and S9).

**Figure 3:**
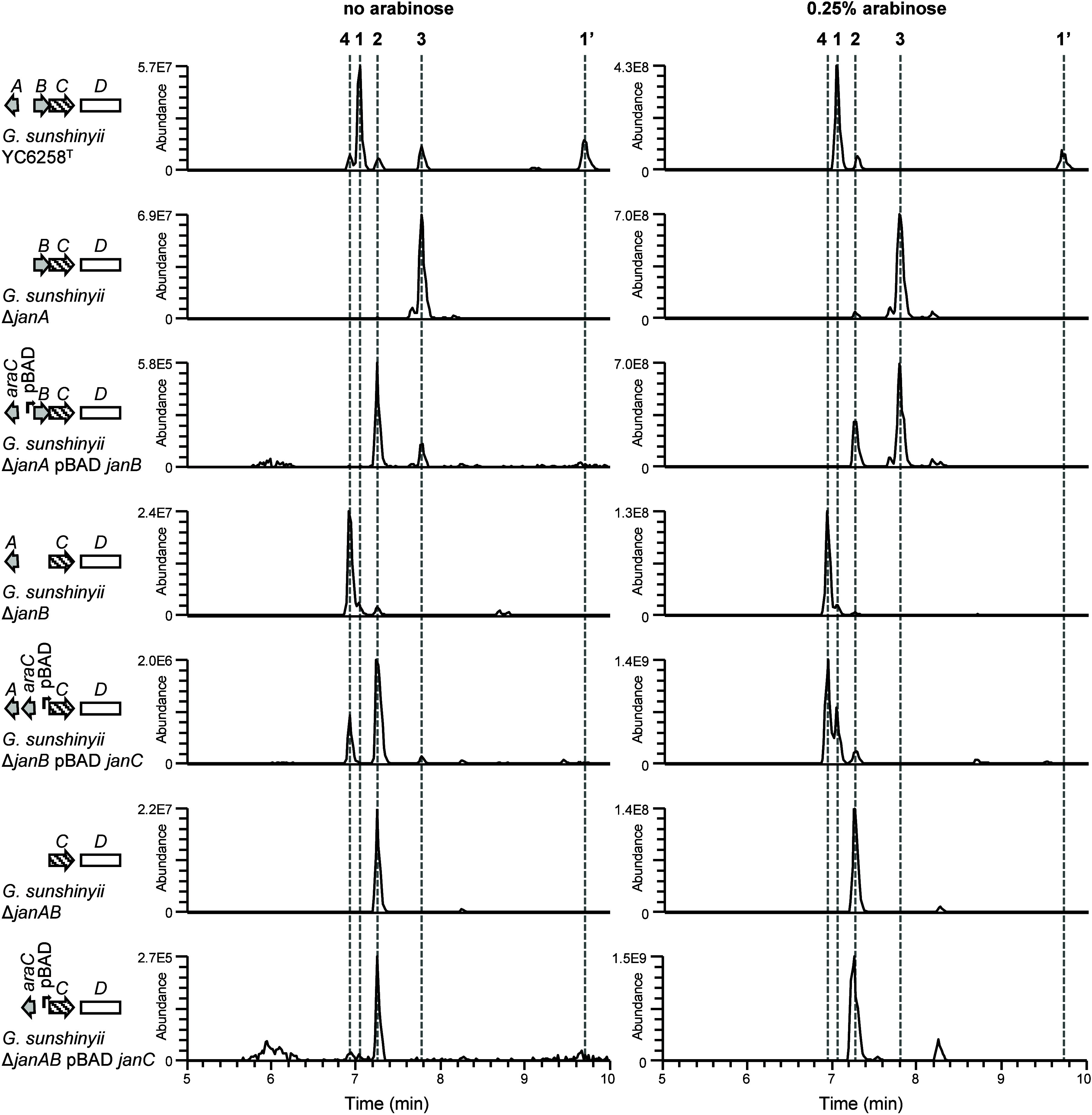
LC-MS profiles of engineered *G. sunshinyii* mutant strains with and without added arabinose. Plots are extracted ion chromatograms (*m*/*z* 432.2744, 434.2901, 448.2694, 450.2850), corresponding to intermediates **2**–**4** (compound numbers are shown above traces). The respective *jan* BGC organization (compare Figure 1) with the *araC*-pBAD promoter system is shown at the left. Experiments were performed in biological triplicates (Figure S7, and Table S7). See Figure S10 for details on janustatin B (**1**′).

To obtain structural information on the new compounds **2**-**4**, we attempted to isolate them. However, titers of the *G*. *sunshinyii* mutant strains were low at markedly reduced growth rates. To overcome this challenge, we explored ways to enhance production in this nonmodel bacterium. Natural product titers can be increased by optimizing growth conditions or by genetically modifying the producer.^16^ Since the *araC*-pBAD induction system had worked well in *G*. *sunshinyii* during the complementation experiments, we tested it in *jan* promoter exchange experiments to improve intermediate yields. Placing *araC*, facing upstream, and the pBAD promoter, facing downstream in front of *janB* or *janC* in the mutants allowed induction of biosynthetic intermediates, janustatins D-F (**2**-**4**) (Figure 3, Figure S5, and S7). Cultivation of these genetically modified strains (*G. sunhyinyii* Δ*janA* pBAD *janB*; Δ*janB* pBAD *janC;* and Δ*janAB* pBAD *janC*) at 9–18 L scale enabled the isolation of janustatins D-F (**2**-**4**).

Compound structures were elucidated by nuclear magnetic resonance (NMR) spectroscopy in combination with LC-MS (Figure 4, Figure S11–S38, Table S4, and S5). The NMR spectra of compounds **2**–**4** revealed a series of signals that were very similar to the C(1) to C(13) part of janustatin A (**1**).^3^ For **2** and **4**, correlation spectroscopy (COSY) signals from spin system **III** (Figure 4) to a downfield singlet proton signal connected to a heteroatom were observed. Heteronuclear multiple bond correlations (HMBC) signals from H(18) across this heteroatom to C(15) suggested a nitrogen next to C(18). The signals of a proton connected to a nitrogen and the deshielded chemical shift of C(20), δ_C_ 188 ppm, suggests the C(20) keto tautomer to be the prominent solution structure of compounds **2** and **4** (Figure 4). Assignment of the other shifts in the bicyclic ring was difficult for all three compounds due to the lack of protons in that region. Correlations of H(13), H(14) and H(18) to C(15) as well as correlations of H(14) and H(19) to C(16) established the nitrogen heterocycle as a six-membered ring. In none of the spectra, other than in one NMR measurement of **3** that was recorded before the purification process was finished, correlations to the carbonyl C(17) were observed. However, elucidation of C(1)–C(16) and C(18)–C(20) and comparison to the suggested molecular formulas based on HRMS analysis only left one carbon and one oxygen to be added to molecules (**2**-**4**). These were placed between the quaternary carbon C(16), which was still missing a bonding partner, and the oxygen of C(13), forming a δ-lactone for **2**-**4** (Figure 4). This structure also fits the downfield chemical shift of H(13) in all three compounds. Additionally, in tandem LC-MS experiments, we were able to detect fragment ions corresponding to the respective bicyclic systems of compounds **2**-**4** (Figure S38). The complementation experiments, combined with the elucidation of structures **2**-**4** corroborate the hypothesis that JanA and JanB are responsible for maturation of the assembly line intermediate to the final natural product **1** (Figure 4). For other natural products, similar late-stage modifications have been reported.^17^ For example, in jerangolid A biosynthesis, two Rieske oxygenases JerL and JerP, together with the flavin-dependent reductase JerO, install a hydroxy group and a double bond, respectively.^18^ Furthermore, cytochrome P450 enzymes are known to catalyze similar transformations,^19^ of which labrenzin hydroxylations^20^ and the introduction of a benzene unit in lorneic acids^21^ are recent examples.

**Figure 4:**
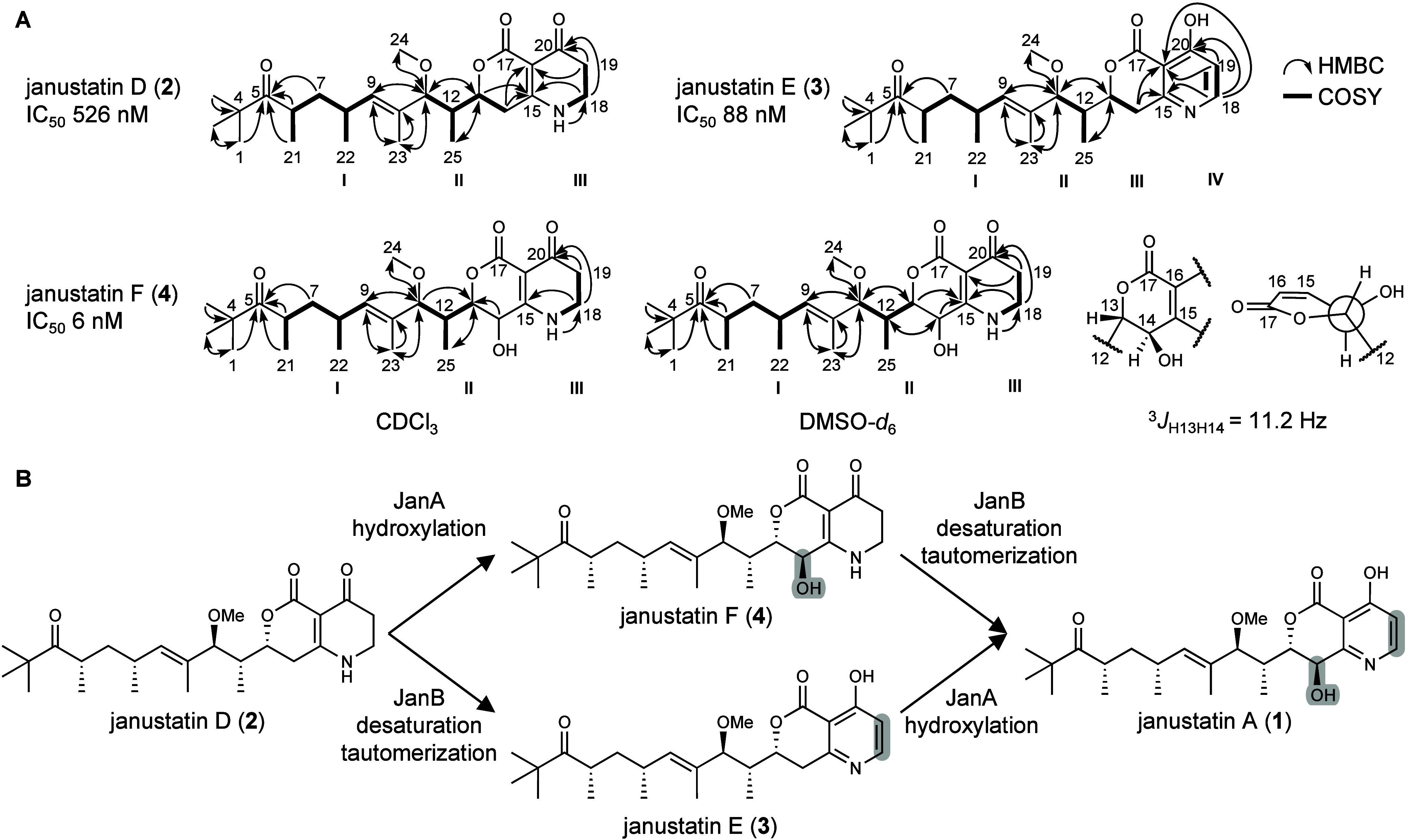
Structure elucidation of janustatins D-F (**2**-**4**). **A**, Core COSY spin-systems and HMBC correlations. Based on the molecular formula, the carbonyl C(17) was placed next to C(16) forming a δ-lactone, in agreement with the downfield shift of C(13). Details on structure elucidation and bioactivity assays can be found in the Supporting Information. **B**, Updated biosynthetic janustatin model, in which JanA catalyzes hydroxylation, JanB desaturation of the assembly line product **2**. The two enzymes can act independently of each other.

The isolation of janustatins D-F (**2**-**4**) shows that promoter replacement is a useful genetic engineering approach to produce polyketides in *G*. *sunshinyii*. To quantify the relative impact of the optimizations on **1**-**4** titers in the mutant strains, we prepared external standard curves using the isolated polyketides (Figure S7, and S39, Table S6-Table S8). Adding *araC* and the pBAD promoter upstream of *janC* in the Δ*janB* and Δ*janAB* deletion mutants enhanced production of **4** and **2**, respectively, by about 10-fold (Figure 3, Figure S7, Table S7). Adding the promoter upstream of *janB* in the Δ*janA* mutant yielded compound **3** in comparable titers to the strain with the native promoter. In summary, we were able to increase biosynthetic intermediate titers in the mutants to levels comparable to the natural product in wild-type *G*. *sunshinyii*: **1** in the wild type: 0.7 mg/L; **2** in Δ*janAB* pBAD *janC*: 0.6 mg/L; **3** in Δ*janA* pBAD *janB*: 0.4 mg/L; **4** in Δ*janB* pBAD *janC*: 0.7 mg/L (Table S7). Our attempts to increase janustatin A (**1**) production by promotor replacement and BGC refactoring yielded strains with product titers comparable to those of the wild type (Figure S42, and S43, Table S9). The observation that arabinose increases polyketide titers was consistent for all three deletion strains Δ*janA,* Δ*janB*, and Δ*janAB*, as well as for the wild type. Strains with the janustatin BGC under pBAD control produced multiple orders of magnitude less of compounds **1**-**4** when cultivated without arabinose (5843-fold decrease of **2** production in Δ*janAB* pBAD *janC*, 5594-fold decrease of **3** production in Δ*janA* pBAD *janB*, 3396-fold decrease of **4** production in Δ*janB* pBAD *janC*, Table S7). Thus, the pBAD promoter provides good control over polyketide production in *G*. *sunshinyii*.

With sufficient amounts of compounds **1**-**4** at hand, we set out to study the effect of the modifications installed by JanA and JanB on the cytotoxicity of janustatins. Janustatin A (**1**) is extremely cytotoxic with an IC_50_ of 0.8 nM against Henrietta Lacks (HeLa) cervical cancer cells.^3^ When tested against the same cell line, IC_50_ values of janustatin E (**3**) and janustatin F (**4**) were 88 and 6 nM, respectively (Figure 4, Figure S40), corresponding to a 106- and 7-fold decrease in cytotoxicity compared to **1**. Janustatin D (**2**) displayed an even lower cytotoxicity with an IC_50_ of 526 nM. These results show that hydroxylation of janustatin at C(14) and aromatization to the pyridine ring greatly enhance cytotoxicity, with a combination of the two modifications resulting in a synergistic increase of toxicity. Taken together, these results identify the bicyclic system as an important pharmacophore of janustatin A (**1**). The proteins JanA and JanB are two modifying enzymes involved in heterocycle maturation that render the initial assembly line product more toxic.

In conclusion, this work identifies the final biosynthetic steps of janustatin A (**1**), an exceptionally potent cytotoxin produced by a plant symbiont. We show that the unusual bicyclic pyridine-containing moiety of **1** is generated by the desaturase-like enzymes JanA and JanB through hydroxylation and aromatization of the PKS-NRPS product janustatin D (**2**). Since the earliest post-PKS-NRPS intermediate **2** from the *janAB* deletion mutant already contains all heterocycle atoms at the correct position, the reversal of biosynthetic units likely occurs on the assembly line, with a potential mechanism involving β-branching and rearrangement, as proposed in Figure 1. While the modifications introduced by JanA and JanB are required for full bioactivity, janustatin E (**3**) lacking one chiral center still displays double-digit nanomolar cytotoxicity. These insights, together with current studies on the cellular target, might aid the design of further upscaled production routes for active compounds through combined chemical and enzymatic synthesis using the hydroxylase JanA.

## Data Availability

The data supporting the findings of this study are available in this Article and the Supporting Information. Data that support the claims in this manuscript are available on the Zenodo repository (10.5281/zenodo.14035836).

## Supporting Information Available

Experimental details, sequence alignments, LC-MS chromatograms, NMR spectra (PDF)

## Accession Codes

The genome sequence of *G. sunshinyii* YC6258 is accessible in GenBank under accession no. NZ_CP007142.1, the janustatin gene cluster is located at locus YC6258_05439 (AJQ97469.1) to YC6258_05446 (AJQ97476.1). The janustatin BGC has been deposited in MIBiG under accession no. BGC0002136. GenBank Protein IDs; JanA: WP_052830556.1, JanB: WP_052830555.1, JanC: WP_044619205.1, JanD: WP_044619203.1, JanE: WP_052830554.1.
