# Supplementary material for: Insights into Heterocycle Biosynthesis in the Cytotoxic Polyketide Alkaloid Janustatin A from a Plant-Associated Bacterium
Source: Biochemistry. 2025 Jan 9;64(2):357–63. doi: 10.1021/acs.biochem.4c00542 (PMC11755721; doi:10.1021/acs.biochem.4c00542)
Supplement: Supplementary file 1 — bi4c00542_si_001.pdf [file bi4c00542_si_001.pdf]

Supporting Information for

Insights into Heterocycle Biosynthesis in the Cytotoxic Polyketide Alkaloid Janustatin A from a Plant-Associated Bacterium

AUTHOR NAMES. Stefan Leopold-Messer[1], Pornsuda Chawengrum[1,2], Jörn Piel[1]*

AUTHOR ADDRESS.

[1]Institut für Mikrobiologie, Eidgenössische Technische Hochschule (ETH) Zürich, Vladimir-Prelog-Weg 4, 8093 Zürich, Switzerland

[2]Chemical Biology Program, Chulabhorn Graduate Institute, Chulabhorn Royal Academy, Kamphaeng Phet 6 Road, Laksi, Bangkok 10210, Thailand

**Corresponding author's Email: jpiel@ethz.ch*

MATERIALS AND METHODS

General

High performance liquid chromatography–heated electron spray ionization–mass spectrometry (HPLC-HESI-MS) was performed on a Thermo Scientific Q Exactive or LTQ Orbitrap XL mass spectrometer coupled to a Dionex Ultimate 3000 UPLC system operated by Xcalibur 4.1 and Chromeleon Xpress 7.2 (Thermo Scientific) respectively. NMR spectra were recorded on a Bruker Avance III spectrometer equipped with a cryoprobe at 500 MHz and 600 MHz for ^{1}H NMR and 125 MHz and 150 MHz for ^{13}C NMR at 298 K operated by TopSpin 3.5/4.1 (Bruker). Chemical shifts were referenced to the solvent peaks at δ_H 2.50 ppm and δ_C 39.51 ppm for DMSO-d_6 and δ_H 7.26 ppm and δ_C 77.16 ppm for chloroform-*d*. Data was analysed using Xcalibur 4.1, TopSpin 4.1 and MestReNova (Mestrelab Research). Purification of natural products was achieved on Agilent Infinity 1260 HPLCs operated by Open Lab CDS 2.2 (Agilent). Eukaryotic cell lines were not authenticated as they were used to test cytotoxic effects of compounds. Oligonucleotide synthesis and sequencing was performed by Microsynth AG. *Gynuella sunshinyii* YC6258^{T} was obtained from NITE Biological Resource Center (NBRC).[1]

Construction of gene deletion plasmids (pEB17-Δ*janA*, pEB17-Δ*janB* and pEB17-ΔNRPS-para261)

Primers for Gibson assemblies were designed using NEBuilder (https://nebuilder.neb.com/#!/) to generate products with 20 bp overhang to one another. For gene deletion constructs, 500 bp up- and downstream of the targets (*janA*, *janB*, and the NRPS-para261 domain) in the genome of *G. sunshinyii* and the plasmid pSW8197[2] were used as queries (Table S1, primers 1-18). To amplify target genes, a small culture was transferred to a 1.5 mL Eppendorf tube, sonicated for 10 min and then boiled at 100 °C for 10 min. The supernatant was used as a template in the PCR. Purified plasmid was used as a template for the plasmid backbone (pSW8197). PCR products were separated on an agarose gel and purified with a NucleoSpin Gel and PCR Clean-up kit (Macherey-Nagel AG). The homology arms were fused by an overlap extension PCR (outer primers of the fused product were only added after 15 PCR steps). After Gibson assembly (homemade mixture)[3] of the fragments, plasmids were electroporated into *E. coli* DH5α pir, recovered in Luria-Bertani (LB) broth for one hour and plated onto LB containing kanamycin (50 µg/mL, 1% (w/v) glucose). A culture of a single colony was prepared, plasmid isolated with a NucleoSpin Plasmid kit (Macherey-Nagel AG) and sequenced.

The counterselection of pSW8197 is based on induction of the toxic gene *ccdB* by arabinose. This marker was difficult to handle due to high mutation rates that resulted in poor reproducibility (Figure S4). Therefore, we changed to another plasmid backbone, pEB17.[4] Primers for backbone exchange were designed (primers 19-22), plasmids were constructed and purified as described above, yielding the plasmids pEB17-Δ*janA*, pEB17-Δ*janB* and pEB17-Δpara (Table S1).

Construction of gene activation plasmids (pEB17-pBAD *janB*, pEB17-Δ*janB* pBAD *janC*)

The procedure used to create gene activation plasmids diverged from that for gene deletion plasmids only in a minor design difference. Additionally, *araC*, facing upstream, and the pBAD promoter, facing downstream were placed in between the two homology arms. *G. sunshinyii*, pBMTBX-2 and pEB17 served as PCR templates. The first homology arm, *araC* and the pBAD promoter, and the second homology arm were fused by overlap extension PCR. This PCR product was then used in a Gibson assembly with the pEB17 backbone (primers 23-34).

Construction of pBMTBX-2-*janA* and pBMTBX-2-*janB*

Primers for Gibson assemblies were designed using NEBuilder (https://nebuilder.neb.com/#!/) to generate products with 20 bp overhang to one another. The genes *janA* and *janB* and the plasmid pBMTBX2 were used as queries (Table S1, primers 35-42). To amplify target genes, a small culture was transferred to a 1.5 mL Eppendorf tube, sonicated for 10 min and then boiled at 100 °C for 10 min. The supernatant was used as a template in the PCR. Purified plasmid was used as a template for the plasmid backbone (pBMTBX-2). PCR products were separated on an agarose gel and purified with a NucleoSpin Gel and PCR Clean-up kit (Macherey-Nagel AG). After Gibson assembly of the fragments, plasmids were electroporated into *E. coli* DH5α pir, recovered in Luria-Bertani (LB) broth for one hour and plated onto LB containing kanamycin (50 µg/mL). A culture of a single colony was prepared, plasmid isolated with a NucleoSpin Plasmid kit (Macherey-Nagel AG) and sequenced (primer 50).

Transformation of *E. coli* ST18 donor strains

The plasmids (backbones pEB17 and pBMTBX-2) were electroporated into the auxotrophic donor strain *E. coli* ST18.[5] After bacteria were recovered for one hour in LB supplemented with 50 µg/mL 5-aminolevulinic acid (ALA), cultures were plated onto LB (50 µg/mL ALA, 50 µg/mL kanamycin). For the conjugation a culture of a single colony was prepared.

Construction of *G. sunshinyii* mutants by conjugation and homologous recombination

The following *G. sunshinyii* mutants were constructed using the same protocol: Δ*janA,* Δ*janB,* Δ*janAB,* Δ*para,* Δ*janA* pBAD *janB*, Δ*janB* pBAD *janC,* Δ*janAB* pBAD *janC*. Table S2 lists which donor and acceptor strains were used for the construction of each respective mutant.

Cultures of donor strain (*E. coli* ST18 carrying one of the plasmids in Table S2) were prepared in LB (50 µg/mL ALA, 50 µg/mL kanamycin). Cultures of acceptor strain (one of the *G. sunshinyii* mutants in Table S2) were prepared in marine broth (½ MB: 5 g bacteriological peptone, 1 g yeast extract, 16.5 g instant ocean (Aquarium Systems), 1 L water). The next day, 10 mL of *E. coli* cultures and 50 mL of *G. sunshinyii* cultures were harvested in falcon tubes by centrifugation. The cells were resuspended in 10 mL of the respective medium (LB or ½ MB), the optical density at a wavelength of 600 nm ($OD_{\lambda=600\ nm}$) was measured and after another centrifugation step the cells were resuspended in the respective medium to yield a suspension of $OD_{\lambda=600\ nm}$ = 4. Donor and acceptor strain were mixed in three different ratios (1:9, 3:7, 1:1) to a total volume of 1 mL. After centrifugation, 800 µL of the supernatant were discarded, the remaining volume was used to resuspend the cell mixtures. A plate (½ MB, 50 µg/mL ALA) was separated into three segments and the three different ratios were dropped onto a single spot in one of the segments. After one day of growth at 37 °C, half of each of the three spots was combined and resuspended in 500 µL 0.9% (w/v) NaCl solution, of which 200 µL were plated onto ½ MB agar (50 µg/mL kanamycin) and distributed using a cell spreader. Single colonies were isolated, a liquid culture (½ MB, 50 µg/mL kanamycin) prepared, and the genomic integration of the suicide plasmid confirmed by PCR with two primer pairs. Two of the primers were designed to bind in the plasmid facing up- or downstream (Table S1, primers 43, 44), the other two to bind up- or downstream of the desired integration site (Table S1, primers 45-49). Then, 5 mL of ½ MB without any additives were inoculated with 100 µL of the *G. sunshinyii* culture and cultivated for one day at 30 °C. The next day, 200 µL of this culture were plated onto ½ MB containing 10% (w/v) sucrose. After two days of growth at 30 °C, about 30 colonies were picked using a toothpick and first a plate of ½ MB (50 µg/mL kanamycin) then a plate of ½ MB was inoculated. Colonies that grew on antibiotics were considered false positives and 10 colonies that only grew on ½ MB were propagated into liquid culture (½ MB). Successful genetic modification was confirmed using primers that bind up- and downstream of the modification site (Figure S5, Table S1, primers 45-49). PCR products were separated on an agarose gel, purified with a NucleoSpin Gel and PCR Clean-up kit (Macherey-Nagel AG) and sequenced.

Conjugation of pBMTBX-2 plasmids

Cultures of *E. coli* ST18 carrying empty pBMTBX-2, pBMTBX-2-*janA* and pBMTBX-2-*janB* were prepared in LB (50 µg/mL ALA, 50 µg/mL kanamycin). Cultures of *G. sunshinyii* mutants Δ*janA* and Δ*janB* were prepared in marine broth (½ MB). The next day, 10 mL of *E. coli* cultures and 50 mL of *G. sunshinyii* cultures were harvested in falcon tubes by centrifugation. The cells were resuspended in 10 mL of the respective medium (LB or ½ MB), the $OD_{\lambda=600\ nm}$ was measured and after another centrifugation step the cells were resuspended in the respective medium to yield a suspension of $OD_{\lambda=600\ nm}$ = 4. *E. coli* ST18 carrying empty pBMTBX-2 or pBMTBX-2-*janA* were mixed with *G. sunshinyii* Δ*janA* and *E. coli* ST18 carrying empty pBMTBX-2 or pBMTBX-2-*janB* were mixed with *G. sunshinyii* Δ*janB* in different ratios of donor to acceptor strain (1:9, 3:7, 1:1) to a total volume of 1 mL. After centrifugation, 800 µL of the supernatant were discarded, the remaining volume was used to resuspend the cell mixtures. A plate (½ MB, 50 µg/mL ALA) was separated into three segments and the three different ratios were dropped onto a single spot in one of the segments. After one day of growth at 37 °C, half of each of the three different spots was resuspended in 500 µL 0.9% (w/v) NaCl solution, of which 200 µL were plated onto ½ MB agar (50 µg/mL kanamycin). Single colonies were isolated to confirm the genotype of the four new mutant strains (*G. sunshinyii* Δ*janA* with empty pBMTBX-2 or pBMTBX-2-*janA* and *G. sunshinyii* Δ*janB* with empty pBMTBX-2 and pBMTBX-2-*janB*) by PCR (Figure S5, primers 45/47, 46/47 and 50/51).

LC-HRMS analysis of *G. sunshinyii* mutants: complementation experiments

The following *G. sunshinyii* mutants were analyzed using the same protocol: wild type with pBMTBX-2-empty, Δ*janA* with pBMTBX-2-empty or *janA*, Δ*janB* with pBMTBX-2-empty or *janB* in 5 mL ½ MB (50 µg/mL kanamycin) with and without 0.25% (w/v) arabinose, in biological triplicates. A preculture was used to inoculate 5 mL of the cultures to a calculated OD $_{\lambda=600\ nm}$ = 0.004 in 15 mL falcon tubes. After three days of growth at 30 °C, 180 rpm the supernatants were extracted with 10 mL ethyl acetate and the organic phase was dried. The residue was dissolved in 300 µL acetonitrile and analyzed by LC-HRMS.

LC-MS analysis of *G. sunshinyii* mutants: gene deletion and activation experiments

The following *G. sunshinyii* mutants were analyzed using the same protocol: wild type, Δ*janA*, Δ*janB*, Δ*janAB*, Δ*janA* pBAD *janB*, Δ*janB* pBAD *janC*, Δ*janAB* pBAD *janC*, and Δ*para*. Cultures were cultivated in ½ MB. Every strain was grown without and with 0.25% (w/v) arabinose, each in biological triplicates. In 100 mL Erlenmeyer flasks 15 mL of liquid medium was inoculated with preculture to a calculated OD $_{\lambda=600\ nm}$ = 0.005. After three days of growth at 30 °C, 180 rpm 10 mL of the supernatant were extracted with 10 mL ethyl acetate and the organic phase was dried. The residue was dissolved in 300 µL acetonitrile and analyzed by LC-HRMS.

Relative quantification of janustatins in mutants

Standards of janustatins A, D, E, F with seven concentrations spanning a range of approximately 100 µM to 10 nM were prepared (see Table S6). These standards and the mutant extracts (see above) were analyzed by LC-MS (injection volume: 10 µL). The extracted ion chromatograms (EICs) at *m/z* 432.2744, 434.2901, 448.2694, 450.2850 were exported (see FigureS6-S8) and a python script was used to integrate the peaks at the respective retention times (janustatin A: 7.02-7.15 min, janustatin D: 7.20-7.40 min, janustatin E: 7.70-7.90 min, janustatin F: 6.87-6.96 min). Both a linear as well as a second order polynomial model were used to fit the data (Figure S39, Table S6). Because the residual sum of squares was lower for the polynomial model, it was used to determine the amount of janustatins (**1**-**4**) in the mutant cultures.

The EICs of mutants were integrated at the above-mentioned retention times. Using the determined polynomial function (Table S6) the amounts were calculated and multiplied by the dilution factor: 0.3 mL acetonitrile were used to dissolve the residue extracted from 10 mL culture of the mutants Δ*janA*, Δ*janB*, Δ*janAB*, Δ*para*, Δ*janA* pBAD *janB*, Δ*janB* pBAD *janC*, Δ*janAB* pBAD *janC* (Table S7); and 5 mL culture for the mutants carrying pBMTBX-2 plasmids (Table S8).

Optimization of arabinose induction pBMTBX-2

G. sunshinyii Δ*janA* carrying pBMTBX-2 with or without *janA* mutant were cultivated in 20 mL ½ MB (50 µg/mL kanamycin) without arabinose or supplemented with 0.025% and 0.25% (w/v) arabinose. After 3 days at 30 °C, 180 rpm the supernatant was extracted three times with ethyl acetate and dried. Residues were dissolved in acetonitrile and analysed by LC-MS.

Isolation of janustatin D (2)

A total of 12 L *G. sunshinyii* Δ*janAB* pBAD *janC* was cultivated in batches of 300 mL PH-103[6] broth (tryptone 20 g, yeast extract 5 g, glucose 4 g, maltose 4 g, $CaCO_3$ 4 g, and instant ocean salts 27 g in 1 L water) with 0.25% (w/v) arabinose in 1 L Erlenmeyer flasks at 30 °C for 3 days on an orbital shaker (150 rpm). The cultures were centrifuged, the supernatant was extracted three times with ethyl acetate, and dried to obtain 1.1 g of crude extract. The extract was separated by RP-HPLC (Phenomenex Luna 5µ C18, φ 20 x 250 mm, 10.0 mL/min, 200 nm) with MeCN in H_2O + 0.1% formic acid elution, starting from isocratic 5% MeCN for 5 min, gradient from 5% to 95% MeCN for 32 min, and isocratic elution 95% MeCN for 10 min to afford 40 fractions. The fractions at a retention time (tR) of 26 to 29 min (27.0 mg) were combined and repurified by semi preparative RP-HPLC (Phenomenex Luna 5µ Phenyl-Hexyl, φ 10 x 250 mm, 2.0 mL/min, 200 nm) with 40% MeCN + 0.1% formic acid. Peaks at a tR of 31 to 33 min (4.0 mg) were combined and purified by semi preparative RP-HPLC (Phenomenex Synergi 4µ Hydro-RP, φ 10 x 250 mm, 2.0 mL/min, 200 nm) with 37% MeCN + 0.1% formic acid to obtain 0.44 mg of janustatin D (**2**), tR 51.8 min.

Isolation of janustatin E (3)

A total of 9 L *G. sunshinyii* Δ*janA* pBAD *janB* was cultivated in batches of 300 mL PH-103 broth with 0.25% (w/v) arabinose in 1 L Erlenmeyer flasks at 30 °C for 3 days on an orbital shaker (150 rpm). The cultures were centrifuged, the supernatant was extracted three times with ethyl acetate, and dried to obtain 935 mg of crude extract. The extract was fractionated by RP-HPLC

(Phenomenex Luna 5µ C18, φ 20 x 250 mm, 10.0 mL/min, 200 nm) with MeCN in H_2O + 0.1% formic acid elution starting from isocratic 5% MeCN for 5 min, gradient from 5% to 95% MeCN for 32 min, and isocratic elution 95% MeCN for 10 min to afford 40 fractions. The fractions at a tR of 28 to 31 min (19.5 mg) were combined and repurified by semi preparative RP-HPLC (Phenomenex Luna 5µ Phenyl-Hexyl, φ 10 x 250 mm, 2.0 mL/min, 200 nm) with 40% MeCN + 0.1% formic acid. Peaks at a tR of 32 to 35 min (3.5 mg) were combined and purified by analytical RP-HPLC (Phenomenex Synergi 4µ Hydro-RP, φ 4.6 x 250 mm, 2.0 mL/min, 200 nm) with 38% MeCN + 0.1% formic acid to obtain 0.18 mg of janustatin E (**3**), tR 20.6 min.

Isolation of janustatin F (4)

A total of 16 L *G. sunshinyii* Δ*janB*, pBAD *janC* was cultivated in batches of 300 mL PH-103 broth with 0.25% (w/v) arabinose in 1 L Erlenmeyer flasks at 30 °C for 3 days on an orbital shaker (150 rpm). The cultures were centrifuged, the supernatant was extracted three times with ethyl acetate, and dried to obtain 1.9 g of crude extract. The extract was fractionated by RP-HPLC (Phenomenex Luna 5µ C18, φ 20 x 250 mm, 10.0 mL/min, 200 nm) with MeCN in H_2O + 0.1% formic acid elution starting from isocratic 5% MeCN for 5 min, gradient from 5% to 95% MeCN for 32 min, and isocratic elution 95% MeCN for 10 min to afford 40 fractions. The fractions at a tR of 25 to 30 min (65 mg) were combined and repurified by semi preparative RP-HPLC (Phenomenex Luna 5µ Phenyl-Hexyl, φ 10 x 250 mm, 2.0 mL/min, 200 nm) with 40% MeCN + 0.1% formic acid. Peaks at a tR 24 to 27 min (3.2 mg) were combined and purified by analytical RP-HPLC (Phenomenex Synergi 4µ Hydro-RP, φ 4.6 x 250 mm, 2.0 mL/min, 200 nm) with 37% MeCN + 0.1% formic acid to obtain 0.89 mg of janustatin F (**4**), tR 11.5 min.

Structure elucidation of janustatin D (2)

Janustatin D (**2**) had the predicted molecular formula of $C_{25}H_{39}NO_5$ based on HRMS (*m/z* 434.2903 $[M+H]^+$, Δ +0.18 mmu (Figure S38). ^{1}H NMR in conjunction with heteronuclear single quantum coherence (HSQC) data suggested three doublet methyls, three singlet aliphatic methyls, one vinylic methyl, one methoxy group, one proton connected to an sp^2 carbon, two oxymethines, four methylene, and three methines (Figure S11-16). The HMBC revealed seven additional quaternary carbons, adding up to a total of 24 detected carbons (Figure S19, and S20).

From the COSY spectrum, three units **I-III** were deduced (Figure 4, Figure S17, S18). A *t*-butyl group was assigned to C(1)-C(4), which connected to unit **I** via a ketone C(5) by HMBC correlations from H(1/2/3) to C(1/2/3), C(4) and C(5), from H(6), H(7), and from H(21) to C(5) (Figure S19, S20). Units **I** and **II** were connected by HMBC correlations from H(9) and H(23) to C(11), from H(11) to C(9) and C(23), and from H(8), H(23) and H(11) to C(10). The chemical shift of the vinylic methyl C(23) at δ_C 10.1 ppm in $CDCl_3$ suggests *9E* configuration of the olefinic bond (Figure S15, S16). The methoxy group was attached to C(11) by HMBC correlations from H(11) to C(24) and from H(24) to C(11). In addition to a weak COSY correlation between H(12) and H(13), HMBC correlations from H(25) to C(11), C(12) and C(13) as well as HMBC correlations from H(11) to C(13) and H(13) to C(11) established unit **II**. Finally, HMBC correlations from H(13) and H(14) to a quaternary carbon C(15) (δ_C 168.3 ppm in $CDCl_3$) were observed.

The chemical shift of H(18) in **III** (δ_H 3.51 ppm in DMSO-d_6, δ_H 3.75 ppm in $CDCl_3$) suggested connectivity to a heteroatom. The COSY spectrum revealed a correlation from H(18) to a singlet proton on a heteroatom, possibly a N-H (δ_H 9.00 ppm in DMSO-d_6, δ_H 7.88 ppm in $CDCl_3$). This singlet N-H showed HMBC correlations to C-(18). Furthermore, the two methylenes in unit **III**, H(18) and H(19), were extended by a quaternary carbon carrying an oxygen based on HMBC correlations from H(18) and H(19) to C(20) and its downfield chemical shift at δ_C 187.9 ppm in $CDCl_3$. HMBC correlations from H(19) to an additional quaternary carbon at δ_C 96.9 ppm in $CDCl_3$ attached C(16) to unit **III**. With 24 carbons, 39 hydrogens, 4 oxygens and 1 nitrogen (6 relative double bond equivalents) accounted for, only one fully oxidized carbon C(17) at δ_C 161.4 ppm in DMSO-d_6 (Figure S14) and one oxygen remained to complete the suggested molecular formula ($C_{25}H_{39}NO_5$).

HMBC correlations from H(13), H(14) and H(18) to a non-protonated sp^2 carbon, C(15), connected units **II** and **III**. The connection of C(13) to C(15) via an oxygen, thereby forming a carbamate was excluded because it would require a quaternary nitrogen. Therefore, C(15) was placed next to C(14) connecting units **II** and **III** (Figure 4). Based on the chemical shift of C(16) at δ_C 95.9 ppm in $CDCl_3$ it was connected to the two remaining carbons, C(15) and C(17) and not the oxygen of unit **II**. To keep the constraints of the suggested molecular formula, C(17) was connected to C(13) *via* a lactone. This cyclization is in agreement with the downfield shift of the oxymethine H(13) and concluded the structure elucidation.

Structure elucidation of janustatin E (3)

Janustatin E (**3**) had the predicted molecular formula of $C_{25}H_{37}NO_5$ based on HRMS (*m/z* 432.2742 $[M+H]^+$, Δ -0.25 mmu (Figure S38). ^{1}H NMR in conjunction with HSQC data suggested three doublet methyls, three singlet aliphatic methyls, one vinylic methyl, one methoxy group, three protons connected to an sp^2 carbon, two oxymethines, two methylene and three methines (Figure S21-S24). The HMBC revealed seven additional quaternary carbons, adding up to a total of 25 detected carbons (Figure S26). From the COSY spectrum, three units **I-IV** were deduced (Figure S25). A *t*-butyl group was assigned to C(1)-C(4), which connected to unit **I** via a ketone C(5) by HMBC correlations from H(1/2/3) to C(1/2/3), C(4) and C(5), from H(6), H(7), and from H(21) to C(5) (Figure S26). Units **I** and **II** were connected by HMBC correlations from H(9) and H(23) to C(11), from H(11) to C(9) and C(23), and from H (8), H(23) and H(11) to C(10). The chemical shift of the vinylic methyl C(23) at δ_C 10.1 ppm in $CDCl_3$ suggests *9E* configuration of the olefinic bond (Figure S23, S24). The methoxy group was attached to C(11) by HMBC correlations from H(11) to C(24) and from H(24) to C(11). In addition to a COSY correlation between H(13) and H(14), HMBC correlations from H(25) to C(11), C(12) and C(13), and H(11) to C(13) as well as HMBC correlations from H(13) to C(11) and H(14) to C(12) connected units **II** and **III**.

The chemical shift of H(18) at δ_H 8.42 ppm in $CDCl_3$ suggested connectivity to a heteroatom. In addition, the two aromatic peaks in unit **III** were extended by a quaternary carbon carrying an oxygen based on HMBC correlations from H(18) and H(19) to C(20) and its downfield chemical shift at δ_C 167.8 ppm in $CDCl_3$. HMBC correlations from H(19) to an additional quaternary carbon at δ_C 106.3 ppm in $CDCl_3$ attached C(16) to unit **III**. With 24 carbons, 37 hydrogens, 4 oxygens and one nitrogen (7 relative double bond equivalents) accounted for, only one fully oxidised carbon C(17) and one oxygen remained to complete the suggested molecular formula ($C_{25}H_{37}NO_5$).

HMBC correlations from H(13), H(14) and H(18) to a non-protonated sp^2 carbon, C(15), connected units **II** and **III**. The connection of C(13) to C(15) via the oxygen to form a carbamate was excluded because it would require a quaternary nitrogen. Therefore, C(15) was placed next C(14) connecting units **III** and **IV** (Figure 4). Based on the chemical shift of C(16) at δ_C 106.3 ppm in $CDCl_3$ it was connected to the two remaining carbons, C(15) and C(17) and not the oxygen of C(13). To keep the constraints of the suggested molecular formula, C(17) was connected to C(13) *via* a lactone. This cyclization is in agreement with the downfield shift of the oxymethine H(13).

An impurity peak was observed at δ_H 5.37 ppm in $CDCl_3$. NMR spectra of **3** were also recorded in DMSO-d_6, but some key correlations could not be detected in that solvent.

Structure elucidation of janustatin F (4)

Janustatin F (**4**) had the predicted molecular formula of $C_{25}H_{39}NO_6$ based on HRMS (*m/z* 450.2863 $[M+H]^+$, Δ +1.24 mmu (Figure S38). ^{1}H NMR in conjunction with HSQC data suggested three doublet methyls, three singlet aliphatic methyls, one vinylic methyl, one methoxy group, one proton connected to an sp^2 carbon, three oxymethines, three methylene, three methines (Figure S27-32). The HMBC revealed six additional quaternary carbons, adding up to a total of 24 detected carbons (Figure S35, S36). From the COSY spectrum, three units **I-III** were deduced (Figure 4, Figure S33, S34). A *t*-butyl group was assigned to C(1)-C(4), which connected to unit **I** via a ketone C(5) by HMBC correlations from H(1/2/3) to C(1/2/3), C(4) and C(5), from H(6), H(7), and from H(21) to C(5) (Figure S35, 36). Units **I** and **II** were connected by HMBC correlations from H(9) and H(23) to C(11), from H(11) to C(9) and C(23), and from H (8), and H(23) to C(10). The chemical shift of the vinylic methyl C(23) at δ_C 10.1 ppm in $CDCl_3$ suggests *9E* configuration of the olefinic bond (Figure S31, 32). The methoxy group was attached to C(11) by HMBC correlations from H(11) to C(24) and from H(24) to C(11). In addition to a weak COSY correlation between H(12) and H(13), HMBC correlations from H(25) to C(11), C(12) and C(13) as well as HMBC correlations from H(11) to C(13) established unit **II**.

The chemical shift of H(18) (δ_H 3.55 ppm in DMSO-d_6, δ_H 3.76 ppm in $CDCl_3$) suggested connectivity to a heteroatom. The COSY spectrum revealed a correlation from H(18) to a singlet on a heteroatom, possibly the N-H (δ_H 8.83 ppm in DMSO-d_6, δ_H 7.86 ppm in $CDCl_3$). Furthermore, the two methylenes in unit **III** were extended by a quaternary carbon carrying an oxygen based on HMBC correlations from H(18) and H(19) to C(20) and C(20)s downfield chemical shift (δ_C 186.3 ppm in DMSO-d_6, δ_C 188.0 ppm in $CDCl_3$). HMBC correlations from H(19) to an additional quaternary carbon (δ_C 92.3 ppm in DMSO-d_6) attached C(16) to unit **III**.

With 24 carbons, 39 hydrogens, 4 oxygens and one nitrogen (6 relative double bond equivalents) accounted for, only one fully oxidised carbon C(17) and one oxygen remained to complete the suggested molecular formula ($C_{25}H_{39}NO_6$).

HMBC spectrum in DMSO-d_6 showed the correlation from H(13), H(14) and H(18) to a non-protonated sp^2 carbon, C(15), connected units **II** and **III**. The connection of C(13) to C(15) via the oxygen to form a carbamate was excluded because it would require a quaternary nitrogen. Therefore, C(15) was placed next C(14) connecting units **II** and **III** (Figure 4). Based on the chemical shift of C(16) at δ_C 92.3 ppm in DMSO-d_6 it was connected to the two remaining carbons, C(15) and C(17) and not the oxygen. To keep the constraints of the suggested molecular formula, C(17) was connected to C(13) *via* a lactone. This cyclization is in agreement with the downfield shift of the oxymethine H(13).

Assignment of C(17) and the δ-lactone in compounds 2-4

Based on the molecular formula suggested by high-resolution mass spectrometry (Figure S38), only one carbonyl group remained unassigned for all compounds (see discussion above). A structure with an open ring containing a hydroxy acid would be 18 Da heavier and is therefore unlikely. Based on the missing bonding partners, the carbonyl group on C(17) had to be placed next to the quaternary carbon C(16). Furthermore, it must be connected intramolecularly to an additional oxygen. In all compounds formation of a six-membered ring is possible, in compound **4** also a five membered ring could be formed. There are several arguments for a six-membered rings in compounds **2**-**4**: i) Compared to a oxymethine with a free hydroxy group the chemical shifts of H(13) and C(13) are more downfield in **2**-**4** and fit the shifts reported for janustatin A, which contains a six-membered ring (Table S5). ii) For **4**, the chemical shifts of H(14) and C(14) fit a oxymethine with a free hydroxy group. In the rearranged five-membered ring harboring janustatin A', these shifts are even more downfield due to deshielding effect from the carbonyl at C(17) and the double bond C(15)/C(16). iii) The coupling $^3J_{H13H14}$ = 11.2 Hz suggests both hydrogens to be part of a cyclic system.

Bioactivity tests of isolated compounds (1-4) against HeLa cells

HeLa cells purchased from ATCC were cultivated at 37 °C, 5% (v/v) CO_2 for 3-4 days. Cells were washed with PBS buffer (Sigma D8537), 0.05 % trypsin-EDTA solution (Thermo 25300-054) was added and the plate was incubated for 5 min at 37 °C. The cells were resuspended in 5 mL medium (DMEM-GlutaMAX) supplemented with 10% FCS (Eurobio CVFSVF00-01), and 50 µg/mL penicillin-streptomycin (Corning). After counting the cells under a ZEISS Axiovert 25 microscope using a Neubauer hemocytometer a 17,000 cells/mL suspension was prepared and 200 µL were transferred into each well of three 96-well plates. After one day of cultivation, 2 µL of compounds (**1**-**4**), DMSO as a negative control and doxorubicin (1 mg/mL) were added to row B of the plate and a 5-fold serial dilution was performed to row G. After four days of cultivation, 50 µL of 3-(4,5-dimethylthiazol-2-yl)-2,5-diphenyltetrazolium bromide (MTT, 1 mg/mL in sterile H_2O) were added and the cells incubated for 3 h at 37 °C. The supernatant was discarded and then 150 µL of DMSO were added to the wells. The absorbance at 570 nm was measured on a spectraMAXplus spectrometer (Molecular Devices LLC). Results are shown in Figure S40.

```
query        1530 QLVAAVQTAFKELP.[1].GGVSYDWL.[ 9].P.[5].PLRINYLGHTD.[ 1].LDS.[ 2].FE.[2].WDT      1591
AAB96628     1591 RTVREIKECLRTLP.[1].QGLGYGIL.[16].P     HFGFNYLGRVS.[16].GLG.[18].WV.[2].SGP.[2]. 1687 Streptomyces filam...
BAB74348     1421 ENLKYVKERLREIP.[1].KGISYGLL.[18].S     AISFNYLGQLD.[ 3].SQG.[ 2].LG.[2].KES      1488 Nostoc sp. PCC 7120
P39846       2406 HLIKQTKDMLHRIP.[1].KGAGYGVL.[13].P     EISFNYLGQFD.[ 2].IQS.[ 2].FE.[2].DIK      2467 Bacillus subtilis ...
AAF08796     2667 HYIKTAKEELRQIP.[1].KGIGYGVL.[15].P     EISFNYLGQFD.[ 2].IQN.[ 2].VQ.[2].SYS      2730 Bacillus subtilis ...
P27206       3432 YQLKQMKEDIRHVP.[1].KGVGYGIL.[17].P     DVSFNYLGQFD.[ 2].SDA.[ 2].FT.[2].ELP      3497 Bacillus subtilis ...
P09095        920 DYIKLTKENMRKIP.[1].KGIGYDIL.[17].P     EVTFNYLGQFD.[ 2].MRT.[ 2].FT.[2].PYS      985  Brevibacillus para...
Q04747       3427 AVIKTVKENLRRIP.[1].KGVGYGIL.[13].P     EISFNYLGQFD.[ 2].VKT.[ 2].FE.[2].AFD      3488 Bacillus subtilis ...
P39845       2411 YRIKTTKDMLRRVP.[1].KGTGYGLL.[10].P     EVSFNYLGQFS.[ 2].KEV.[ 2].FQ.[2].YYQ      2469 Bacillus subtilis ...
P39847       2398 TSVKTVKDTLGRIP.[1].KGVGYGML.[16].P     EISFNYLGQFN.[ 2].ERQ.[ 2].FR.[2].SLG      2462 Bacillus subtilis ...
O68006       4050 QNIKMVKEALRKIP.[1].KGIGYGVL.[13].A     PLSFNYFGEMN.[ 2].MNR.[ 2].FS.[2].PFS      4111 Bacillus lichenifo...
O68008       4931 RTIKMVKEHLRNVP.[1].KGIGYGIL.[14].S     PILFNYLGQLD.[ 2].INS.[ 2].FS.[2].HLS      4993 Bacillus lichenifo...
AAC32048     1879 AAAKAVKEQLRAVP.[1].RGLGYGVL.[11].P     QILFNYLGRFT.[ 3].GND      WR.[2].AGI.[2]. 1939 Mycobacterium smeg...
CAB53322     1038 AALKAVKEDLARVP.[1].HGVSYGAL.[12].P     QVLFNYLGRFD.[ 3].SGD      WQ.[2].SVN.[2]. 1099 Streptomyces coeli...
CAA11795     2857 RLLKQVKENVRAVP.[1].GGLGYGML.[16].A     EIGFNYLGRFS.[ 3].DGE.[ 3].WQ     IKG.[1]. 2922 Amycolatopsis orie...
CAB55600     4359 GIVKAVKEQLRSLP      EGLTYGLL.[13].P     TIGFNYLGRLG.[ 4].ELS.[ 3].WR.[2].QDS.[2]. 4424 Mycobacterium smeg...
EQL42983      182 ESLKAIKEQLRGVP.[1].KGVGYGLL.[17].P     RITFNYLGRFD.[ 3].DGA.[ 2].LV.[2].TES      248  Pseudomonas aerugi...
WP_010973248 2410 RLLLDVKDTLRRIP.[1].NGVGFGVL.[14].P     QLRFNYLGQMD.[ 3].AID.[ 2].FA.[2].GIT      2473 Agrobacterium fabrum

query        1592 AN.[1].RFIP.[1].Q.[1].KRTT.[1].IEVLMFFRN     KQ     LHLDIDYS.[1].QRYRDETIQELAEGY 1641
AAB96628     1688 VG.[1].GQDP.[1].L.[1].VAHA     VEFNAITLD.[3].GP.[1].LSVTWSWP.[1].TLLSESRIRELARFW 1740 Streptomyces filam...
BAB74348     1489 TG.[2].SHPL.[1].Q      RRYV     LNINAWIAQ     SQ     LQIQWRYS.[1].NLHDTTTIENLAQQF 1537 Nostoc sp. PCC 7120
P39846       2468 PG.[1].EISP.[1].W.[1].RPYA     LDISGAVSS     GC     LNMHIIYN.[1].FQFEEKTIQTFSRHF 2516 Bacillus subtilis ...
AAF08796     2731 CG.[1].DSSG.[1].Q.[1].RPYV     LNINGMIVN     DR     LMVTISYS.[1].KQYAKETIDQLSAII 2779 Bacillus subtilis ...
P27206       3498 SG.[1].SLSP.[1].T.[1].KPNA     LDVVGYIEN     GK     LTMSLAYH.[1].LEFHEKTVQTFSDSF 3546 Bacillus subtilis ...
P09095        986 GG.[9].NLSP.[1].S.[1].VYTA     LNITGLIEG     GE     LVLTFSYS.[1].EQYREESIQQLSQSY 1042 Brevibacillus para...
Q04747       3489 MG.[1].QVSG.[1].S.[1].ALYA     LSFSGMIRN     GR     FVLSCSYN.[1].KEFERATVEEQMERF 3537 Bacillus subtilis ...
P39845       2470 PR.[1].EIAG.[1].R.[1].REYE     LDINALITD     GR     LHVKAVYT     QVFSKHSIECFMDRF 2517 Bacillus subtilis ...
P39847       2463 SG.[1].DITH.[1].W.[1].REQI     IEMSAMAAD     KK     LHFNLSYP.[1].ARFHRNTMEQLINRI 2511 Bacillus subtilis ...
O68006       4112 PG.[1].SIGG.[1].I.[1].RHCA     IEMNAISLN     GE     LTIYTTFN.[1].DQYQTSTIEQLNQSF 4160 Bacillus lichenifo...
O68008       4994 PG.[1].AAGK.[1].I.[1].REHP     LEINAVVFR     GK     LAIQTTYN.[1].RAYSEDVVRAFAQNY 5042 Bacillus lichenifo...
AAC32048     1940 LT.[1].GVDP      S.[1].PAMS     LEINALAEE.[3].GT.[1].LSMTLAWP.[1].GLLDADDVSELGSMW 1991 Mycobacterium smeg...
CAB53322     1100 LG.[1].QRDP.[1].M.[1].LPRA     LEFNAIAEP.[3].GA.[2].LVTAISWP.[1].GMFTDTDITTLGAYY 1153 Streptomyces coeli...
CAA11795     2923 VG.[1].AAEQ.[1].T.[1].LRHV     VEIDAVVLD.[3].GP.[1].FTLTVTWA.[1].RIVGDAEAESFANAW 2975 Amycolatopsis orie...
CAB55600     4425 LS.[2].ASAV.[1].M.[1].LMHT     VELNAGTID.[3].GP.[1].LRASWTWA.[1].SAFDEEQATRLSRLW 4478 Mycobacterium smeg...
EQL42983      249 AG.[1].AQDP.[1].A.[1].LANW     LSIEGQVYG     GE     LSLHWSFS.[1].EMFAEATVQRLVDDY 297  Pseudomonas aerugi...
WP_010973248 2474 SG.[1].MYGA.[1].N.[1].RDTI     LEINAMVVR     GE     LQLQWVYG.[1].QLHSEDTIRTLADHF 2522 Agrobacterium fabrum

query        1642 F      ALIR 1646
AAB96628     1741 D.[4].GLVA 1749 Streptomyces filamentosus
BAB74348     1538 I.[4].AIIQ 1546 Nostoc sp. PCC 7120
P39846       2517 K.[4].NIIE 2525 Bacillus subtilis subsp. subtilis str. 168
AAF08796     2780 Q.[4].TVIE 2788 Bacillus subtilis subsp. spizizenii ATCC 6633
P27206       3547 K.[4].RIIE 3555 Bacillus subtilis subsp. subtilis str. 168
P09095       1043 Q.[4].AIIA 1051 Brevibacillus parabrevis
Q04747       3538 K.[4].MLIR 3546 Bacillus subtilis subsp. subtilis str. 168
P39845       2518 H.[4].ETIE 2526 Bacillus subtilis subsp. subtilis str. 168
P39847       2512 E.[4].DIMK 2520 Bacillus subtilis subsp. subtilis str. 168
O68006       4161 K.[4].KIVD 4169 Bacillus licheniformis
O68008       5043 K.[4].AVIR 5051 Bacillus licheniformis
AAC32048     1992 A.[4].ALTR 2000 Mycobacterium smegmatis
CAB53322     1154 V.[4].GLAA 1162 Streptomyces coelicolor A3(2)
CAA11795     2976 L.[4].GLAA 2984 Amycolatopsis orientalis
CAB55600     4479 F.[4].GICA 4487 Mycobacterium smegmatis str. MC2 155
EQL42983      298 A.[4].ALIE 306  Pseudomonas aeruginosa VRFPA03
WP_010973248 2523 R.[4].TLIQ 2531 Agrobacterium fabrum
```

Figure S1. Alignments of 18 NRPS-para261 domains (TIGR01720).

The conserved motifs (e.g. 'RxxPxxGxxYGxL' and 'FNYLGxxD') are highlighted in red. The P domain (query from JanE) is also present in characterized NRPSs such as MycB from *Bacillus subtilis* from mycosubtilin biosynthesis.[7]
https://www.ncbi.nlm.nih.gov/Structure/cdd/cddsrv.cgi?ascbin=8&maxaln=10&seltype=2&uid=TIGR01720

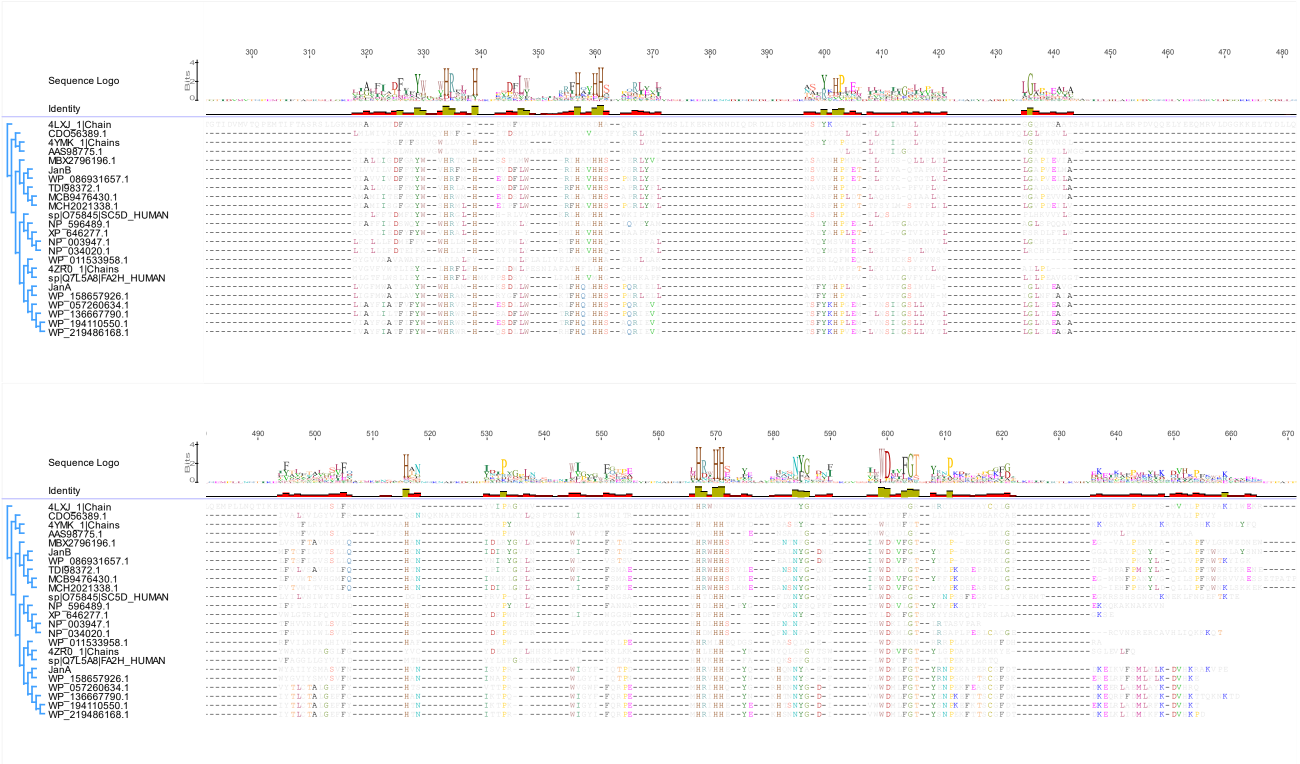

Figure S2. Protein alignment of JanA and JanB with characterized desaturases and hydroxylases.
The characteristic active-site histidine residues are conserved in the two maturation enzymes in janustatin biosynthesis in the bacterium *G. sunshinyii*. Histidine residues in the putative active site are conserved. The alignment and visualization was performed in Geneious version 7.1.9 (Biomatters) using the MUSCLE[8] algorithm.

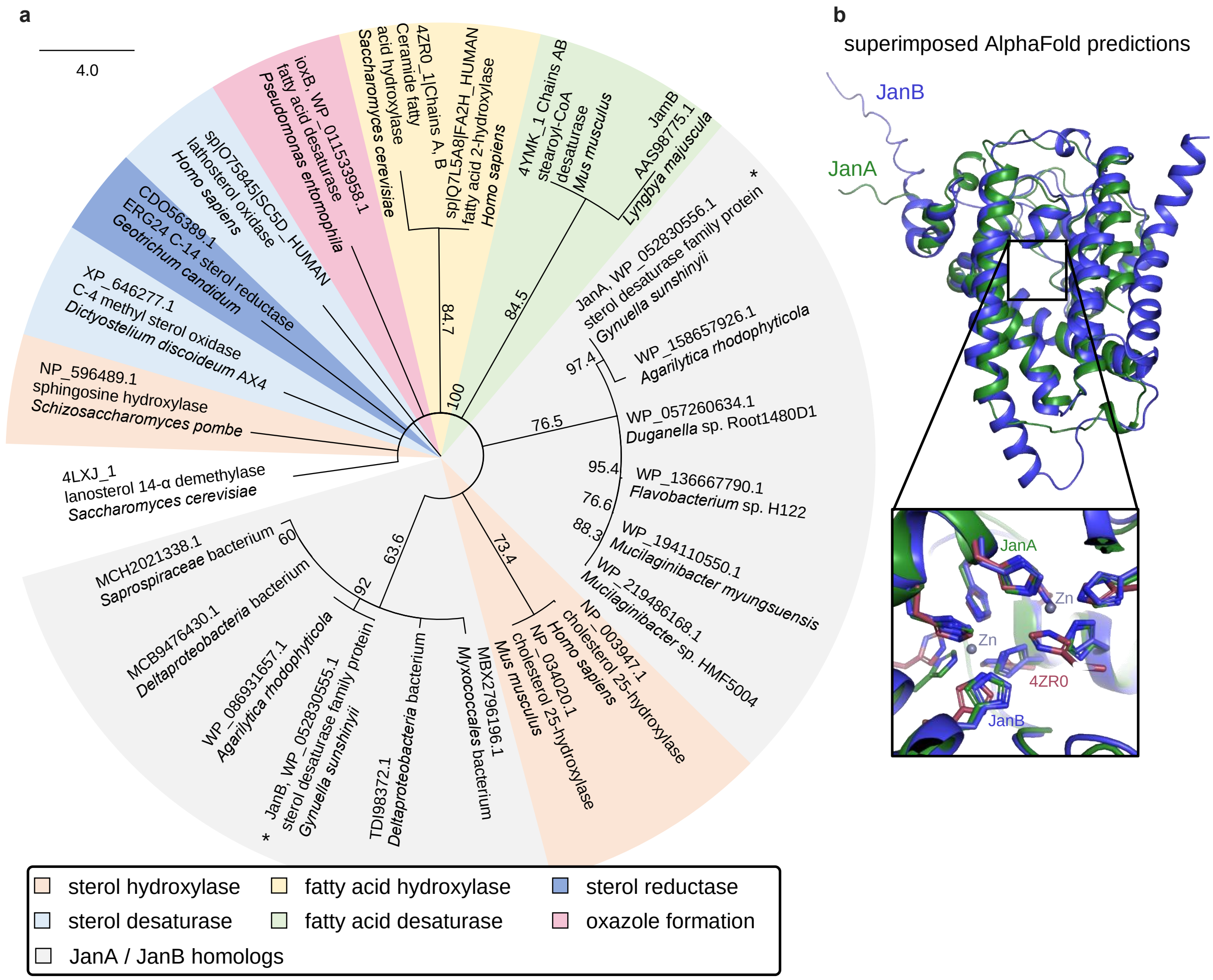

Figure S3. Phylogenetic analysis of JanA and JanB.

A, In a phylogenetic tree of selected hydroxylase/desaturase enzymes, JanA and JanB (*) form distinct clades. The phylogenetic tree was constructed in Geneious version 7.1.9 (Jukes-Cantor, Neighbor-Joining, 1000 Bootstrap, of a MUSCLE alignment; Biomatters, Figure S2), demethylase (PDB: 4LXJ_1) was used as an outgroup. Enzymes were classified based on the primary publications and manually colored. **B**, Despite displaying only 29% sequence identity, the predicted AlphaFold structures of JanA (green) and JanB (blue) align very well (RMSD = 1.22, https://alphafold.ebi.ac.uk/ structures: A0A0C5VTG4_9GAMM, A0A0C5W4C1_9GAMM)). Inset: In the predicted structures ten histidine residues face into a pocket. In characterized desaturases and hydroxylases, these conserved residues bind metal ions. The histidine residues and Zn-ions from the sphingolipid α-hydroxylase (red, Scs7p, PDB: 4ZR0) were superimposed onto the predicted structures.

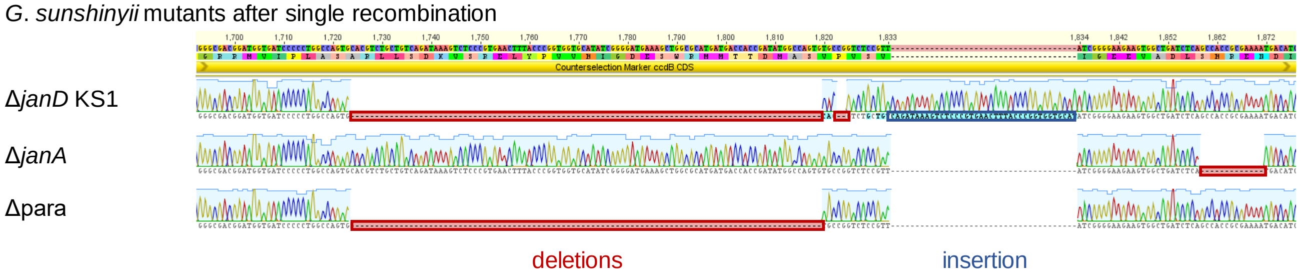

Figure S4. Sequencing of single cross-over mutants shows deletions and insertions in the counterselection marker *ccdB*. Suicide plasmids (pSW8197) with homology arms targeting different locations in the *G sunshinyii* genome (the first KS encoded by *janD*[9], *janA*, and the NRPS-para 261 domain-encoding region in *janE*; Table S1) were transferred to the acceptor strain by bacterial conjugation. Kanamycin selection of single cross-over mutants followed by PCR amplification of the counter-selection marker gene *ccdB* revealed large deletions and insertions. These mutations caused problems during the counter-selection. We therefore changed to another plasmid system (pEB17).

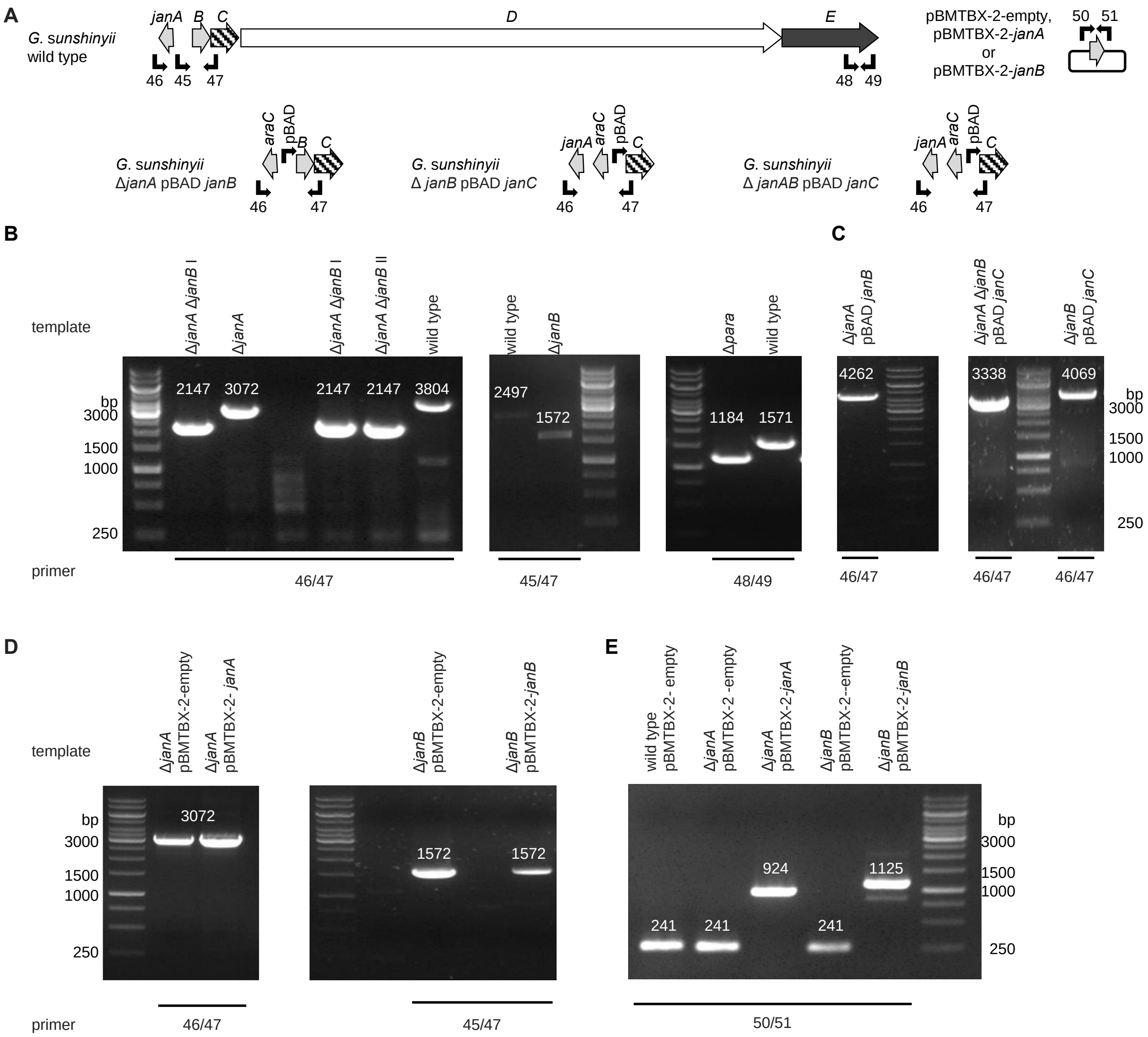

Figure S5. PCR-based genotyping of mutants used for the *janA* and *janB* complementation experiment.
A, Map of the janustatin BGC and the pBMTBX-2 plasmid detailing the regions where primers bind (not to scale, primer sequences: Table S1). **B**, The differences of 732 bp, 925 bp, 1657 bp, and 387 bp confirm deletions of *janA*, *janB*, both *janA* and *janB*, and the *para*-domain, respectively. PCR bands were purified and sequenced. **C**, PCRs confirming the insertion of *araC* plus the pBAD promoter (1190 bp) in the Δ*janA* pBAD *janB*, Δ*janB* pBAD *janC*, and Δ*janAB* pBAD *janC* gene activation strains. **D**, The 3072 bp PCR product confirms deletion of *janA*, the 1573 bp PCR product that of *janB* in the complementation mutants. **E**, The 924 bp and the 241 bp PCR product confirm the presence of pBMTBX-2 harboring *janA* or no gene under pBAD control in the *janA* deletion mutant. The 1125 bp and the 241 bp PCR product confirm the presence of pBMTBX-2 harbouring *janB* or no gene under pBAD control in the *janB* deletion mutant. Expected PCR product sizes (in bp) are shown in white. Ladder: ThermoFischer GeneRuler 1 kb.

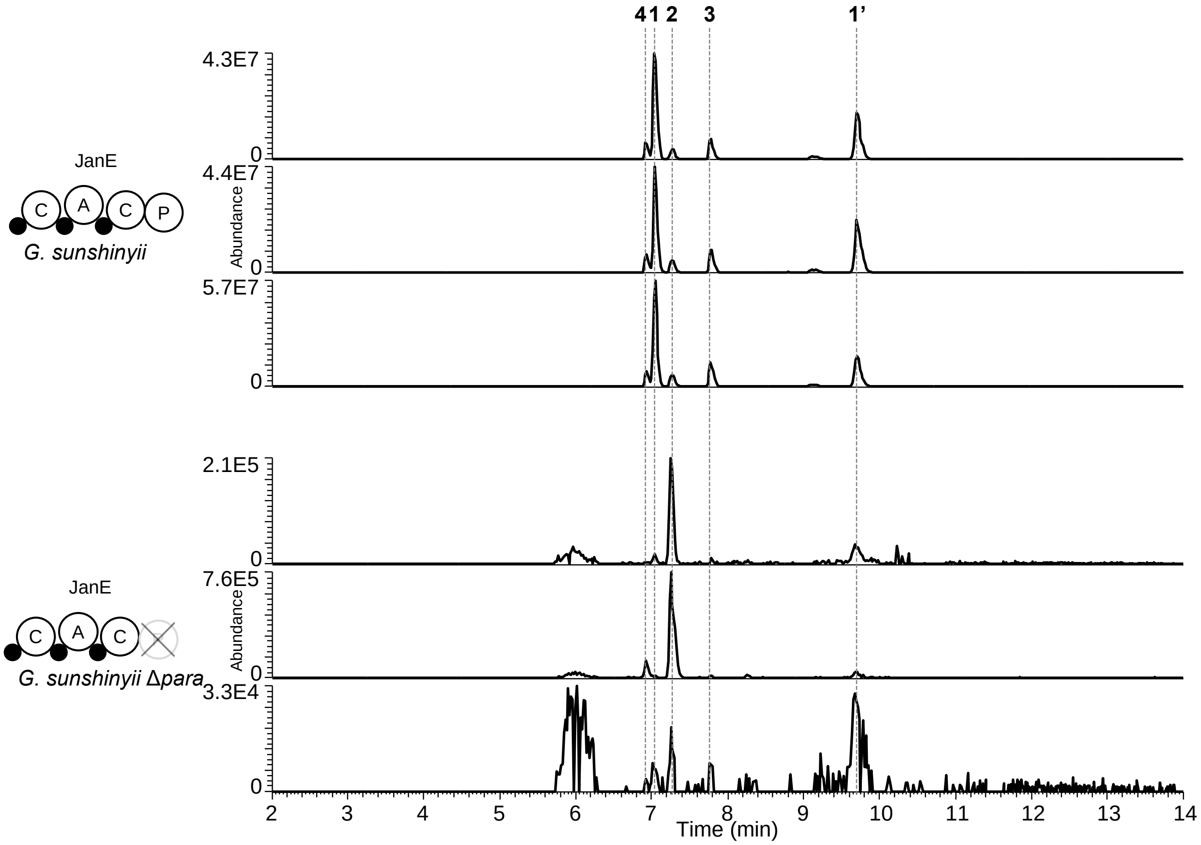

Figure S6. LC-MS analysis of *G. sunshinyii* wild type and the Δ*janE* NRPS-para261 deletion mutant extracts. The domain organization of the NRPS, JanE, in the wild type *G. sunshinyii* and a P domain deletion mutant and the corresponding LC-MS profiles of organic extracts are shown. Plots are extracted ion chromatograms (*m/z* 432.2744, 434.2901, 448.2694, 450.2850) of extracts in biological triplicates. For quantification see Table S3.

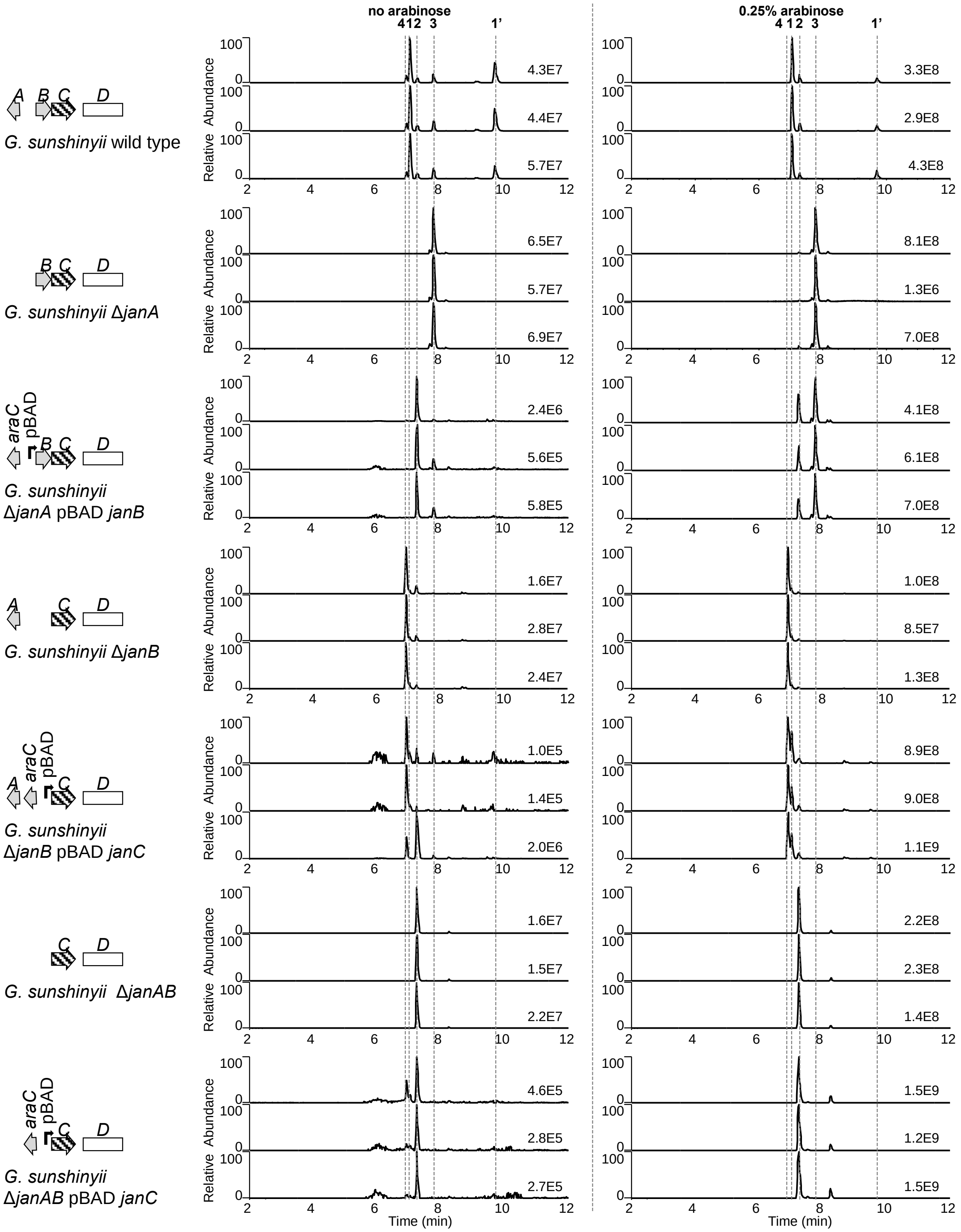

Figure S7. Genetic engineering enables arabinose induced control over biosynthetic intermediate (2-4) titers.
The *jan* BGC organization (compare Figure 1) of *G. sunshinyii* mutants with the *araC*-pBAD promoter system integrated into the genome and the corresponding LC-MS profiles of uninduced and induced organic extracts are shown. Plots are extracted ion chromatograms (*m/z* 432.2744, 434.2901, 448.2694, 450.2850). Cultures were either uninduced or induced with 0.25% arabinose in ½ MB medium. For quantification see Table S7.

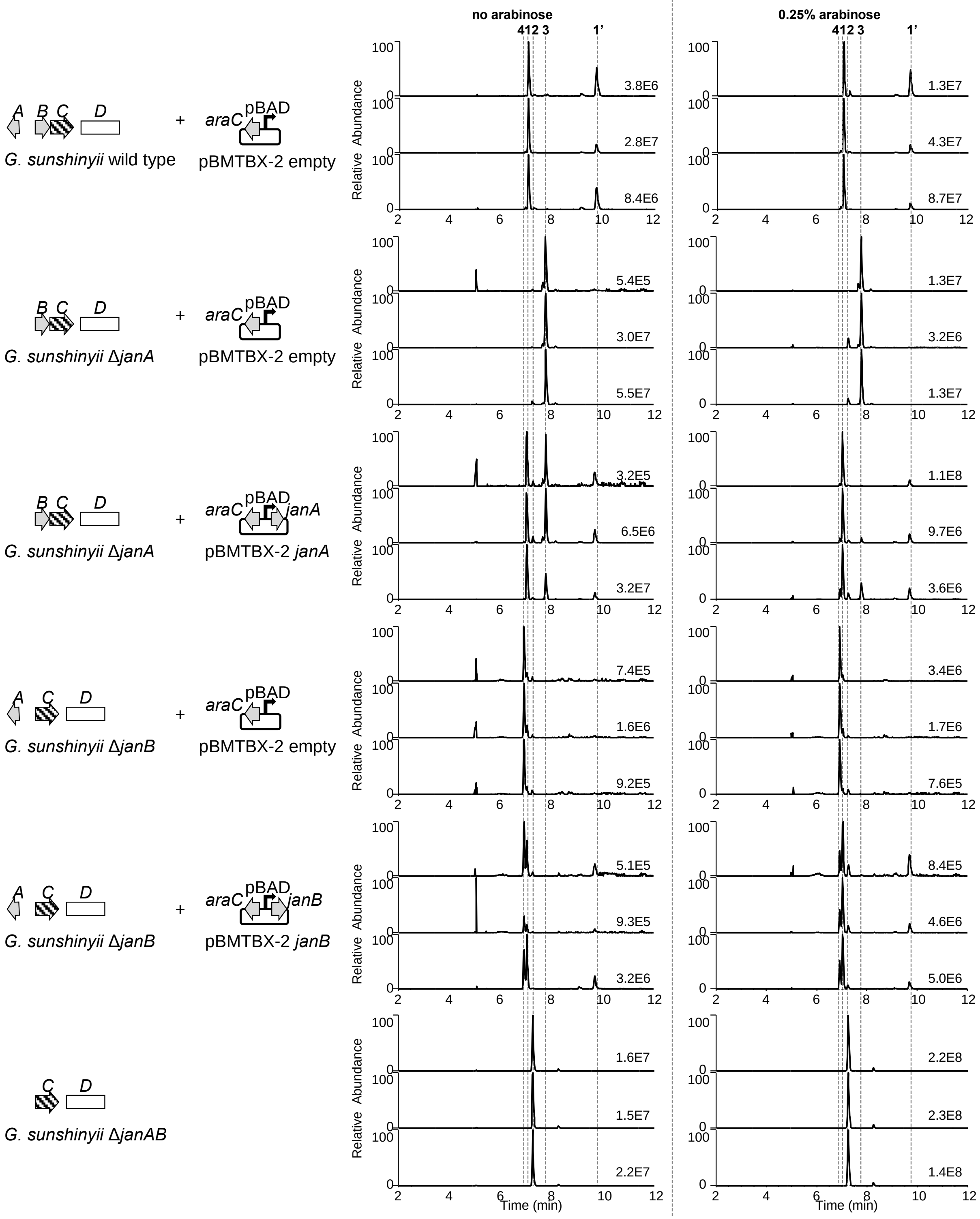

Figure S8. LC- MS profiles of deletion and complementation experiments.

The *jan* BGC organization (compare Figure 1) of *G. sunshinyii* mutants complemented with the deleted genes on plasmids and the corresponding LC-MS profiles of organic extracts are shown. Plots are extracted ion chromatograms (*m/z* 432.2744, 434.2901, 448.2694, 450.2850). Cultures were either uninduced or induced with 0.25% arabinose in ½ MB medium. Strains with plasmids were cultivated with 50 µg/mL kanamycin. Plots are extracted ion chromatograms (*m/z* 432.2744, 434.2901, 448.2694, 450.2850). For quantification see Table S8.

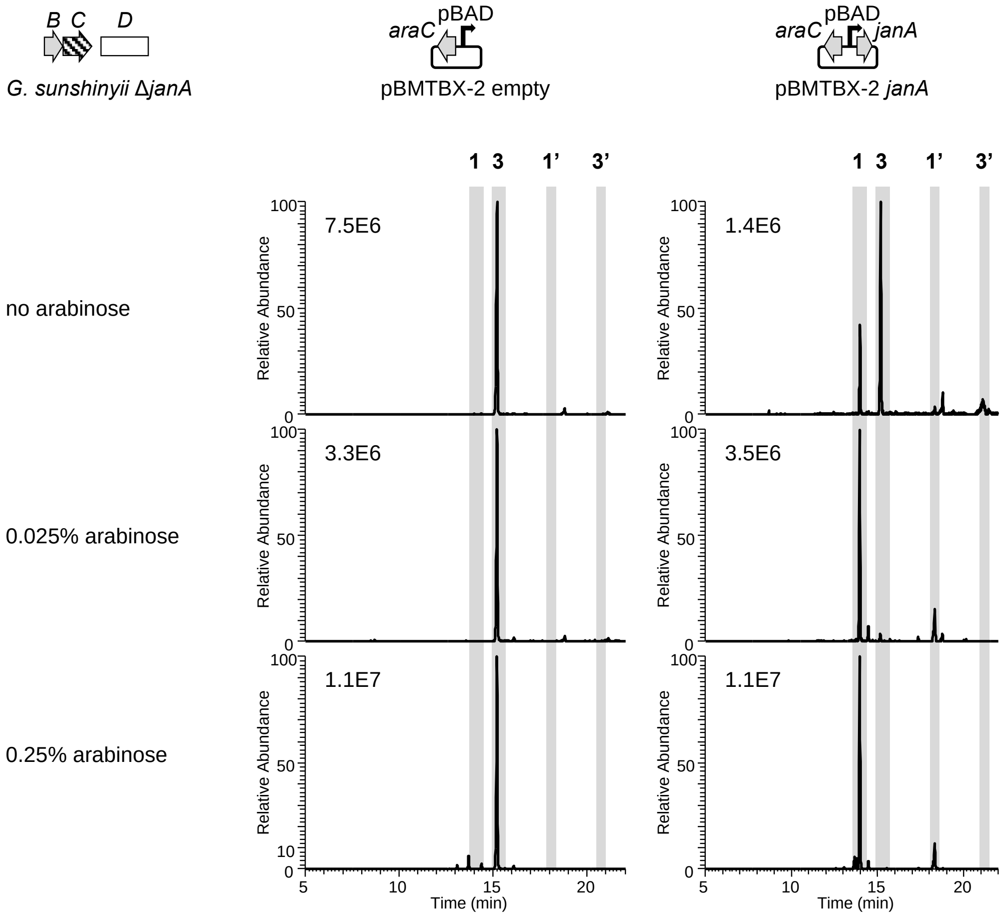

Figure S9. Optimization of pBAD induction in *G. sunshinyii*.
The *janA* deletion mutant carrying pBMTBX-2 with or without *janA* was cultivated without arabinose, with 0.025% and 0.25% (w/v) arabinose. Production of **1** is only observed in the strain containing pBMTBX-2 *janA*. Some background activity of JanA can be observed without addition of arabinose, with 0.025% arabinose most of janustatin E (**3**) is converted to **1**. With 0.25% arabinose, **3** cannot be detected anymore. The same is true for the intermediates of janustatin B (**1'**). Based on these results we used 0.25% arabinose induction for other experiments.

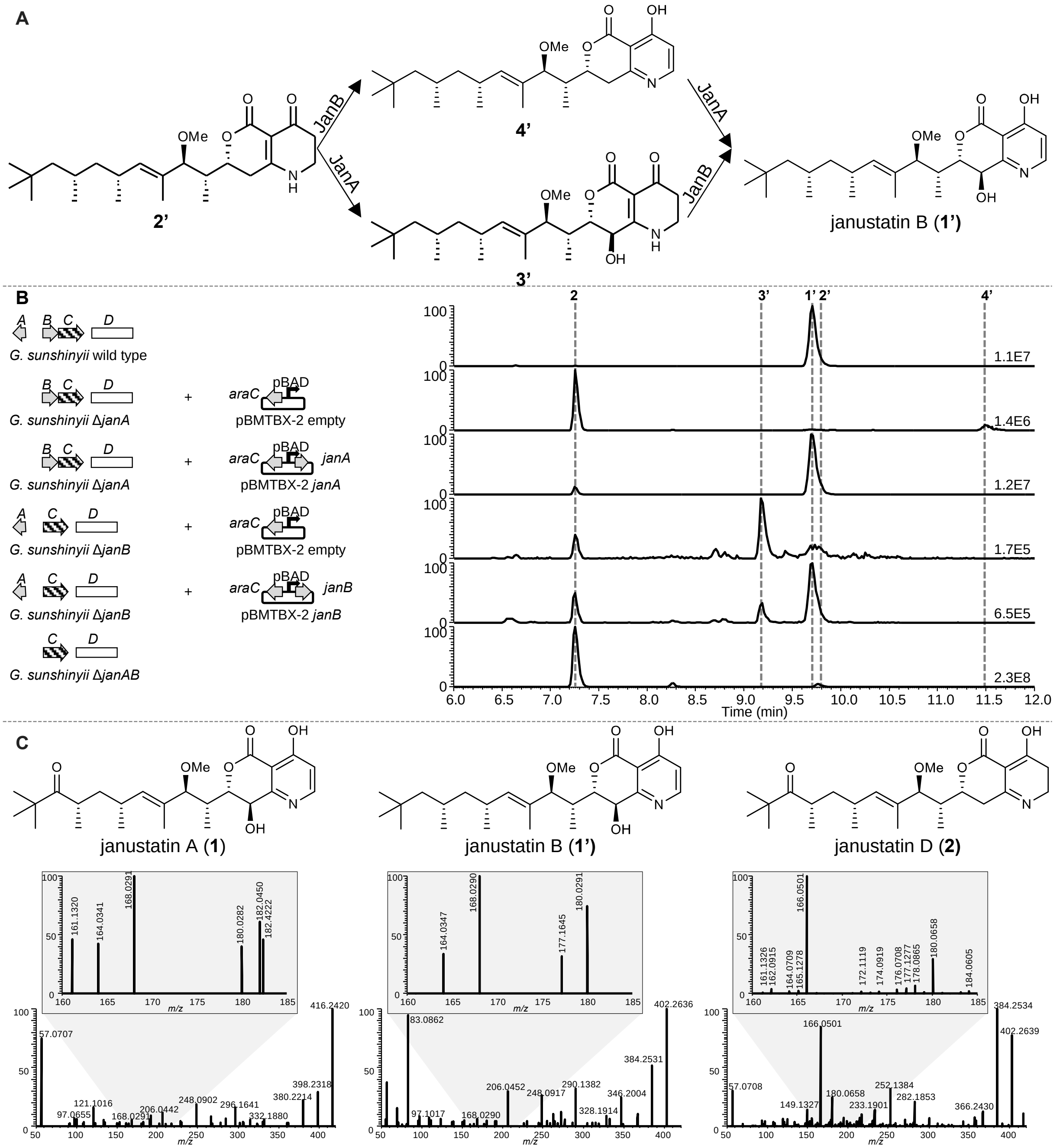

Figure S10. LC-MS analysis of deletion/complementation experiments of *janA* and *janB* reveals ions corresponding to janustatin B intermediates (1'-4').

A, Biosynthetic scheme of janustatin B (**1'**) maturation. **B**, Complementation of *janA* or *janB* in mutant strains recovers the wildtype phenotype, production of **1'** (EICs *m/z* 418.2952, 420.3108, 434.2901, 436.3057). **C**, MS/MS spectra of janustatin A (**1**) and janustatin B (**1'**) from the wildtype culture compared to isolated janustatin D (**2**). The fragment ions of **1'** are different from those of the isobaric compound **2**, and similar to the fragments of **1**. In particular, ion *m*/*z* 164.0347 with the suggested formula $C_8H_6O_3N$ Δ 0.43 mmu is characteristic for the aromatic bicyclic system, that is present in **1'** while in **2** the reduced ion *m*/*z* 166.0501 with the suggested formula $C_8H_8O_3N$ Δ 0.24 mmu was observed (compare Figure S38).

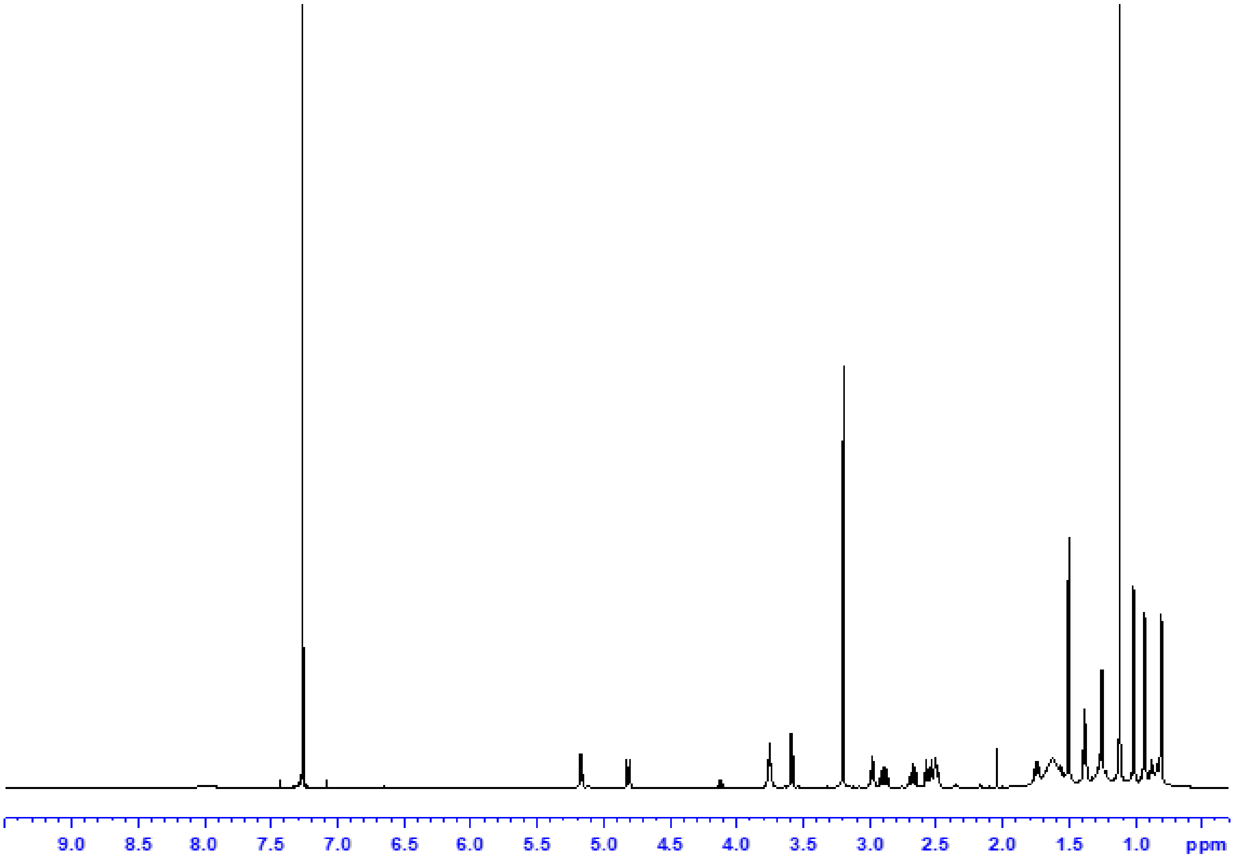

Figure S11. **^{1}H NMR spectrum of janustatin D (2) in $CDCl_3$ at 298 K (600 MHz).**

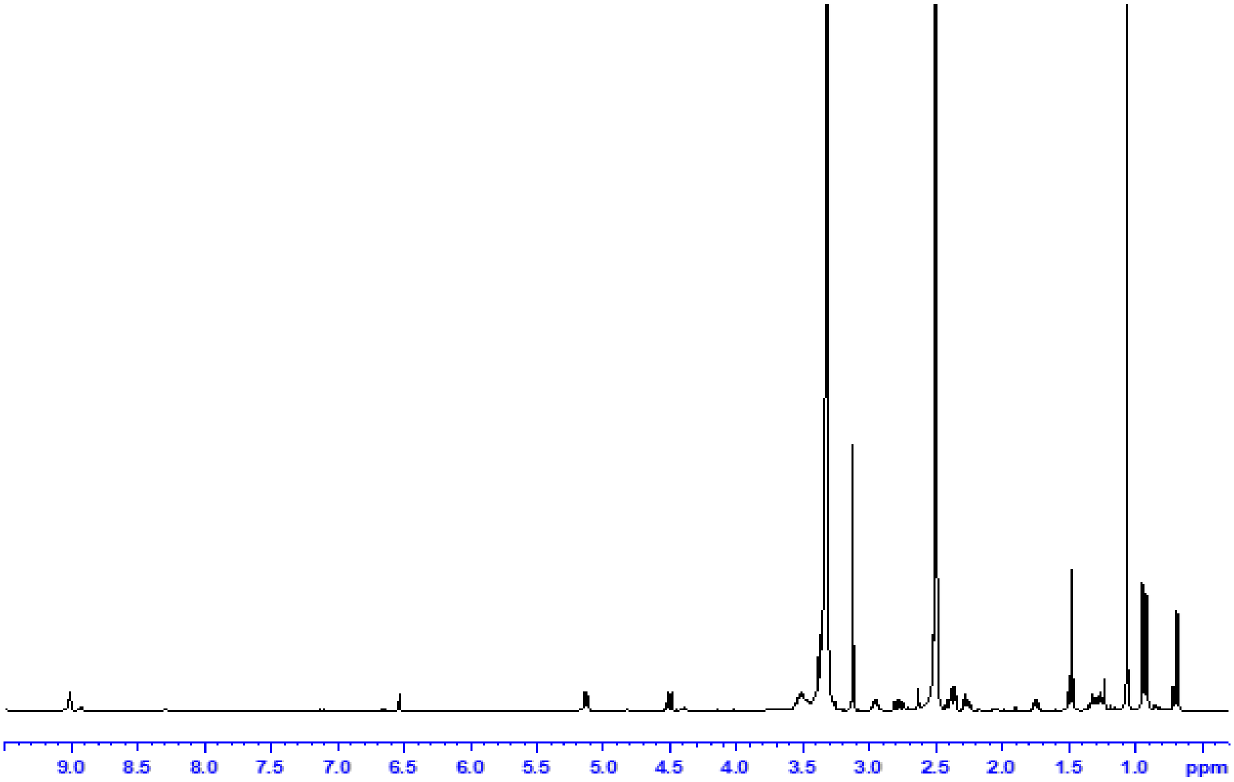

Figure S12. **^{1}H NMR spectrum of janustatin D (2) in DMSO-d_6 at 298 K (500 MHz).**

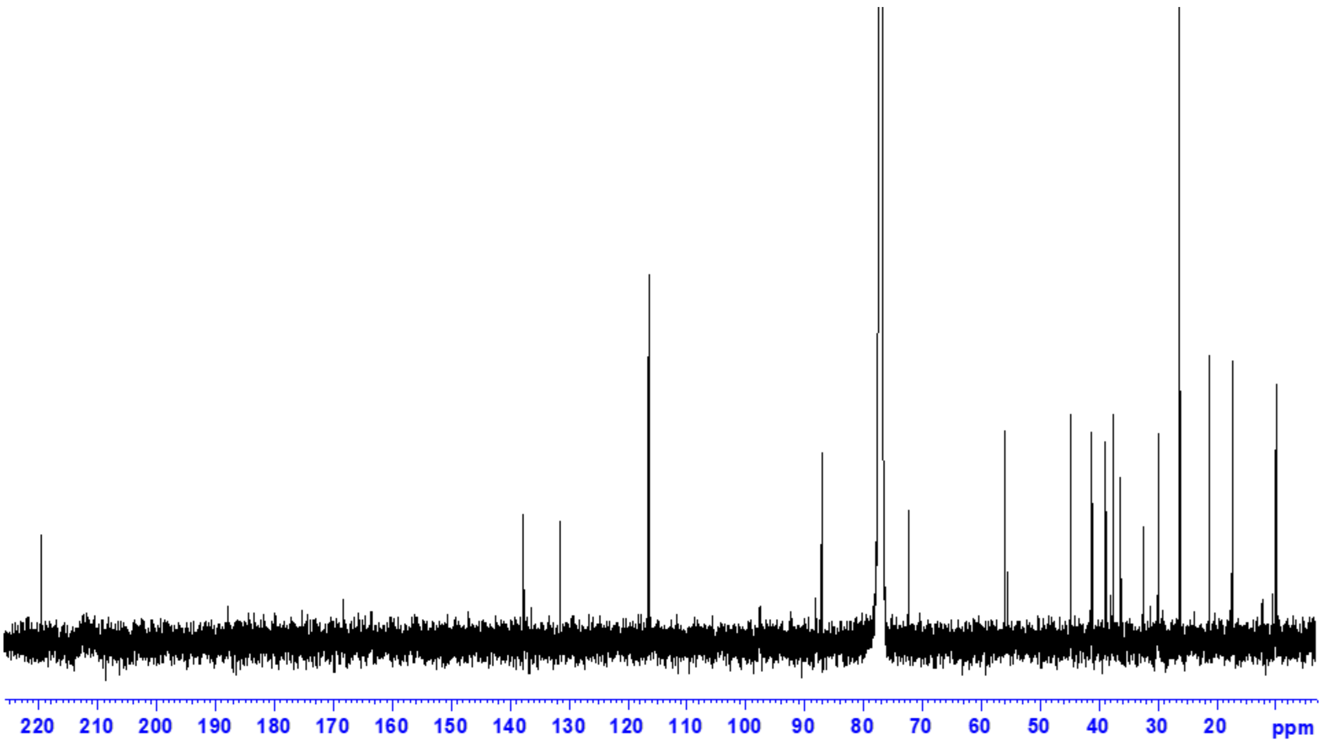

Figure S13. [1]**C NMR spectrum of janustatin D (2) in $CDCl_3$ at 298 K (150 MHz).**

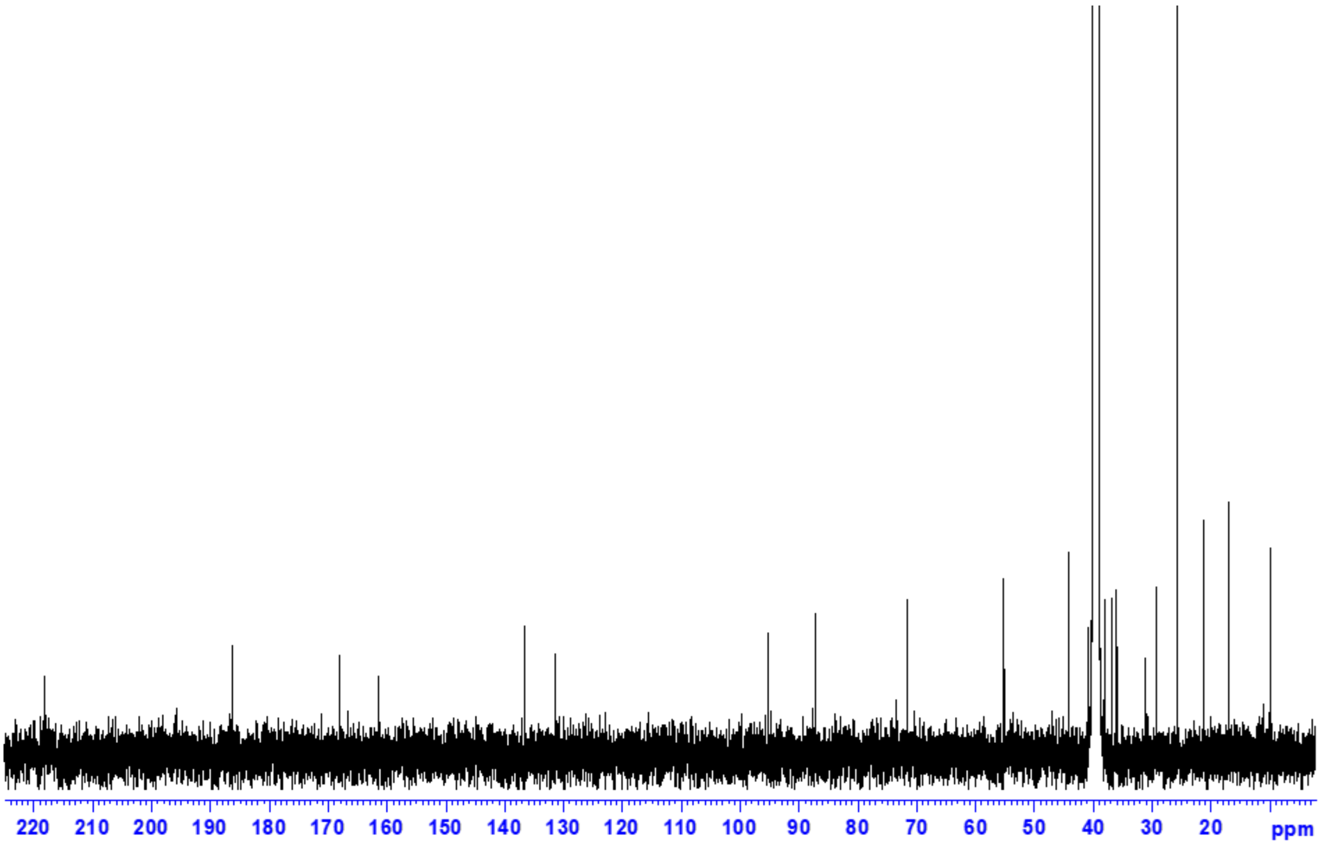

Figure S14. [1]**C NMR spectrum of janustatin D (2) in DMSO-*d_6* at 298 K (150 MHz).**

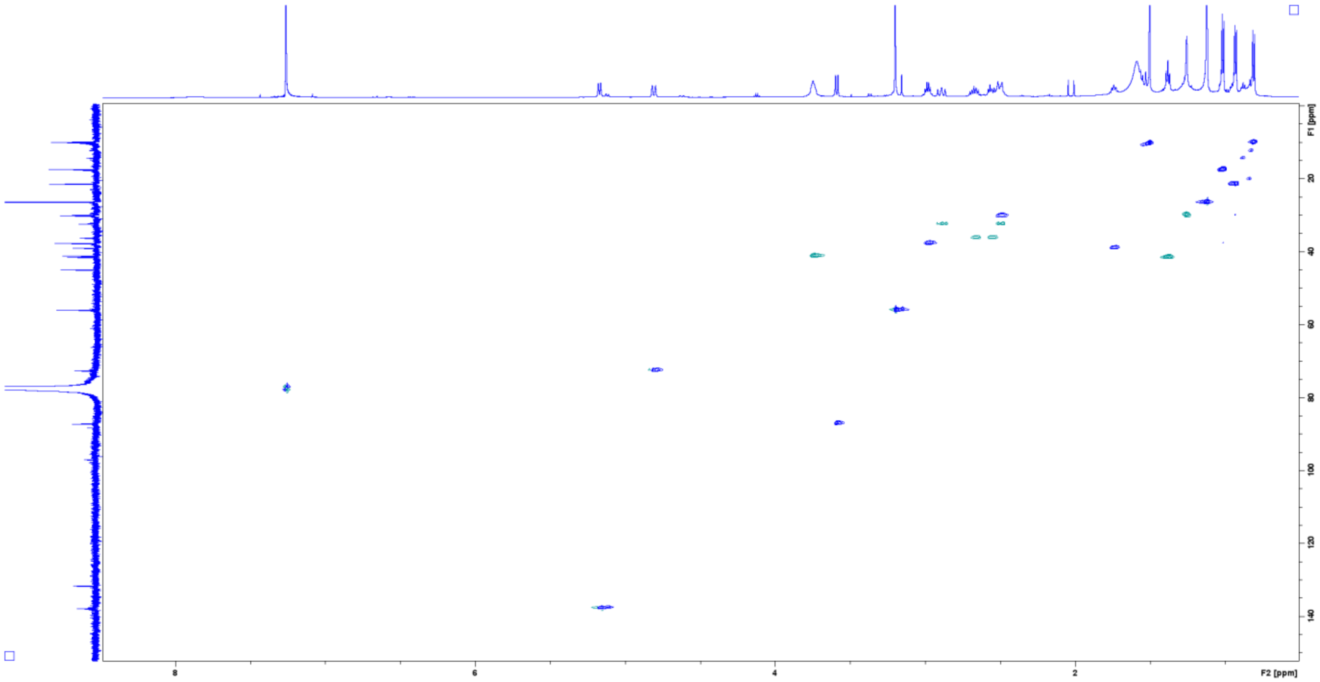

Figure S15. HSQC spectrum of janustatin D (2) in $CDCl_3$ at 298 K.

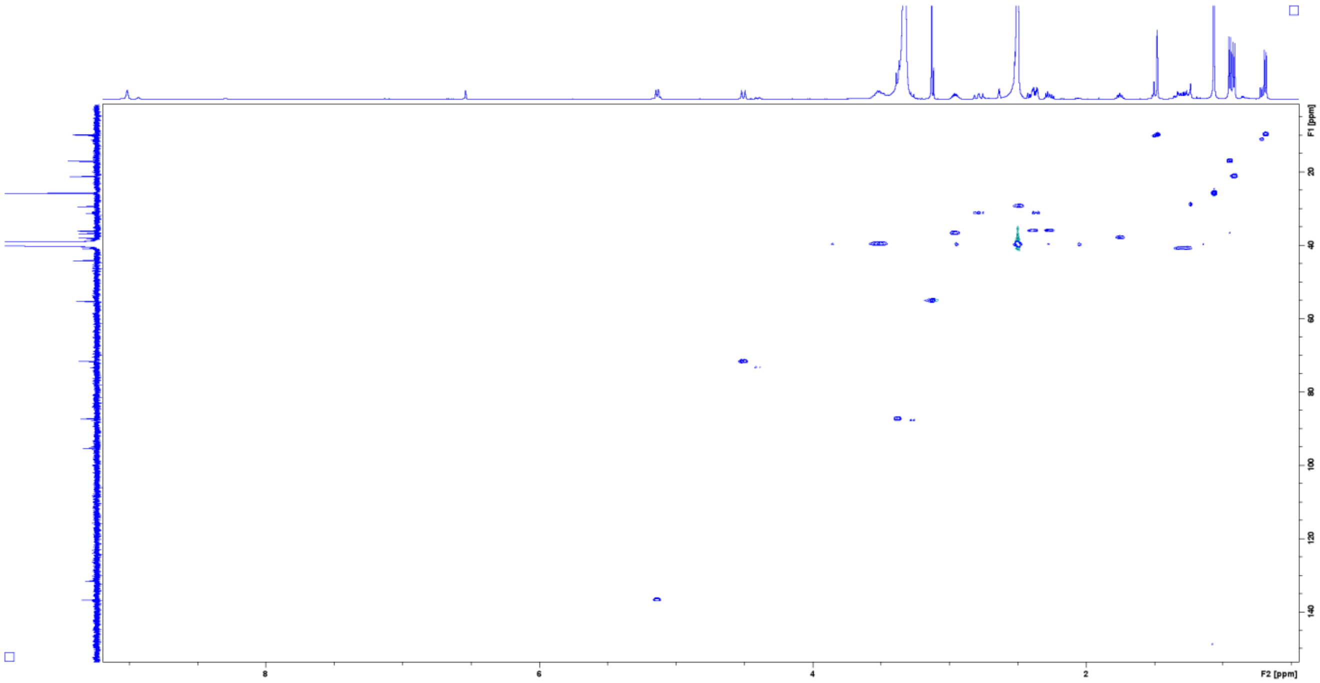

Figure S16. HSQC spectrum of janustatin D (2) in DMSO-d_6 at 298 K.

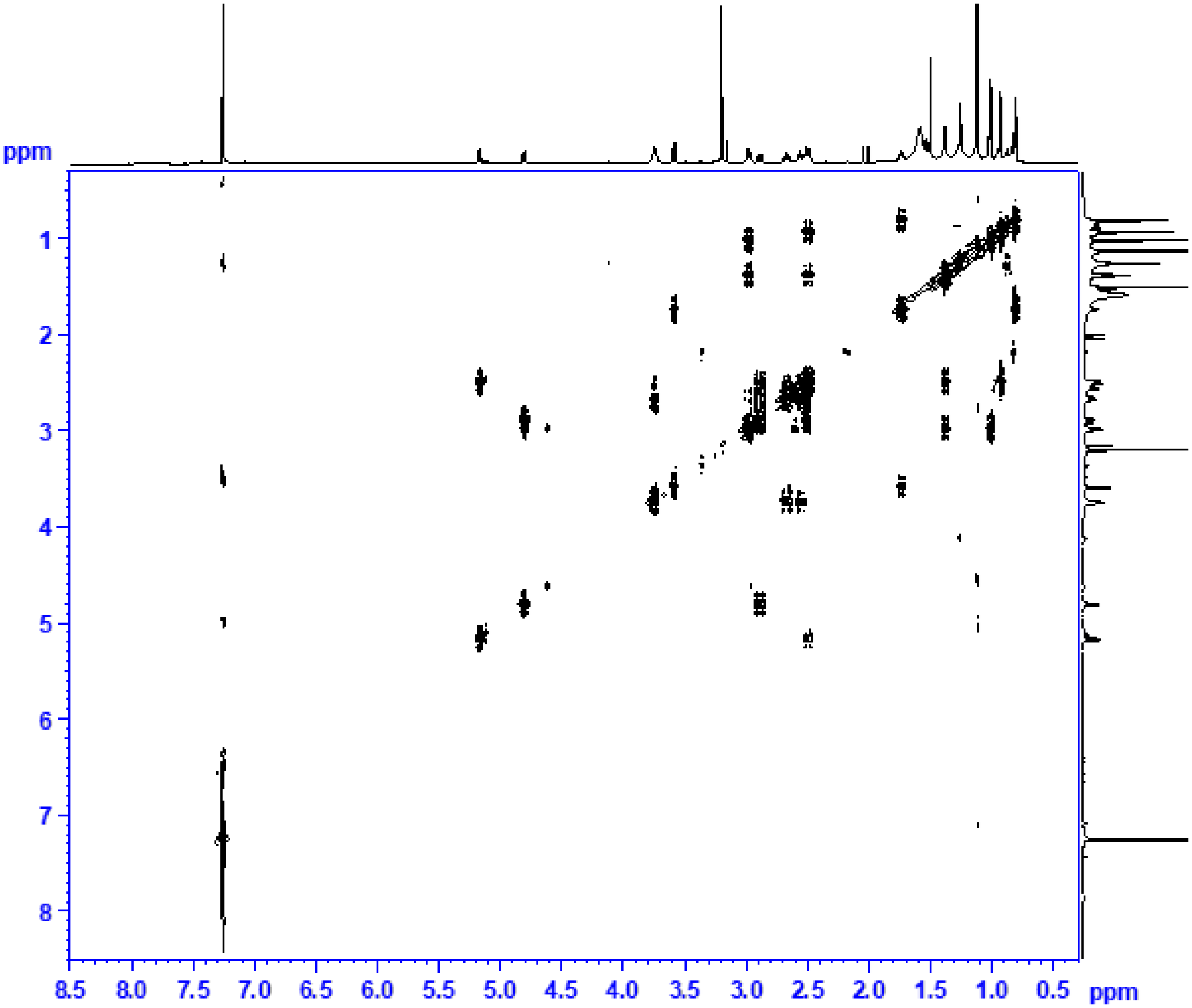

Figure S17. COSY spectrum of janustatin D (2) in $CDCl_3$ at 298 K.

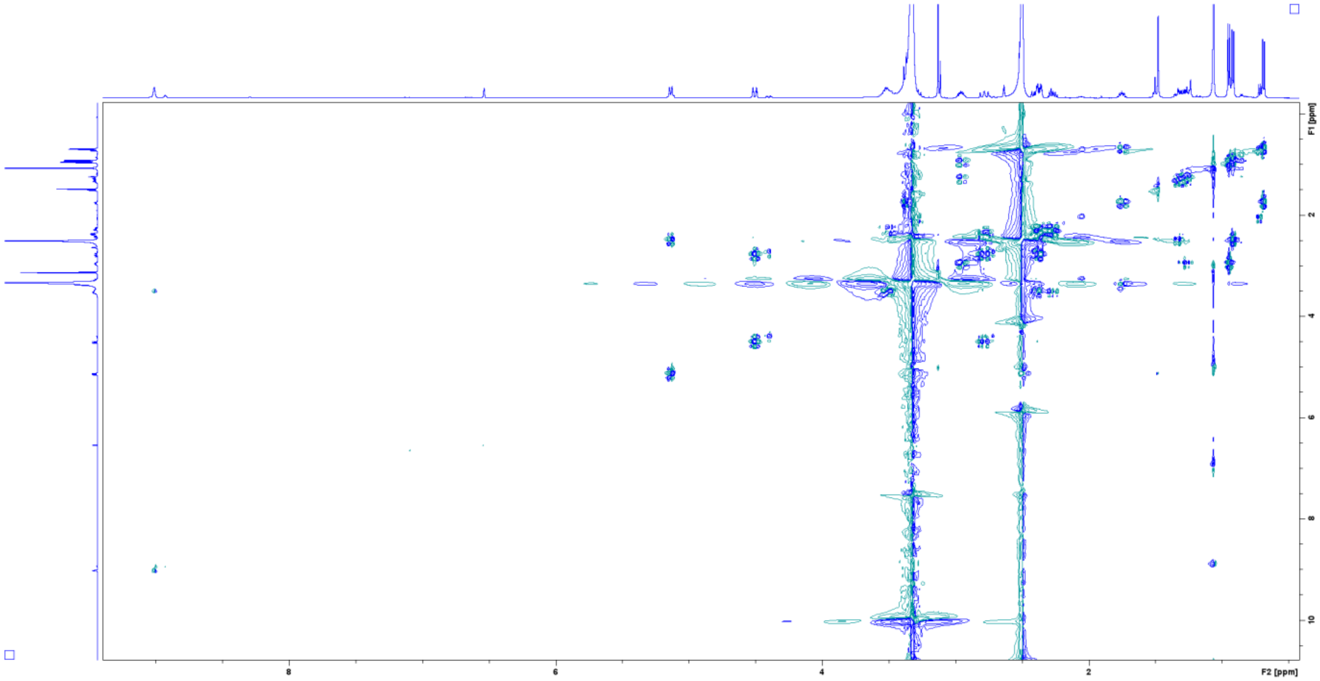

Figure S18. COSY spectrum of janustatin D (2) in DMSO-d_6 at 298 K.

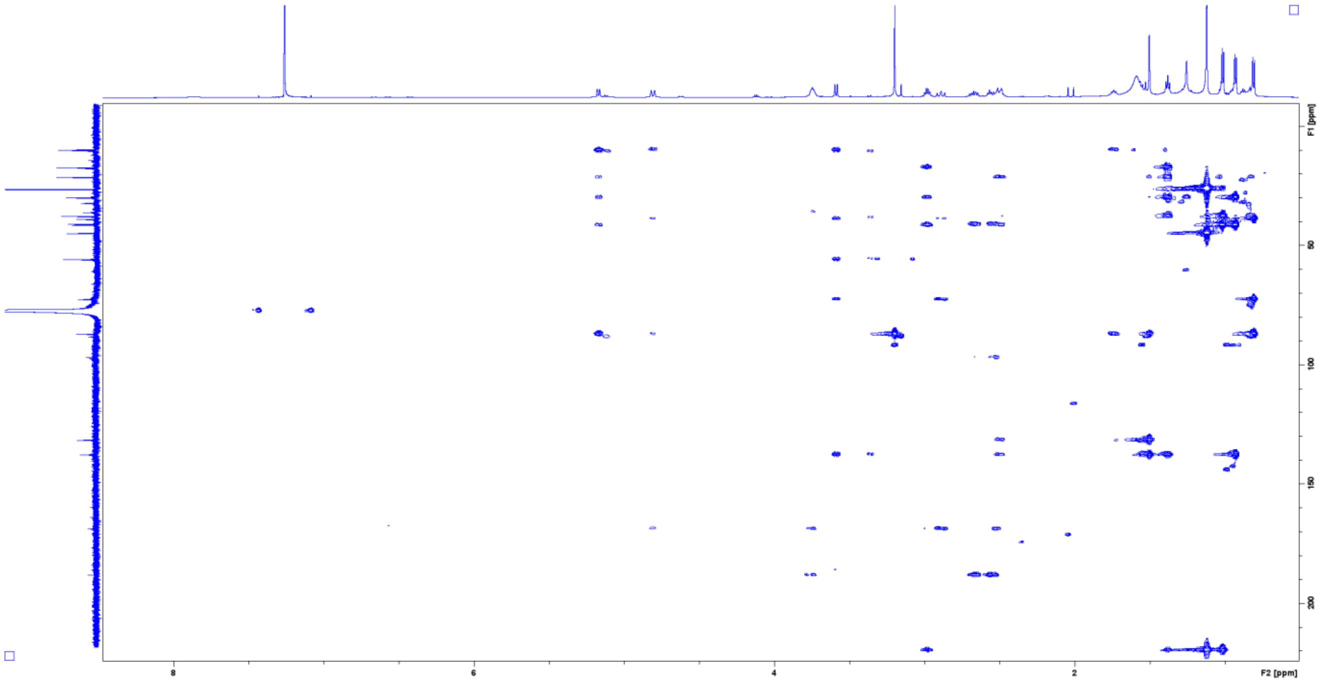

Figure S19. HMBC spectrum of janustatin D (2) in $CDCl_3$ at 298 K.

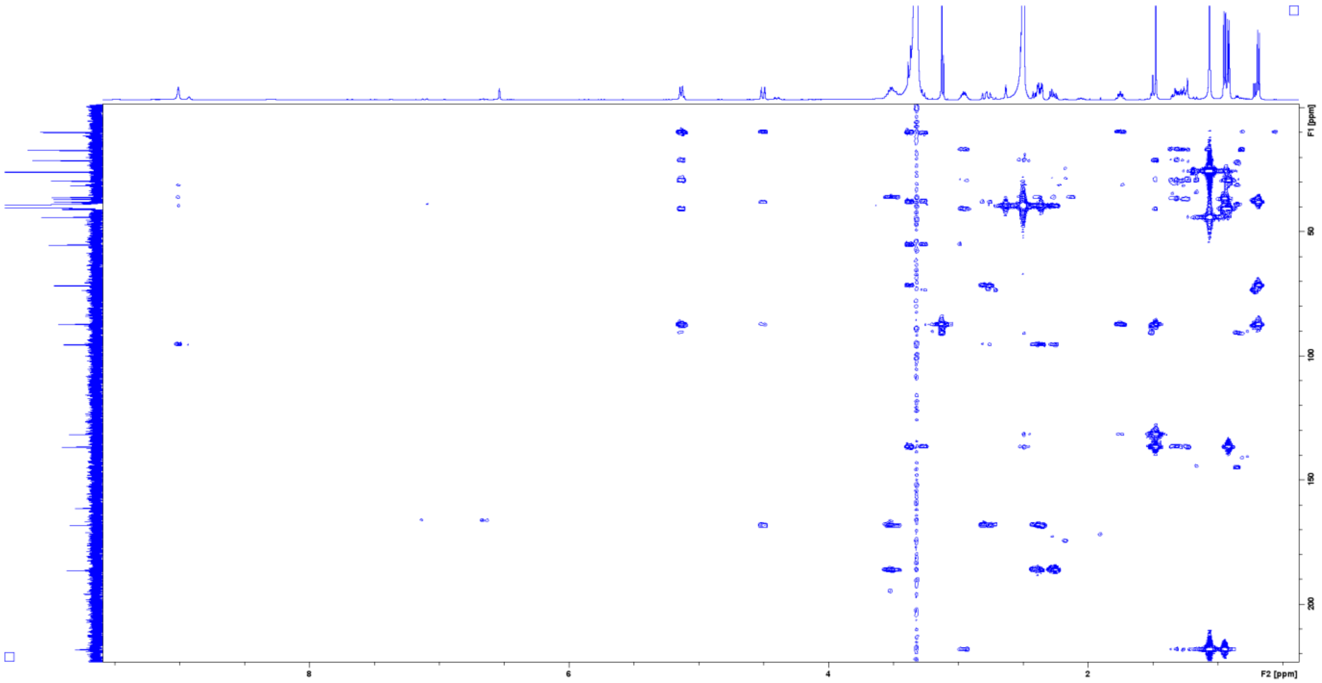

Figure S20. HMBC spectrum of janustatin D (2) in DMSO-d_6 at 298 K.

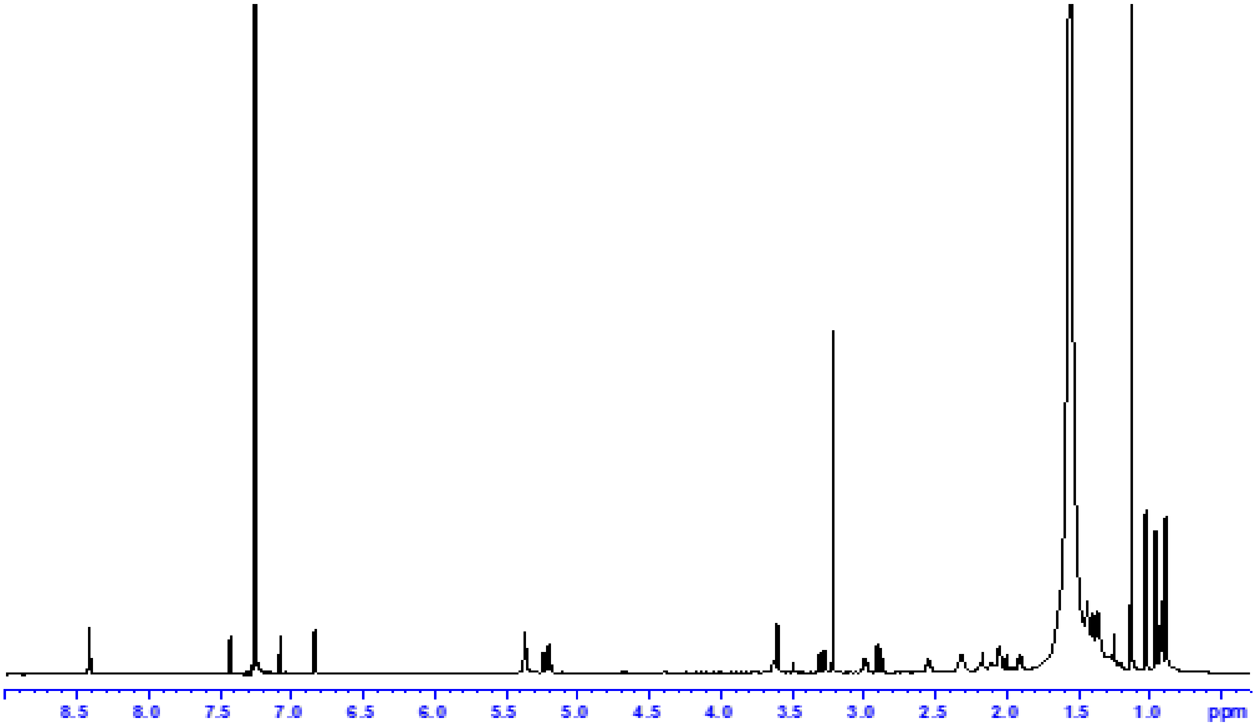

Figure S21. [1]**H NMR spectrum of janustatin E (3) in $CDCl_3$ at 298 K. (600 MHz)**

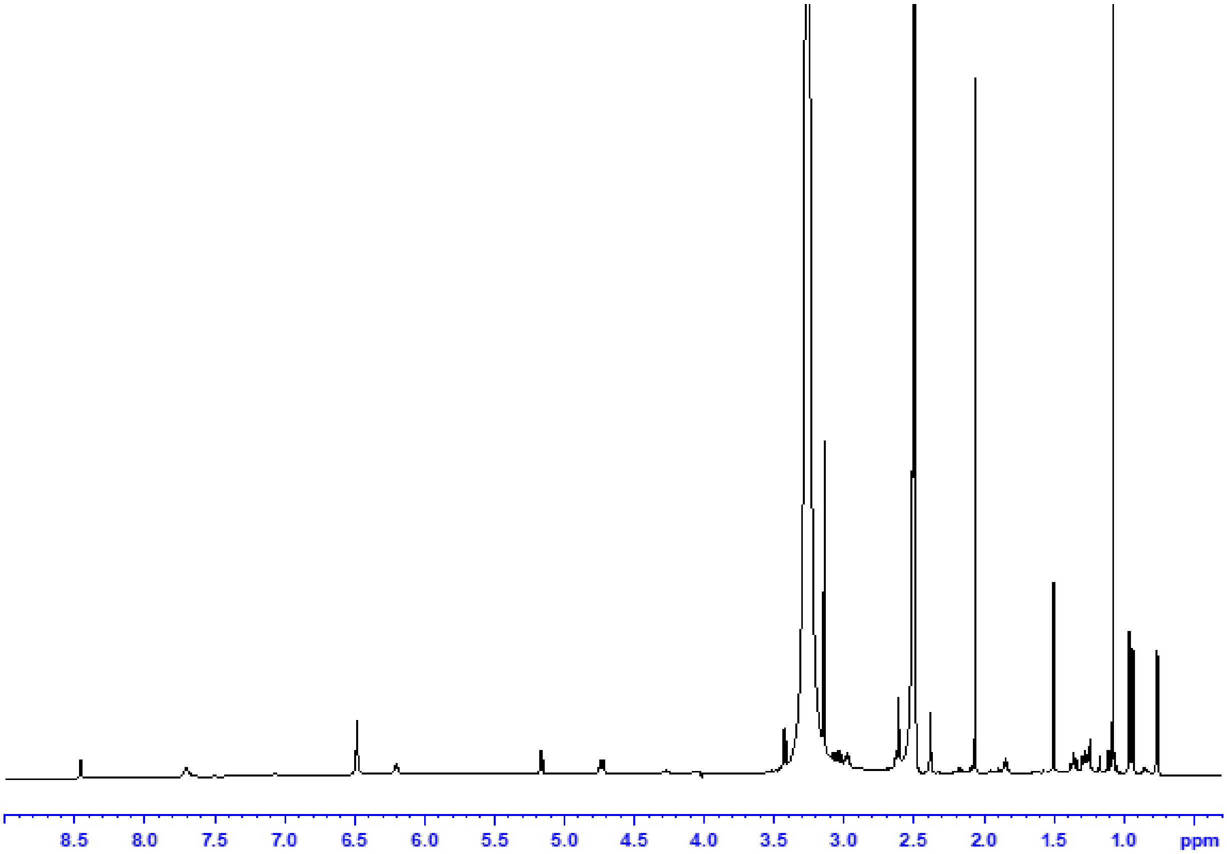

Figure S22. [1]**H NMR spectrum of janustatin E (3) in DMSO-*d*$_6$ at 298 K.**

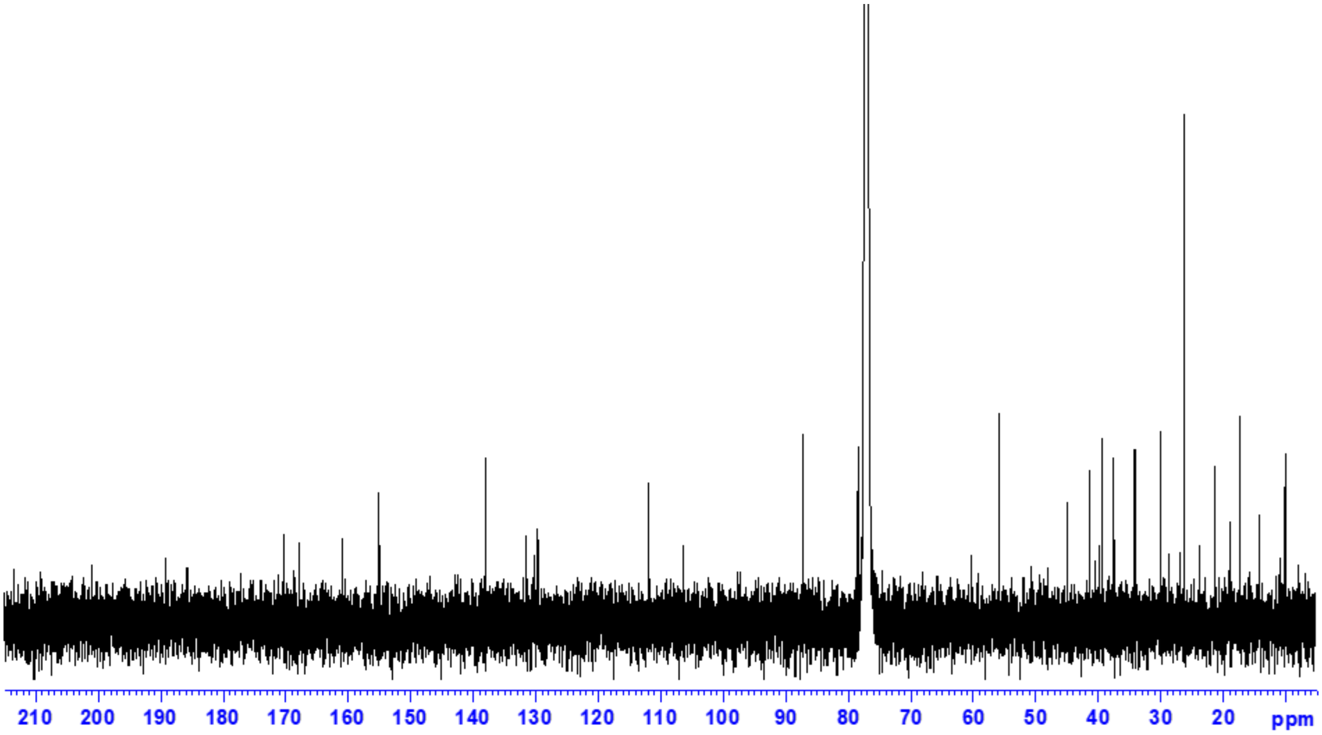

Figure S23. **^{1}H NMR spectrum of janustatin E (3) in $CDCl_3$ at 298 K (150 MHz).**

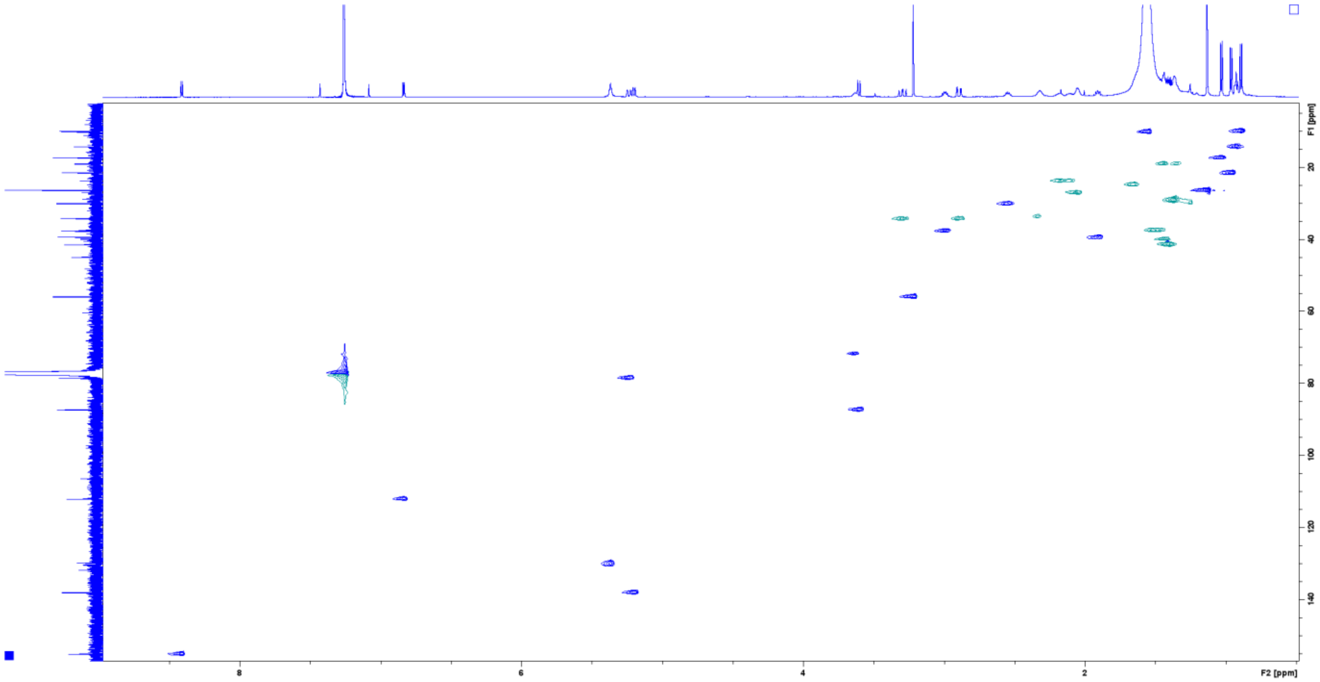

Figure S24. HSQC spectrum of janustatin E (3) in $CDCl_3$ at 298 K.

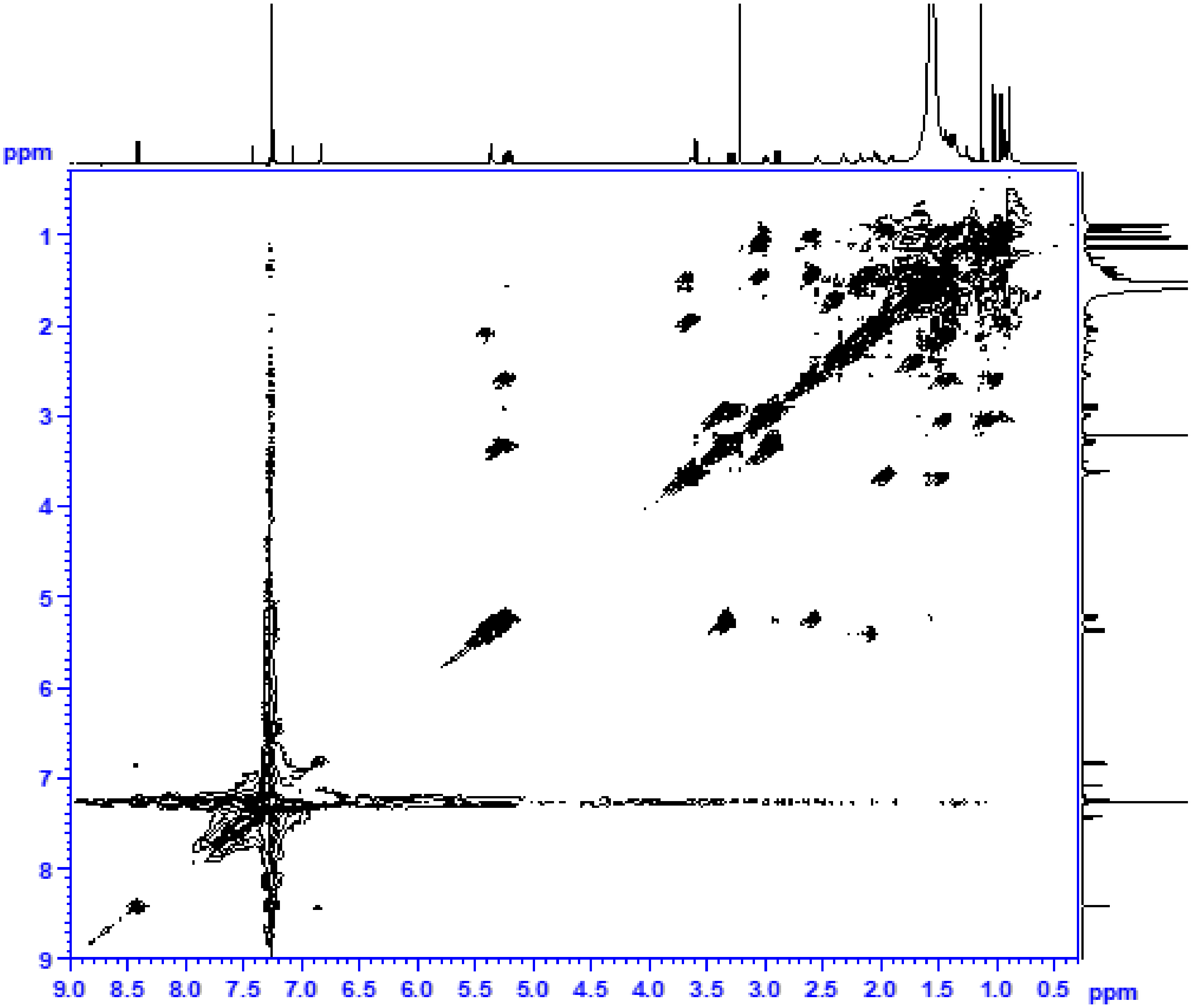

Figure S25. COSY spectrum of janustatin E (3) in $CDCl_3$ at 298 K.

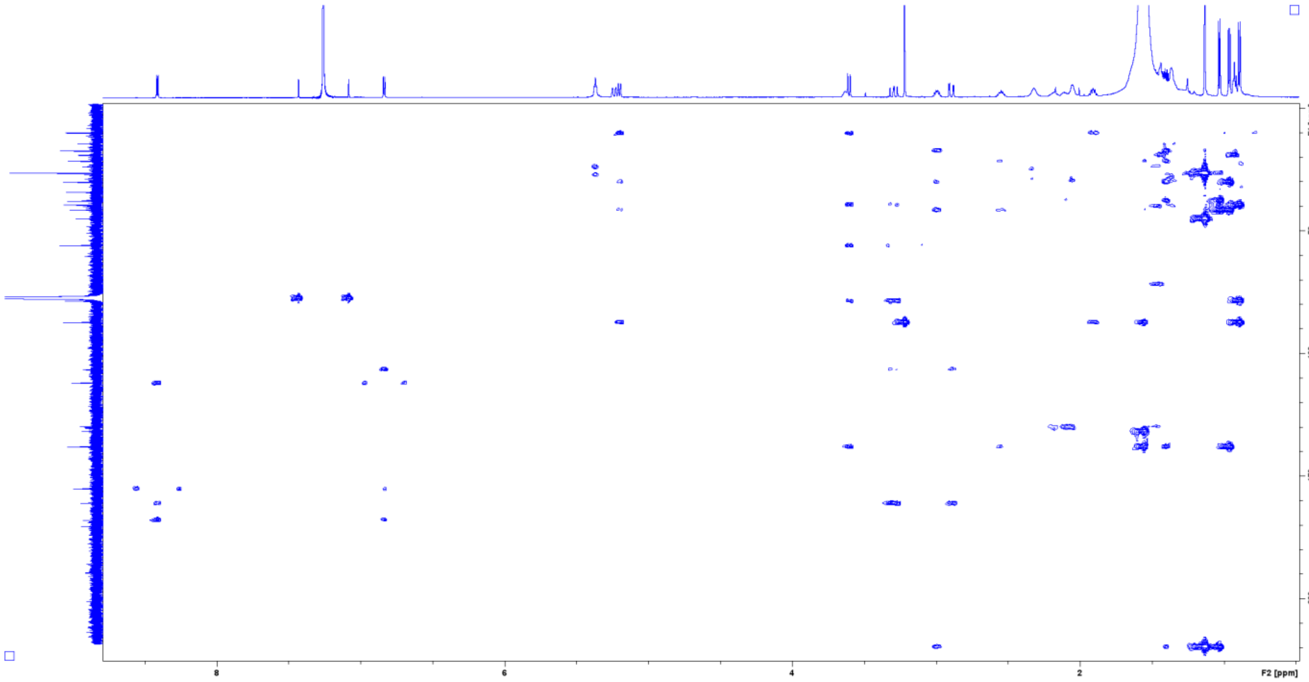

Figure S26. HMBC spectrum of janustatin E (3) in $CDCl_3$ at 298 K.

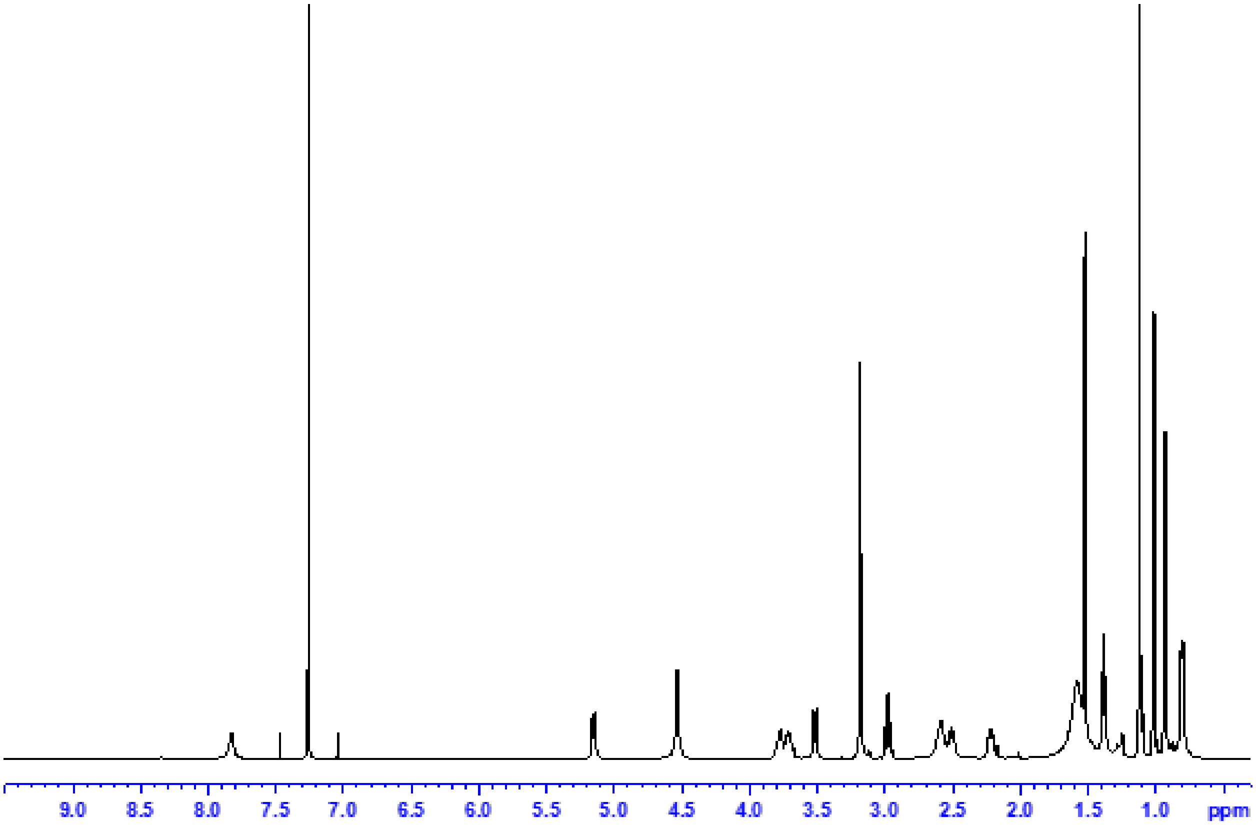

Figure S27. **^{1}H NMR spectrum of janustatin F (4) in $CDCl_3$ at 298 K (500 MHz).**

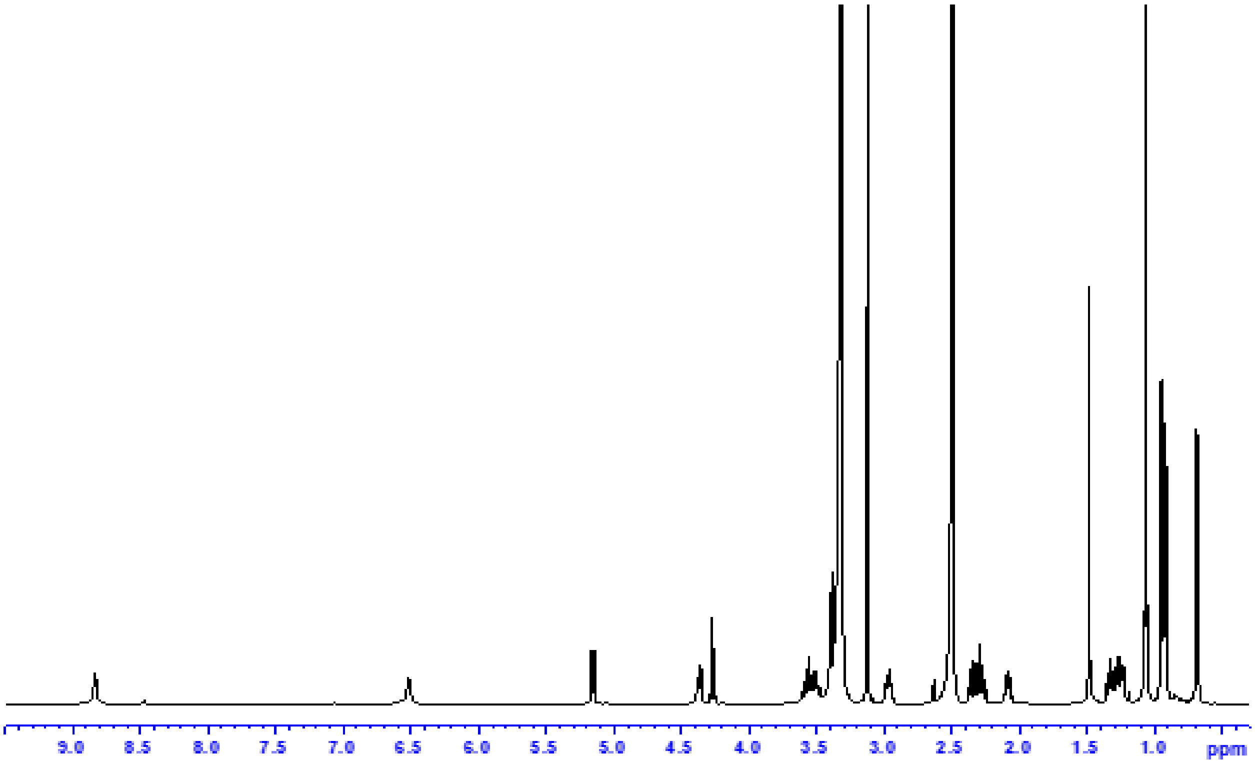

Figure S28. **^{1}H NMR spectrum of janustatin F (4) in DMSO-d_6 at 298 K (500 MHz).**

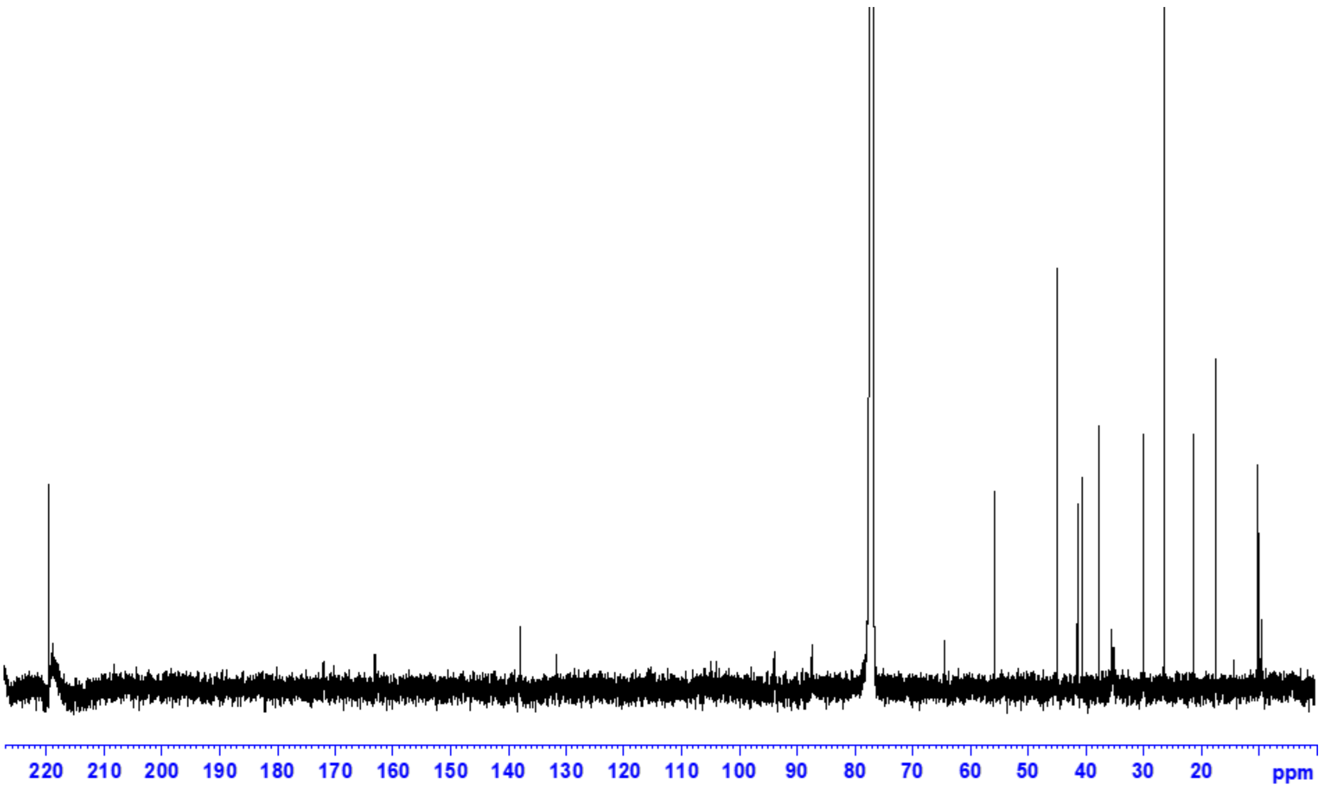

Figure S29. ^{13}**C NMR spectrum of janustatin F (4) in CDCl$_3$ at 298 K (125 MHz).**

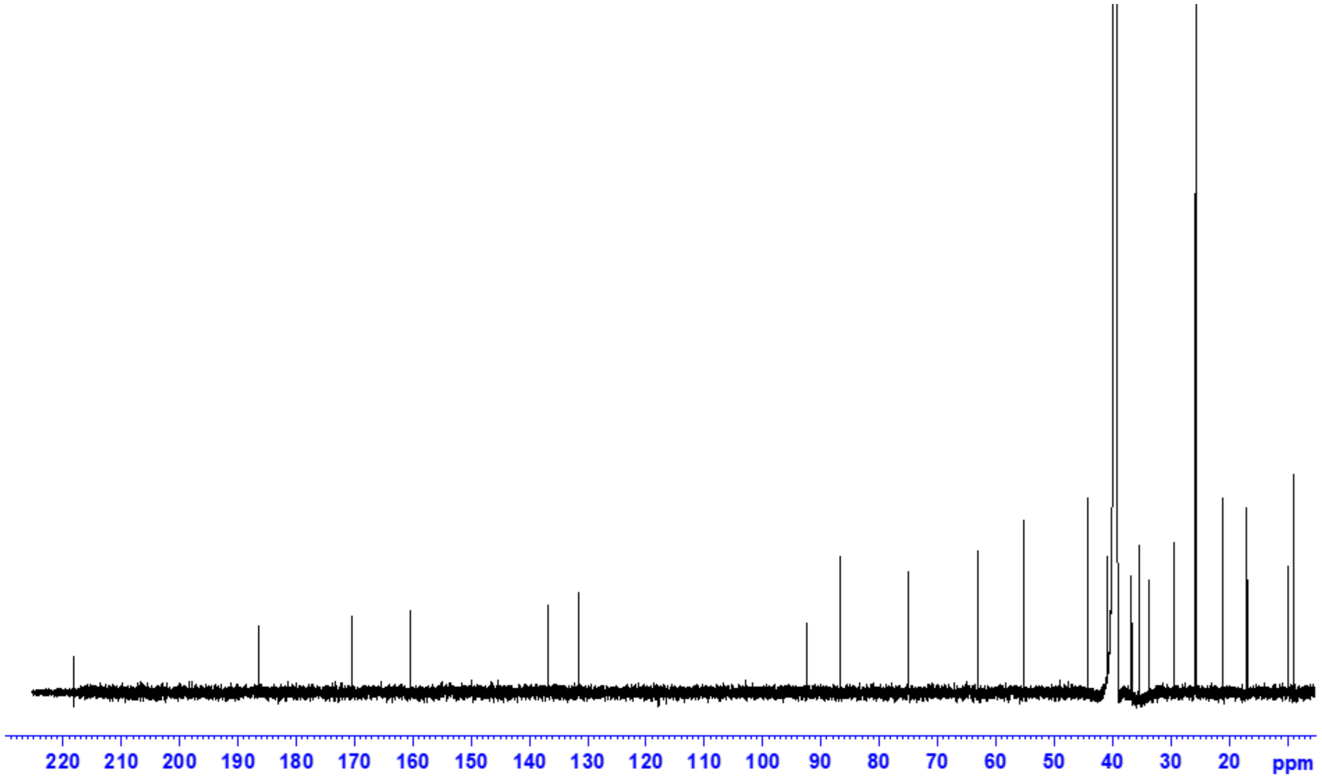

Figure S30. ^{13}**C NMR spectrum of janustatin F (4) in DMSO-d_6 at 298 K (175 MHz).**

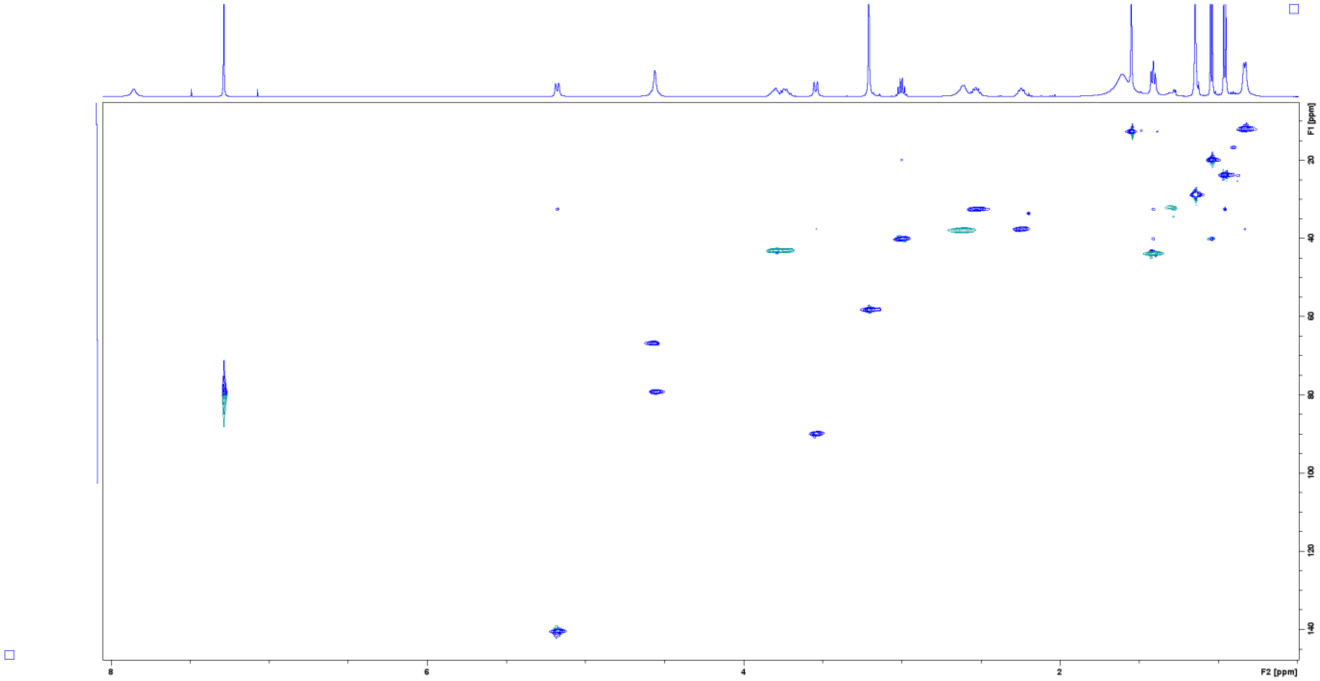

Figure S31. HSQC spectrum of janustatin F (4) in $CDCl_3$ at 298 K.

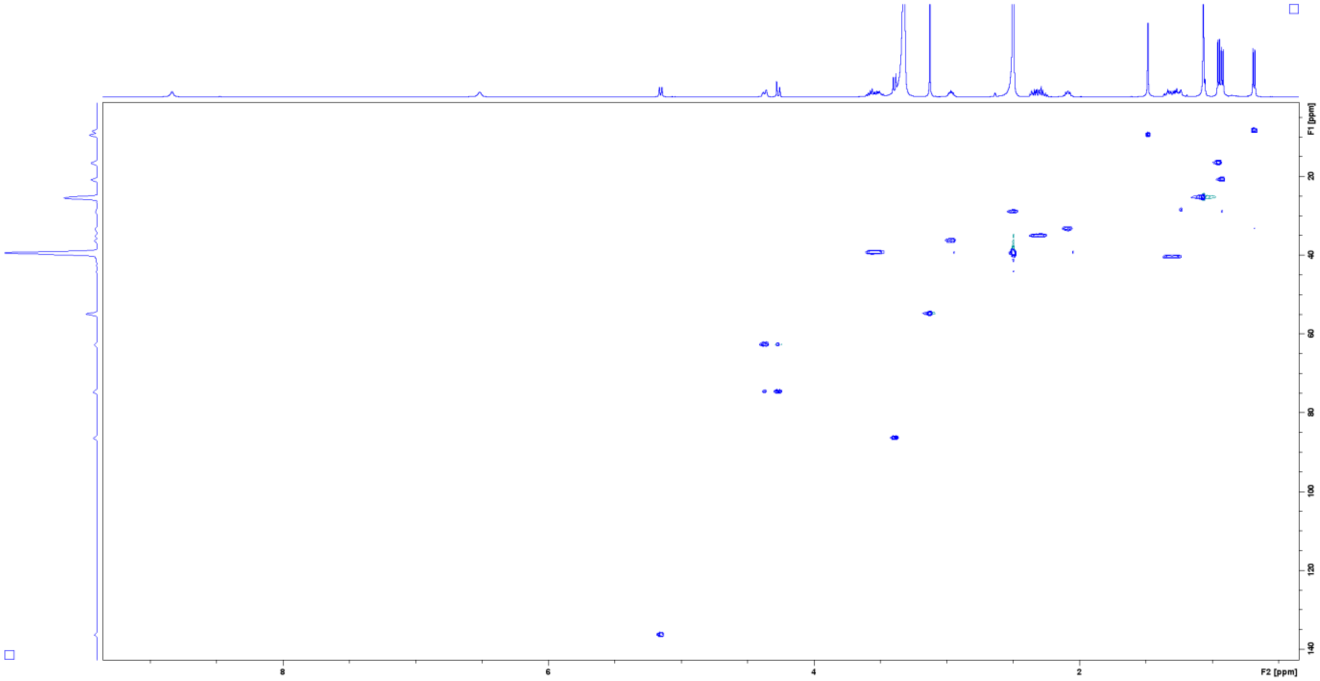

Figure S32. HSQC spectrum of janustatin F (4) in DMSO-d_6 at 298 K.

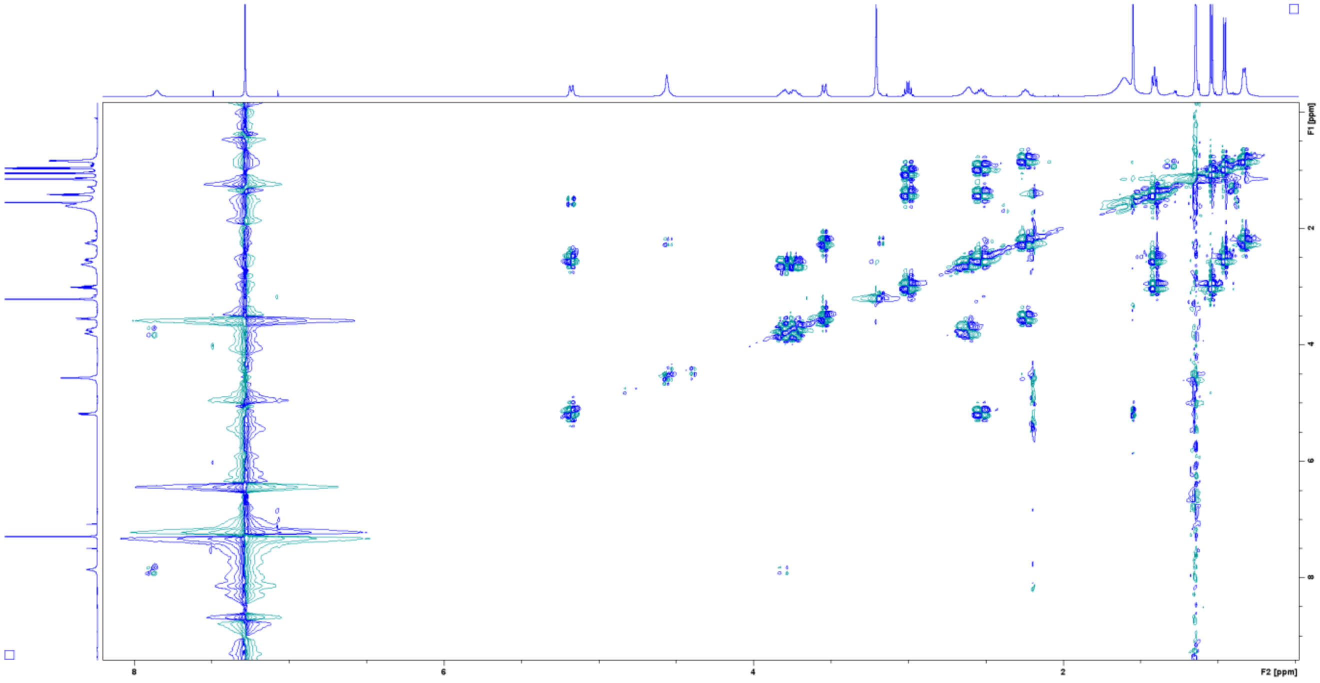

Figure S33. COSY spectrum of janustatin F (4) in $CDCl_3$ at 298 K.

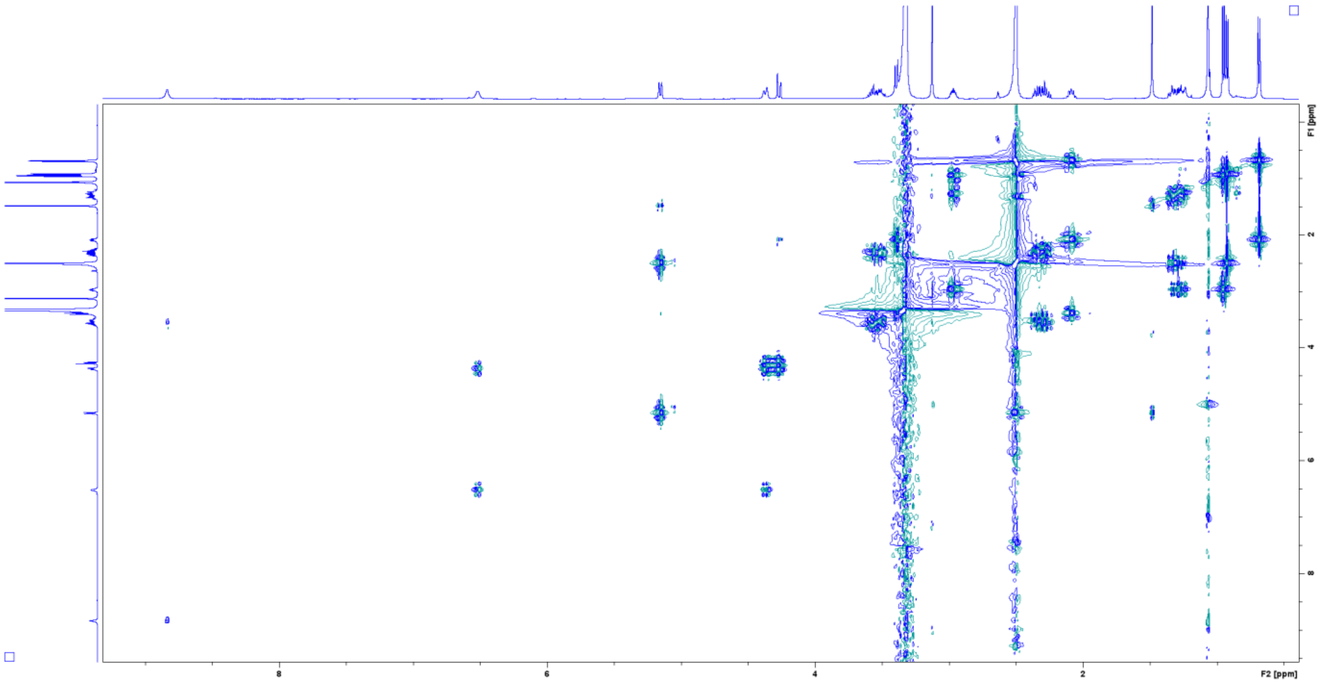

Figure S34. COSY spectrum of janustatin F (4) in DMSO-*d*$_6$ at 298 K.

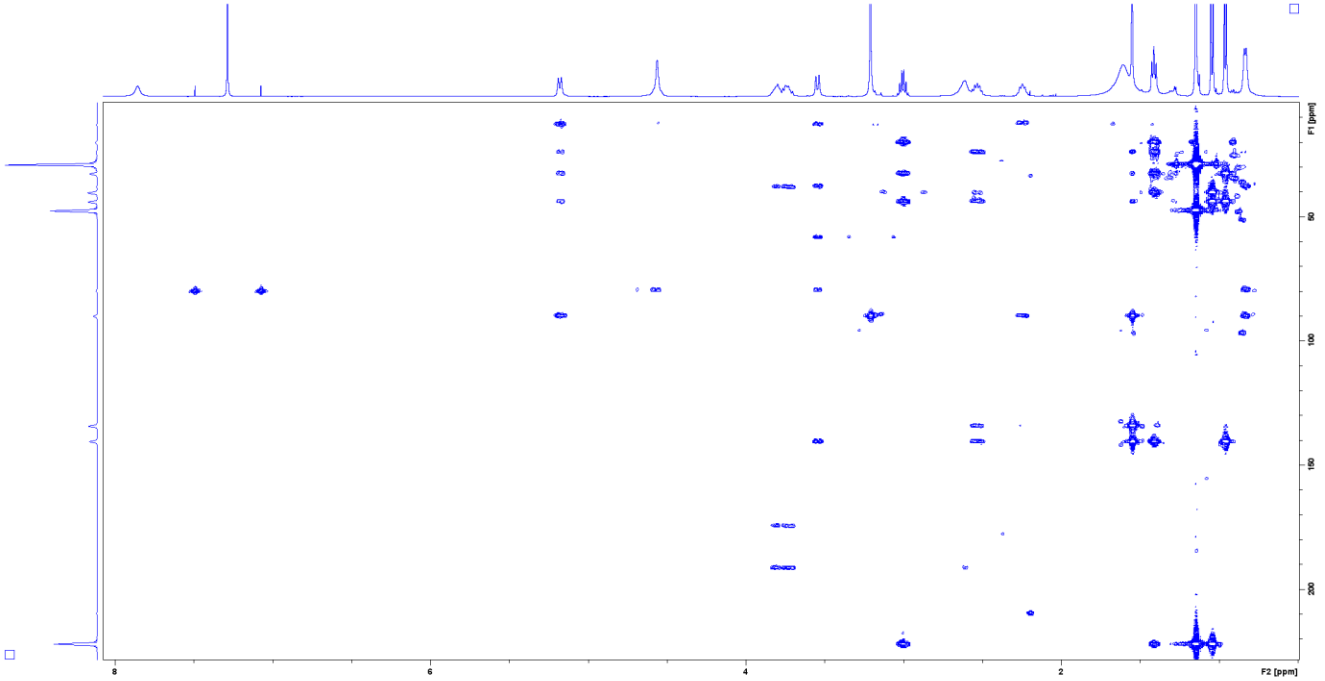

Figure S35. HMBC spectrum of janustatin F (4) in $CDCl_3$ at 298 K.

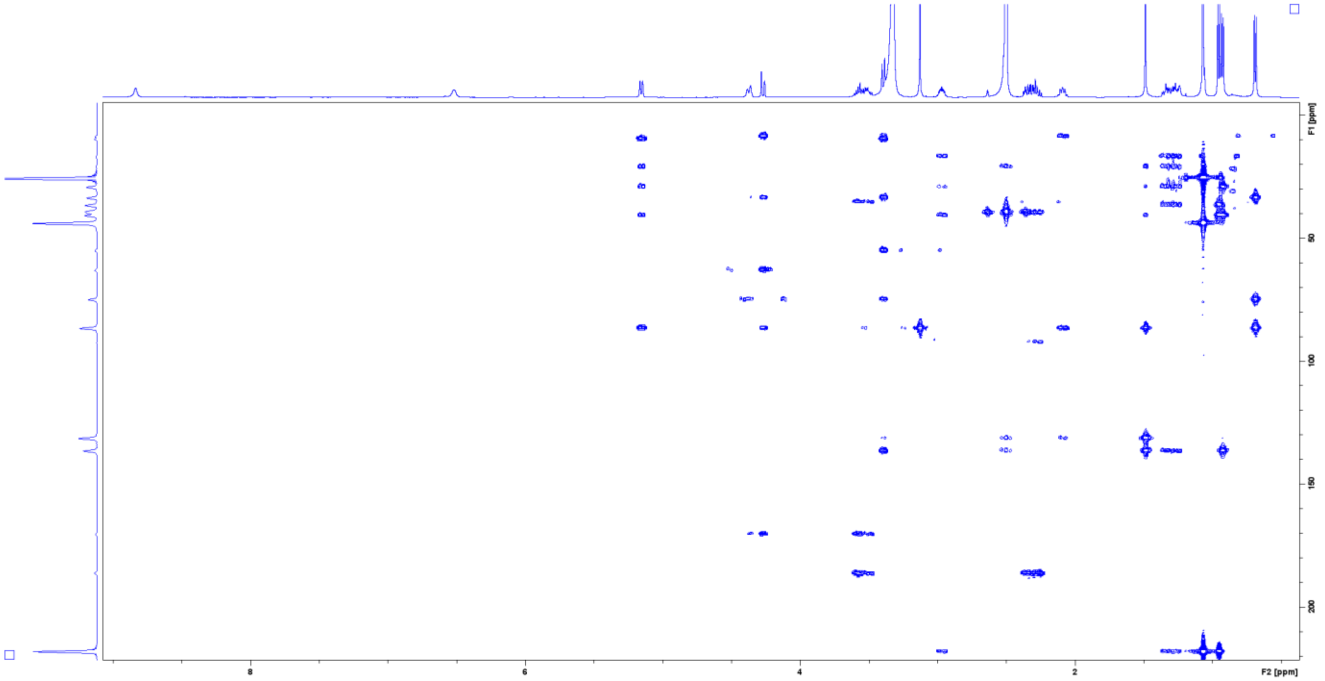

Figure S36. HMBC spectrum of janustatin F (4) in DMSO-d_6 at 298 K.

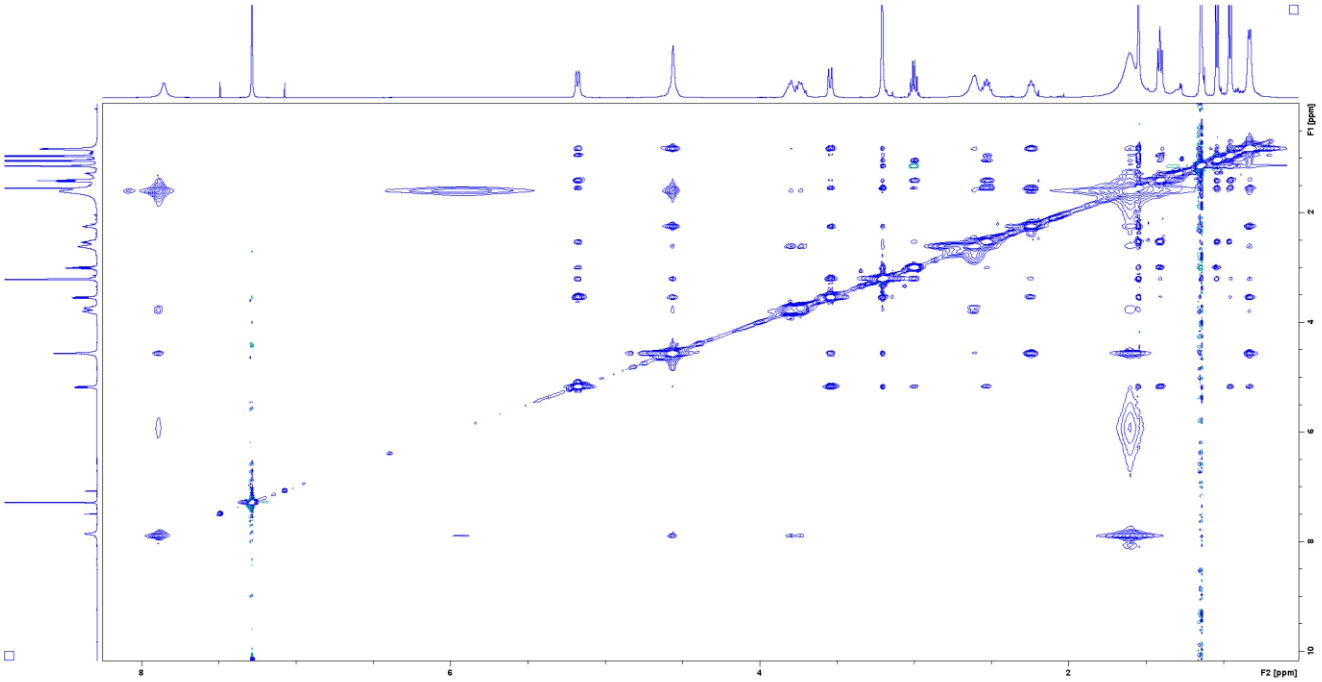

Figure S37. Nuclear Overhauser effect spectrum of janustatin F (4) in $CDCl_3$ at 298 K.

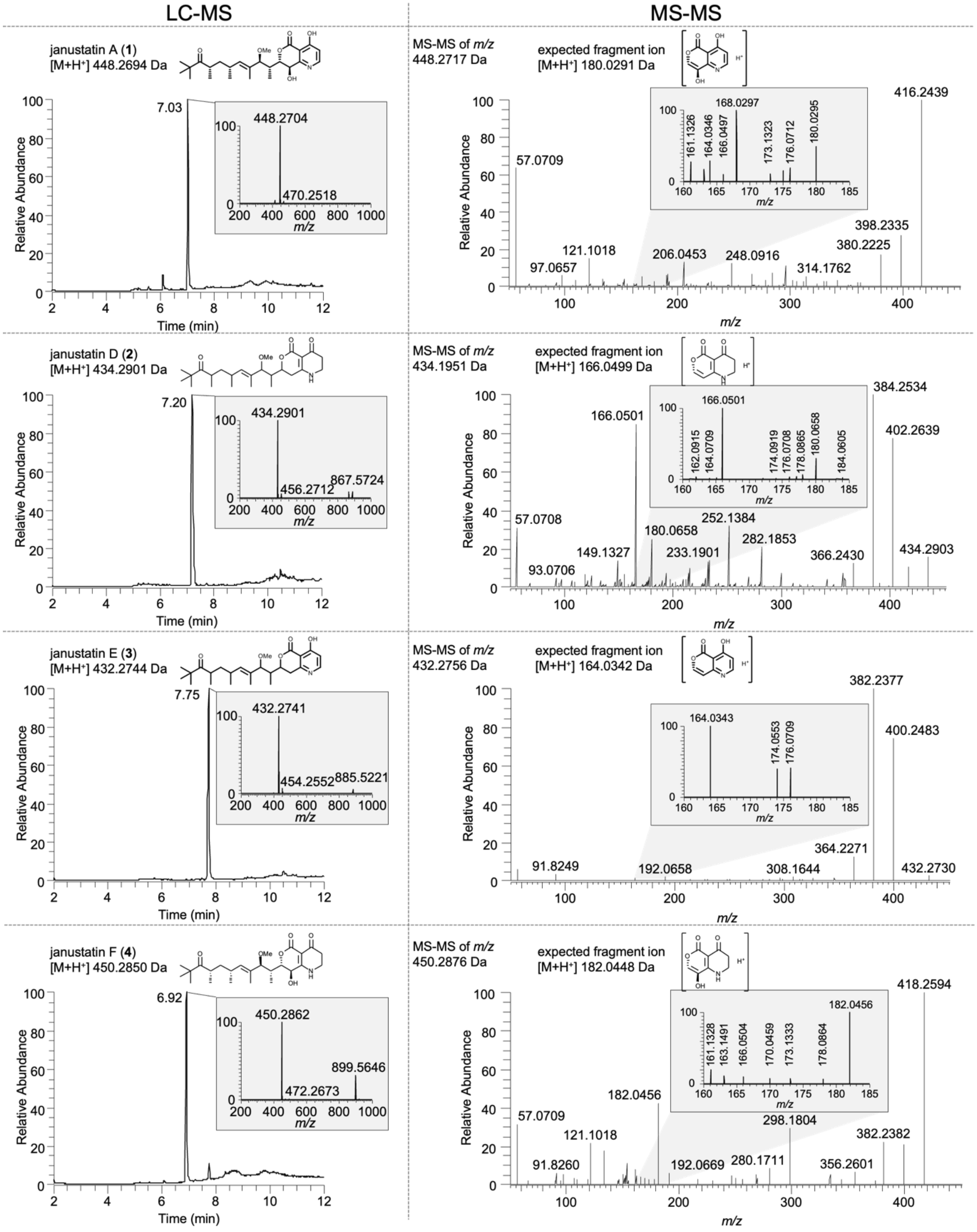

Figure S38. LC-MS traces of purified janustatins A, D, E, and F.

Left; Total ion chromatograms (*m/z* 200-1500 Da) of purified compounds **1**-**4**. High resolution mass spectra are shown in insets. From these, molecular formulas were calculated: janustatin A (**1**): $C_{25}H_{37}NO_6$; *m/z* 448.2704 $[M+H]^+$, Δ +1.03 mmu; janustatin D (**2**): $C_{25}H_{39}NO_5$; *m/z* 434.2903 $[M+H]^+$, Δ +0.18 mmu; janustatin E (**3**): $C_{25}H_{37}NO_5$; *m/z* 432.2742 $[M+H]^+$, Δ -0.25 mmu; janustatin F (**4**): $C_{25}H_{39}NO_6$; *m/z* 450.2863 $[M+H]^+$, Δ +1.24 mmu. Right; Tandem MS (MS/MS, isolation window: 1 Da) spectra of compounds **1**-**4**. Inset shows the region containing the fragments of the bicyclic system (*m/z* 160-185 Da).

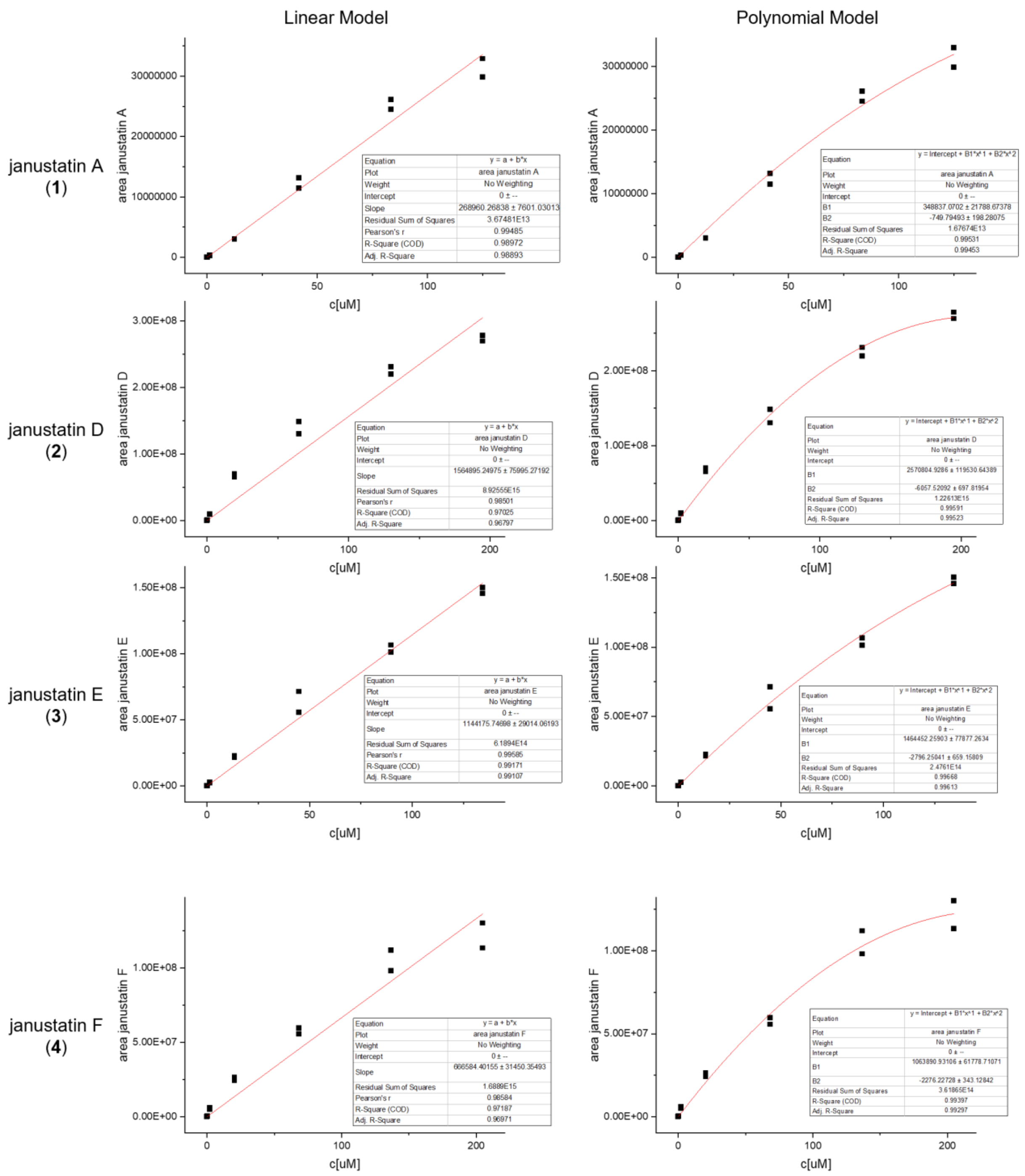

Figure S39. Fitting standard curves to janustatin standards.
In Origin Lab (OriginLab Corporation) a linear as well as a second order polynomial model were fitted to the data of janustatins (**1**-**4**). To estimate the concentrations of janustatins in the mutants the polynomial model was used (Figure S6-S8 and Table S6-S8)

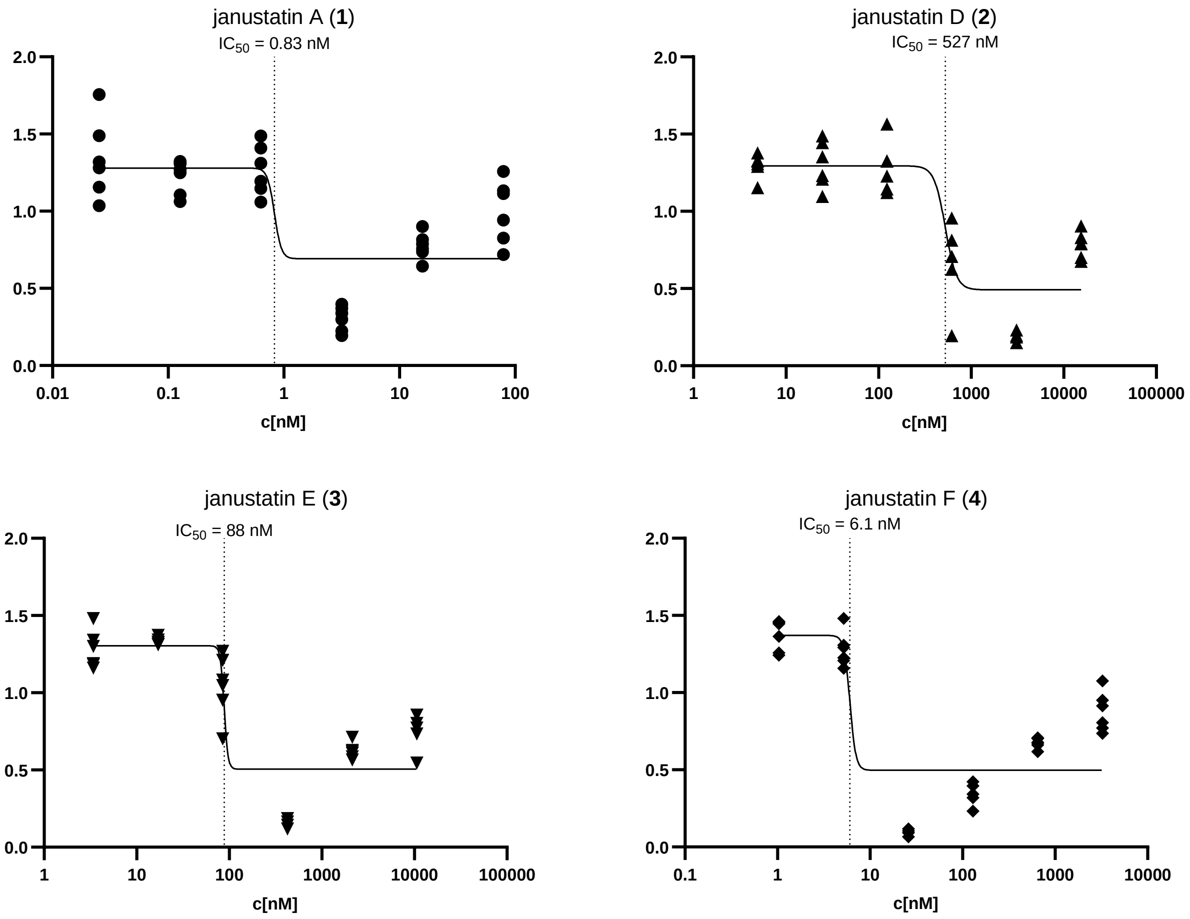

Figure S40. Cytotoxicity tests of compounds (1-4) against HeLa cells.
Calculated IC_{50}-values: **1**, 0.83 nM, R^2 = 0.57; **2**, 527 nM, R^2 = 0.75; **3**, 88 nM, R^2 = 0.752; **4**, 6.1 nM, R^2 = 0.70. Absorbance of MTT was measured at 570 nm. Data of six independent experiments performed on three different plates is shown. A four-parameter logistic function was used to fit the curve. The bottom and the IC_{50} were constrained to “greater than 0”. No outliers were detected using a ROUT coefficient Q=1% in GraphPad Prism 9.2.0.

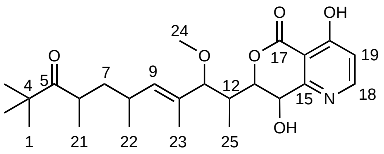

janustatin A (**1**)

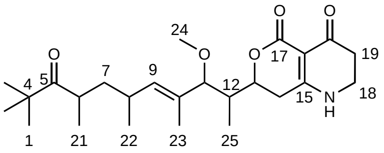

janustatin D (**2**)

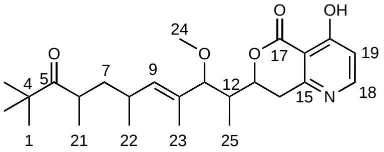

janustatin E (**3**)

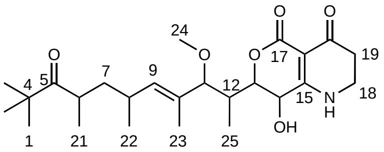

janustatin F (**4**)

Figure S41. Structures of janustatin A, D, E and F.
Chemical shifts are shown in Table S4 and Table S5. NMR correlations in Figure 4.

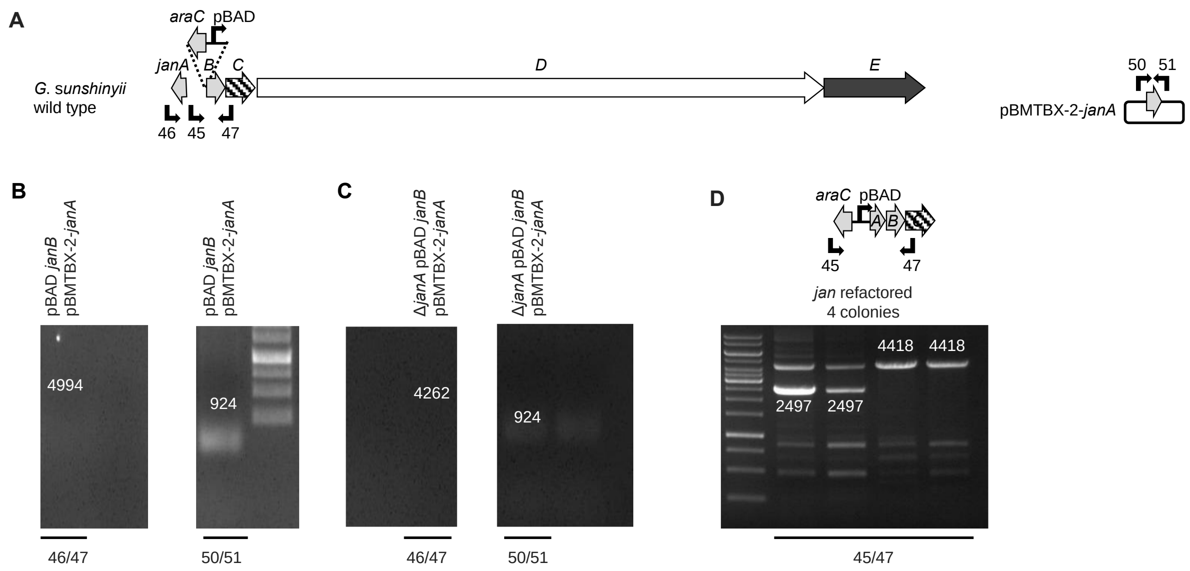

Figure S42. PCR-based genotyping of *G. sunshinyii* mutants.
A, Map of the janustatin BGC and the pBMTBX-2 plasmid detailing the regions where primers bind (not to scale). By homologous recombination, *araC* and the pBAD promoter were placed upstream of *janB* (dotted line). **B**, PCR products confirming integration of the induction system upstream of *janB* in the wild type. The 924 bp PCR product confirms the presence of pBMTBX-2 harboring *janA*. **C**, PCR products confirming integration of the induction system upstream of *janB* in the Δ*janA* mutant. The 924 bp PCR product confirm the presence of pBMTBX-2 harboring *janA*. **D**, The *jan* BGC was refactored by placing *araC*, the pBAD promoter and *janA* upstream of *janB* in the *janA* deletion mutant, increasing the length of the PCR product from 2497 to 4418. Expected PCR product sizes (in bp) are shown in white. PCR bands were purified and sequenced.

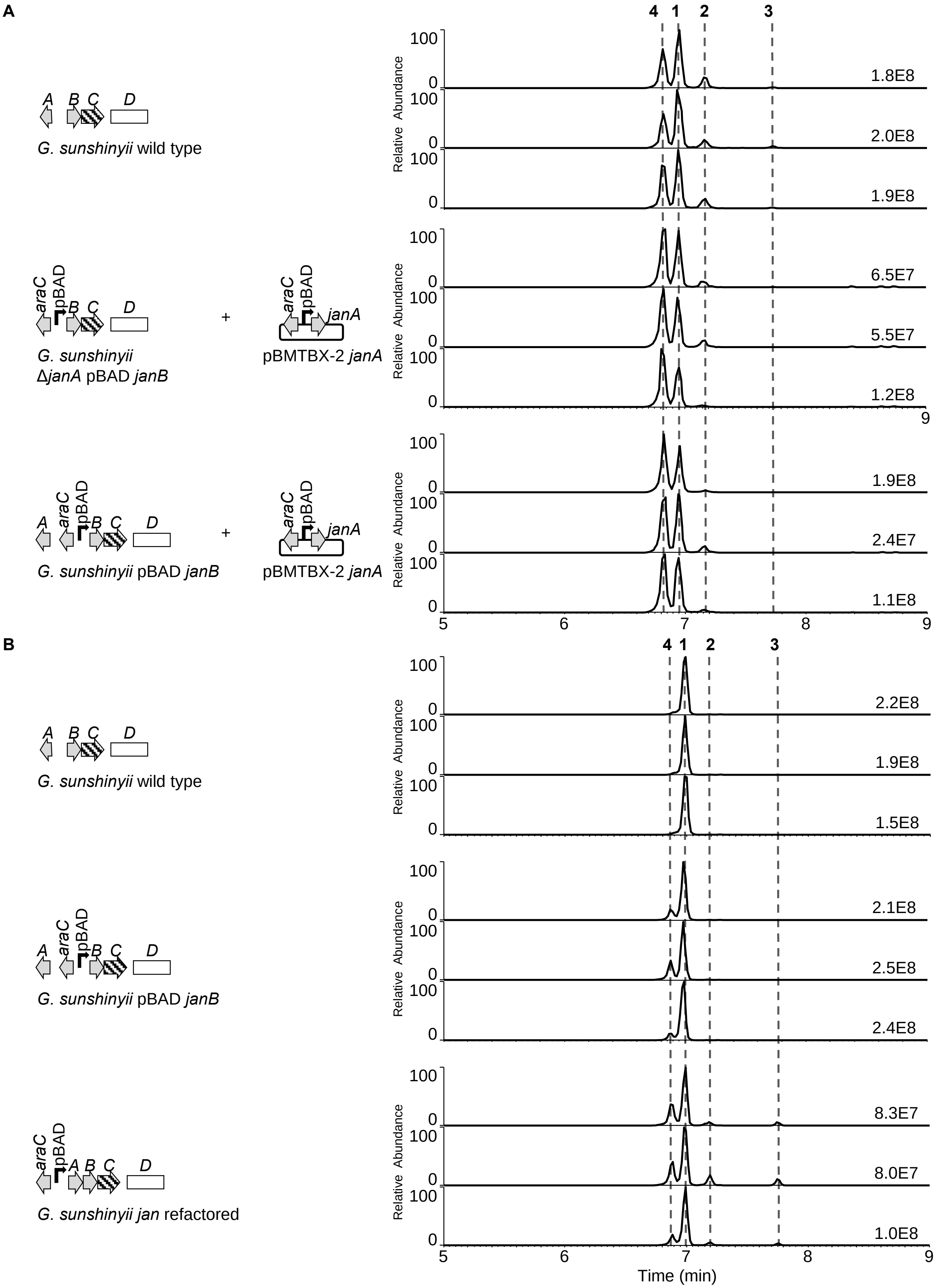

Figure S43. *G. sunshinyii* genome engineering to increase production of janustatin A.

Left: Schematic representation of genotypes. Right: Extracted ion chromatograms of LC-MS measurements of organic extracts from *G. sunshinyii* strains (*m/z* 432.2744, 434.2901, 448.2694, 450.2850). Compounds **2-4** are biosynthetic intermediates of **1**. **A**, The two strains *G. sunshinyii* pBAD *janB* and *G. sunshinyii* Δ*janA* pBAD *janB* were complemented with *janA* on the plasmid pBMTBX-2. **B**, The biosynthetic product of the *jan* BGC is detected in the two strains, *G. sunshinyii* pBAD *janB* and *G. sunshinyii jan* refactored. For quantification see Table S9.

Table S1. Primers used in this study.

Capitalized letters are binding sequences, small letters overhangs.

construct	ID	name	sequence (binding region capitalized)	template	size [bp]	comment
**pSW8197-Δ*janA***	1	Δ*janA* H1 f	ccggcgtcgacGAAGCATTGACCGATGCCATG	*G. sunshinyii*	520	
	2	Δ*janA* H1 r	tcattgaattcCAGGACGGTGCCGCGTTAAAC			
	3	Δ*janA* H2 f	tcctggaattcAATGACTGCCTCTACTCATG	*G. sunshinyii*	520	
	4	Δ*janA* H2 r	ctagaactagtTTTATTTCATATAAAGATGAC			
	5	Δ*janA* back f	aataaaACTAGTTCTAGAGCCGTC	pSW8197	3445	
	6	Δ*janA* back r	gcttcGTCGACGCCGGCCAGCCTCG			
**pSW8197-Δ*janB***	7	Δ*janB* H1 f	ccggcgtcgacAAATATTTCAGCTGCTGATAAG	*G. sunshinyii*	520	
	8	Δ*janB* H1 r	atcatgaattcCCGGCTTTCCTCGTTGATCG			
	9	Δ*janB* H2 f	gccgggaattcATGATTGAAGCATTGAATATG	*G. sunshinyii*	520	
	10	Δ*janB* H2 r	ctagaactagtGGTCGCATGAACATCTCTGC			
	11	Δ*janB* back f	cgaccactagtTCTAGAGCCGTC	pSW8197	3465	
	12	Δ*janB* back r	tatttgtcgacGCCGGCCAGCCTCG			
**pSW8197-Δpara**	13	Δpara H1 f	ccggcgtcgacACATGGTCTCCGGGCAGATC	*G. sunshinyii*	520	
	14	Δpara H1 r	agcgcgaattcCGGCATTTCCACCACCACGG			
	15	Δpara H2 f	tgccggaattcGCGCTTTGAAGAGCAGGCAC	*G. sunshinyii*	520	
	16	Δpara H2 r	ctagaactagtGGACAAATTCAGTTCCCG			
	17	Δpara back f	tgtccactagtTCTAGAGCCGTC	pSW8197	3445	
	18	Δpara back r	catgtgtcgacGCCGGCCAGCCTCG			
**pSW8197 to pEB17 backbone exchange**	19	pEB17 H1H2 f	gagtcgacctTCAACGGGAATCCTGCTC	pSW8197 constructs	1113	construction of pEB17-Δ*janA*, pEB17-Δ*janB*, pEB17-Δpara
	20	pEB17 H1H2 r	atgggaaTTCTTGGTAACGAATCAGACAATTG			
	21	pEB17 back f	tcgttaccaaGAATTCCCATGTCAGCCG	pEB17	5311	
	22	pEB17 back r	ttcccgttgaAGGTCGACTCTAGAGGATC			
**pEB17-pBAD *janB***	23	pBAD *janB* back f	ctgtccgggtGAATTCCCATGTCAGCCG	pEB17	5291	
	24	pBAD *janB* back r	tgaaatatttGGTCGACTCTAGAGGATC			
	25	pBAD *janB* H1 f	agagtcgaccAAATATTTCAGCTGCTGATAAGTTTCTG	*G. sunshinyii*	500	
	26	pBAD *janB* H1 r	gttgtcataaCCGGCTTTCCTCGTTGATC			
	27	pBAD *janB* ins f	ggaaagccggTTATGACAACTTGACGGC	pBMTBX-2	1189	
	28	pBAD *janB* ins r	tttgagccatATGGAGAAACAGTAGAGAG			
	29	pBAD *janB* H2 f	gtttctccatATGGCTCAAATTATGAAAAGTG	*G. sunshinyii*	500	
	30	pBAD *janB* H2 r	atgggaattcACCCGGACAGAATTGAAC			
**pEB17-Δ*janB* pBAD *janC***	31	Δ*janB* pBAD *janC* back f	tcatgcgaccGAATTCCCATGTCAGCCG	pEB17-pBAD *janB*	5291	
	32	Δ*janB* pBAD *janC* back r	cttcaatcatATGGAGAAACAGTAGAGAGTTG			
	33	Δ*janB* pBAD *janC* H2 f	gtttctccatATGATTGAAGCATTGAATATGC	*G. sunshinyii*	500	
	34	Δ*janB* pBAD *janC* H2 r	atgggaattcGGTCGCATGAACATCTCTG			
**pBMTBX-2-*janA***	35	pBMTBX2 *janA* f	agcaccgcctATGTATGTAAGCTTTTTTGTTTTTCTGGC	*G. sunshinyii*	752	
	36	pBMTBX2 *janA* r	ttttaccatgTCACTCGGGTACCTTGGC			
	37	pBMTBX2 back f	acccgagtgaCATGGTAAAATGGTCAGTATTGAGCG	pBMTBX-2	5047	

	38	pBMTBX2 back r	ttacatacatAGGCGGTGCTAGCCCAAA			
**pBMTBX-2-*janB***	39	pBMTBX2 *janB* f	agcaccgcctATGGCTCAAATTATGAAAAGTGC	*G. sunshinyii*	953	
	40	pBMTBX2 *janB* r	ttttaccatgTCAATCATGCTCACCCGATAC			
	41	pBMTBX2 back f	gcatgattgaCATGGTAAAATGGTCAGTATTGAGCG	pBMTBX-2	5047	
	42	pBMTBX2 back r	tttgagccatAGGCGGTGCTAGCCCAAA			
**primers to confirm mutants**	43	pEB17 upstream	CTAAATAATAGTGAACGGCAGGTATATG	*G. sunshinyii* mutants		binds on pEB17 confirmation of single recombination event (with primers 47, 49)
	44	pEB17 downstream	GGATGTAACGCACTGAGAA			binds on pEB17 confirmation of single recombination event (with primers 45, 46, 48)
	45	*janB* upstream	tcctggaattcAATGACTGCCTCTACTCATG	*G. sunshinyii* mutants		binds upstream of janB facing downstream
	46	*janA* upstream	GTCATGGGGGTATTCCAGGCC	*G. sunshinyii* mutants		binds upstream of janA facing downstream
	47	*janB* downstream	TGCTGTGCCAGTTCAGCCTGTTC			binds downstream of janB facing upstream
	48	para upsteamr	CATTAATGAAAGTCTGGGTGGTCAAAC	*G. sunshinyii* mutants		binds upstream of para facing downstream
	49	para downstream	GAATACAATCAACGCTATCTATCTTGAGG			binds downstream of para facing upstream
	50	pBMTBX-2 f	CTTTGCTATGCCATAGCATTTTTATCC	*G. sunshinyii* carrying pBTMBX-2 plasmids	241 (for empty plasmid)	detection of pBMTBX-2 plasmids
	51	pBMTBX-2 r	AGGCTCGTCCTGAATGATATCAAG			
**pEB17-refactored**	52	*jan* refactored back f	ttacatacatATGGCTCAAATTATGAAAAGTG	pEB17-pBAD *janB*	7450	**pEB17-refactored**
	53	*jan* refactored back r	acccgagtgaTGGAGAAACAGTAGAGAG			
	54	*jan* refactored *janA* f	tgtttctccaTCACTCGGGTACCTTGGC	*G. sunshinyii*	752	
	55	*jan* refactored *janA* r	tttgagccatATGTATGTAAGCTTTTTTGTTTTTCTGGC			

Table S2. *G. sunshinyii* mutants generated in this study.
New mutants were generated by conjugative transfer of a suicide plasmid from the donor strain to the respective acceptor strain.

***G. sunshinyii* mutant**	**donor: E. coli ST18 carrying plasmid**	**acceptor strain (*G. sunshinyii*)**	**purpose**
Δ*janA*	pEB17-Δ*janA*	wild type	determining function of JanA
Δ*janB*	pEB17-Δ*janB*	wild type	determining function of JanB
Δ*para*	pEB17-Δ*para*	wild type	determining function of the para domain
Δ*janAB*	pEB17-Δ*janB*	Δ*janA*	determining function of JanA and JanB
Δ*janA* pBMTBX-2-empty	pBMTBX-2-empty	Δ*janA*	determining function of JanA
Δ*janB* pBMTBX-2-empty	pBMTBX-2-empty	Δ*janB*	determining function of JanB
Δ*janA* pBMTBX-2-*janA*	pBMTBX-2-*janA*	Δ*janA*	determining function of JanA
Δ*janB* pBMTBX-2-*janB*	pBMTBX-2-*janB*	Δ*janB*	determining function of JanB
Δ*janAB* pBAD *janC*	pEB17-Δ*janB* pBAD *janC*	Δ*janA*	production of janustatin D (**1**)
Δ*janA* pBAD *janB*	pEB17-pBAD *janB*	Δ*janA*	production of janustatin E (**2**)
Δ*janB* pBAD *janC*	pEB17-Δ*janB* pBAD *janC*	wild type	production of janustatin F (**3**)
ΔpBAD *janB*	pEB17-pBAD *janB*	wild type	production of janustatin A
jan refactored	pEB17-*jan* refactored	Δ*janA*	production of janustatin A
ΔpBAD *janB* pBMTBX-2-*janA*	pBMTBX-2-*janA*	ΔpBAD *janB*	production of janustatin A
Δ*janA* ΔpBAD *janB* pBMTBX-2-*janA*	pBMTBX-2-*janA*	Δ*janA* ΔpBAD *janB*	production of janustatin A

Table S3. Relative quantification of janustatins (1-4) in *G. sunshinyii* Δpara.

Values are color-coded for each compound (**1**-**4**) from least amount (red) to highest amount (green). Standard curves: Figure S39 and Table S6.

	janustatin integrated areas				janustatin concentrations			
	A (1)	D (2)	E (3)	F (4)	A (1)	D (2)	E (3)	F (4)
***G. sunshinyii* strain**	**area [u]**	**area [u]**	**area [u]**	**area [u]**	**c [uM]**	**c [uM]**	**c [uM]**	**c [uM]**
wild type 1	1.9 E+6	3.2 E+5	6.0 E+5	3.5 E+5	1.6 E-1	3.8 E-3	1.2 E-2	9.7 E-3
wild type 2	2.3 E+6	4.2 E+5	6.6 E+5	3.5 E+5	2.0 E-1	4.9 E-3	1.4 E-2	9.8 E-3
wild type 3	2.7 E+6	5.1 E+5	9.2 E+5	3.9 E+5	2.3 E-1	6.0 E-3	1.9 E-2	1.1 E-2
Δpara 1	7.0 E+2	1.3 E+4	6.0 E+2	2.6 E+2	6.0 E-5	1.5 E-4	1.2 E-5	7.4 E-6
Δpara 2	6.1 E+2	5.5 E+4	9.8 E+2	6.0 E+3	5.3 E-5	6.4 E-4	2.0 E-5	1.7 E-4
Δpara 3	4.0 E+2	1.0 E+3	4.9 E+2	1.8 E+2	3.5 E-5	1.2 E-5	1.0 E-5	5.2 E-6

Table S4. NMR shift comparison of the published janustatin A (1) and janustatins D-F (2-4) in chloroform-*d*$_3$. Compare Figure 41. n/d: not detected.

	janustatin A (**1**)			janustatin D (**2**)			janustatin E (**3**)			janustatin F (**4**)		
No.	δ_C	δ_H	mult. J	δ_C	δ_H	mult. J	δ_C	δ_H	mult. J	δ_C	δ_H	mult. J
1	26.1	1.13	s	26.3	1.12	s	26.3	1.13	s	26.3	1.12	s
2	26.1	1.13	s	26.3	1.12	s	26.3	1.13	s	26.3	1.12	s
3	26.1	1.13	s	26.3	1.12	s	26.3	1.13	s	26.3	1.12	s
4	44.7			44.9			44.9			44.9		
5	219.3			219.7			219.5			219.6		
6	37.4	3	m	37.6	2.99	m	37.6	3	m	37.6	2.98	m
7	41.2	1.4	m	41.4	1.38	m	41.4	1.4	m	41.4	1.39	m
8	29.8	2.57	m	30	2.5	m	30	2.56	m	30	2.5	m
9	137.5	5.2	d, 9.8	137.8	5.18	d, 9.5	138	5.2	d, 9.7	138	5.16	d, 9.5
10	131.9			131.5			131.7			131.9		
11	86.7	3.6	d, 10.4	87.1	3.6	d, 10.0	87.3	3.61	d, 10.2	87.4	3.53	d, 10.1
12	34.8	2.4	m	38.9	1.77	m	39.3	1.93	m	35.2	2.24	m
13	82.1	4.93	dd, 1.4, 11.5	72.4	4.82	dt, 1.9, 2.3, 13.2	78.5	5.25	ddd, 1.4, 2.9, 13.5	77.0	4.54	bs
14	64.3	4.79	d, 11.5	32.5	2.51	m	34.2	2.91	dd, 3.1, 16.9	64.4	4.54	bs
					2.92	dd, 12.9, 16.3		3.3	dd, 13.6, 16.8			
15	161.6			168.3			161			172.1		
16	103.9			96.9			106.3			93.9		
17	168.5			n/d			170.4			n/d		
18	154.5	8.49	d, 5.9	41.2	3.75	dd, 6.8, 4.5	155.1	8.42	d, 5.9	40.7	3.75	m
19	112.7	6.93	d, 5.9	36.4	2.57	dt, 6.5, 6.8, 16.4	112	6.84	d, 5.9	35.5	2.6	brd, 5.4
					2.7	ddd, 7.7, 9.1, 16.5						
20	167.4			187.9			167.8			188.8		
21	17	1.03	d, 6.7	17.3	1.03	d, 6.6	17.3	1.04	d, 6.7	17.4	1.02	d, 6.6
22	21.2	0.97	d, 6.6	21.4	0.94	d, 6.6	21.4	0.97	d, 6.7	21.4	0.94	d, 6.6
23	9.9	1.59	d, 1.1	10.1	1.5	brs	10.1	1.55	d, 1.2	10.1	1.53	brd, 0.9
24	55.6	3.21	s	55.9	3.2	s	55.9	3.22	s	55.8	3.18	s
25	9.1	0.94	d, 7.1	9.9	0.81	d, 7.0	9.9	0.90	d, 7.1	9.6	0.81	brd, 6.2
14-OH		4.18	br									
20-OH		11.07	s									
N-H					7.88	bs					7.86	bs

Table S5. NMR shift comparison of the published janustatin A (1) and janustatins D-F (2-4) in DMSO-d_6.
Compare Figure S41. n/d: not detected.

	janustatin A (**1**)			janustatin D (**2**)			janustatin E (**3**)			janustatin F (**4**)		
No.	δ_C	δ_H	mult. J	δ_C	δ_H	mult. J	δ_C	δ_H	mult. J	δ_C	δ_H	mult. J
1	25.8	1.07	s	25.7	1.06	s	25.3	1.08	s	25.7	1.07	s
2	25.8	1.07	s	25.7	1.06	s	25.3	1.08	s	25.7	1.07	s
3	25.8	1.07	s	25.7	1.06	s	25.3	1.08	s	25.7	1.07	s
4	44.2			44.2			44.8			44.2		
5	218.3			218.2			218.8			218.2		
6	36.8	2.97	m	36.7	2.96	m	37.2	2.96	m	36.7	2.97	m
7	40.6	1.27	ddd, 4.6, 9.7, 13.3	40.8	1.3	m	41.4	1.3	m	40.8	1.32	m
		1.34	ddd, 3.9, 9.9, 13.3									
8	29.4	2.51	ovlp	29.3	2.49	ovlp	29.9	2.5	ovlp	29.3	2.51	ovlp
9	136.8	5.17	d, 9.5	136.7	5.14	d, 9.6	137.1	5.17	d, 9.5	136.8	5.16	d, 9.2
10	131.5			131.5			131.9			131.5		
11	86.9	3.42	ovlp	87.3	3.38	d, 10.1	87.8	3.43	d, 10.1	86.8	3.4	d, 10.3
12	34.1	2.16	m	37.9	1.75	m	38.5	1.86	m	33.7	2.1	m
13	77.8	4.40	brd, 11.0	71.6	4.51	dt, 2.2, 2.4, 13.0	74.9	4.75	d, 12.8	75.0	4.28	dd, 1.40, 11.5
14	63.3	4.52	br	31.2	2.37	m	32	3.06	dd, 13.1, 16.5	63.1	4.38	d, 11.2
					2.79	dd, 12.7, 16.3		2.62	m			
15	160.9			168.1			n/d			170.6		
16	107.2			95.3			n/d			92.4		
17	163.2			161.4			n/d			160.4		
18	142.7	7.68	br	39.6	3.51	m	n/d	7.7	brs	39.2	3.55	m
19	119.5	6.16	d, 6.8	36	2.4	dd, 3.6, 10.3	118.8	6.19	brs	35.5	2.31	m
					2.28	m						
20	175			186.2			n/d			186.4		
21	17	0.96	d, 6.6	16.9	0.95	d, 6.6	17.6	0.96	d, 6.6	16.9	0.95	d, 6.6
22	21.2	0.93	d, 6.6	21.1	0.92	d, 6.6	21.6	0.94	d, 6.6	21.2	0.92	d, 6.6
23	9.9	1.50	brs	9.9	1.48	s	10.4	1.50	s	9.9	1.49	s
24	55.3	3.13	s	55.2	3.12	s	55.7	3.15	s	55.2	3.13	s
25	9.2	0.75	d, 7.0	9.8	0.69	d, 7.0	10.6	0.76	d, 7.0	8.8	0.69	d, 7.0
20-OH		9.01	s									
N-H		6.53	s		9	s					8.83	s

Table S6. Measured integrals of janustatins (1-4) standard curves.

A second order polynomial model was used to fit the data (area [u] $= B2 \cdot c[uM]^2 + B1 \cdot c[uM]$). The intercept was fixed to 0. See Figure S39.

janustatin A (1)		janustatin D (2)		janustatin E (3)		janustatin F (4)	
c[uM]	area[u]	c[uM]	area[u]	c[uM]	area[u]	c[uM]	area[u]
0.0125	2.8E+03	0.0195	1.1E+05	0.01344	2.7E+04	0.0205	3.6E+04
0.0125	2.7E+03	0.0195	9.6E+04	0.01344	2.6E+04	0.0205	4.8E+04
0.125	2.8E+04	0.1949	9.9E+05	0.13439	2.3E+05	0.2048	4.7E+05
0.125	2.7E+04	0.1949	1.1E+06	0.13439	2.6E+05	0.2048	3.9E+05
1.25	3.2E+05	1.9485	9.3E+06	1.34394	2.7E+06	2.0481	6.1E+06
1.25	2.9E+05	1.9485	9.8E+06	1.34394	2.4E+06	2.0481	5.1E+06
12.5	3.0E+06	19.485	6.5E+07	13.4394	2.3E+07	20.481	2.4E+07
12.5	3.0E+06	19.485	7.0E+07	13.4394	2.2E+07	20.481	2.7E+07
41.667	1.3E+07	64.949	1.5E+08	44.7981	5.6E+07	68.269	6.0E+07
41.667	1.1E+07	64.949	1.3E+08	44.7981	7.1E+07	68.269	5.6E+07
83.333	2.6E+07	129.9	2.3E+08	89.5962	1.1E+08	136.54	1.1E+08
83.333	2.4E+07	129.9	2.2E+08	89.5962	1.0E+08	136.54	9.8E+07
125	3.0E+07	194.85	2.8E+08	134.394	1.5E+08	204.81	1.1E+08
125	3.3E+07	194.85	2.7E+08	134.394	1.5E+08	204.81	1.3E+08

		value	standard error
janustatin A (**1**)	B1	3.5E+05	2.2E+04
	B2	-749.8	198.3
janustatin D (**2**)	B1	2.6E+06	1.2E+05
	B2	-6057.5	697.8
janustatin E (**3**)	B1	1.5E+06	7.8E+04
	B2	-2796.3	659.2
janustatin F (**4**)	B1	1.1E+06	6.2E+04
	B2	-2276.2	343.1

Table S7. Relative quantification of janustatins (1-4) in *G. sunshinyii* mutants.

Values are color-coded for each compound (**1**-**4**) from least amount (red) to highest amount (green). 'Ara':0.25% (w/v) arabinose in medium. Standard curves: Figure S39 and Table S6.

	janustatin integrated areas				janustatin concentrations			
G. sunshinyii strain	A (1) area [u]	D (2) area [u]	E (2)3 area [u]	F (4) area [u]	A (1) c [uM]	D (2) c [uM]	E (3) c [uM]	F (4) c [uM]
Δ*janAB* 1	2.0E+2	9.4E+05	1.7E+03	5.0E+02	1.7E-05	1.1E-02	3.4E-05	1.4E-05
Δ*janAB* 2	1.6E+2	9.8E+05	1.5E+03	3.8E+02	1.4E-05	1.1E-02	3.1E-05	1.1E-05
Δ*janAB* 3	0.0E+0	1.2E+06	1.4E+03	2.7E+02	0.0E+00	1.4E-02	2.9E-05	7.6E-06
Δ*janAB* Ara 1	5.2E+2	1.5E+07	3.1E+04	8.0E+02	4.5E-05	1.8E-01	6.4E-04	2.3E-05
Δ*janAB* Ara 2	1.1E+3	1.6E+07	3.1E+04	1.0E+03	9.7E-05	1.8E-01	6.3E-04	2.8E-05
Δ*janAB* Ara 3	5.7E+2	1.0E+07	1.7E+04	2.1E+03	4.9E-05	1.2E-01	3.5E-04	5.9E-05
Δ*janAB* pBAD *janC* 1	3.5E+3	3.1E+04	1.3E+03	9.5E+03	3.0E-04	3.6E-04	2.6E-05	2.7E-04
Δ*janAB* pBAD *janC* 2	1.4E+3	1.9E+04	3.0E+02	2.1E+03	1.2E-04	2.2E-04	6.1E-06	6.0E-05
Δ*janAB* pBAD *janC* 3	7.0E+2	1.4E+04	1.2E+02	1.0E+03	6.0E-05	1.7E-04	2.4E-06	2.8E-05
Δ*janAB* pBAD *janC Ara* 1	1.4E+4	1.1E+08	6.6E+05	5.9E+03	1.2E-03	1.4E+00	1.3E-02	1.7E-04
Δ*janAB* pBAD *janC Ara* 2	1.4E+4	9.7E+07	4.8E+05	8.4E+03	1.2E-03	1.3E+00	9.8E-03	2.4E-04
Δ*janAB* pBAD *janC Ara* 3	2.7E+4	1.2E+08	9.2E+05	9.7E+03	2.3E-03	1.7E+00	1.9E-02	2.7E-04
Δ*janA* 1	2.8E+3	2.0E+04	4.1E+06	7.7E+02	2.4E-04	2.3E-04	8.4E-02	2.2E-05
Δ*janA* 2	1.0E+3	3.4E+04	4.5E+06	7.3E+02	8.7E-05	4.0E-04	9.2E-02	2.1E-05
Δ*janA* 3	6.6E+2	3.9E+04	4.9E+06	3.5E+02	5.7E-05	4.6E-04	1.0E-01	9.9E-06
Δ*janA* Ara 1	5.1E+3	1.8E+06	6.3E+07	2.0E+03	4.4E-04	2.1E-02	1.4E+00	5.5E-05
Δ*janA*Ara 2	7.5E+2	2.7E+03	8.9E+04	5.1E+02	6.4E-05	3.2E-05	1.8E-03	1.4E-05
Δ*janA* Ara 3	5.1E+3	2.7E+06	6.1E+07	1.9E+03	4.4E-04	3.1E-02	1.4E+00	5.4E-05
Δ*janA* pBAD *janB* 1	1.3E+3	1.4E+05	7.9E+03	3.0E+03	1.1E-04	1.6E-03	1.6E-04	8.4E-05
Δ*janA* pBAD *janB* 2	3.7E+2	3.8E+04	9.0E+03	5.3E+02	3.2E-05	4.4E-04	1.8E-04	1.5E-05
Δ*janA* pBAD *janB* 3	2.8E+2	3.1E+04	9.0E+03	2.5E+02	2.4E-05	3.7E-04	1.8E-04	7.1E-06
Δ*janA* pBAD *janB Ara* 1	3.2E+3	1.9E+07	3.3E+07	1.4E+03	2.8E-04	2.2E-01	7.1E-01	4.0E-05
Δ*janA* pBAD *janB Ara* 2	4.6E+3	1.9E+07	4.7E+07	2.0E+03	4.0E-04	2.3E-01	1.0E+00	5.8E-05
Δ*janA* pBAD *janB Ara* 3	6.0E+3	2.3E+07	5.4E+07	2.6E+03	5.2E-04	2.8E-01	1.2E+00	7.4E-05
Δ*janB* 1	4.1E+4	1.7E+05	1.6E+04	7.3E+05	3.5E-03	2.0E-03	3.2E-04	2.6E-02
Δ*janB* 2	1.1E+5	1.8E+05	4.7E+03	1.1E+06	9.6E-03	2.1E-03	9.7E-05	3.1E-02
Δ*janB* 3	1.1E+5	1.3E+05	3.0E+03	9.4E+05	9.2E-03	1.5E-03	6.2E-05	2.7E-02
Δ*janB* Ara 1	4.3E+5	2.4E+05	6.7E+03	4.5E+06	3.7E-02	2.8E-03	1.4E-04	1.3E-01
Δ*janB* Ara 2	4.4E+5	2.4E+05	5.7E+03	3.6E+06	3.8E-02	2.8E-03	1.2E-04	1.0E-01
Δ*janB* Ara 3	5.6E+5	2.4E+05	7.5E+03	5.2E+06	4.8E-02	2.8E-03	1.5E-04	1.5E-01

Δ*janB* pBAD *janC* 1	1.0 E+3	1.2 E+03	1.1 E+03	5.0 E+03	8.6 E-05	1.4 E-05	2.2 E-05	1.4 E-04
Δ*janB* pBAD *janC* 2	8.5 E+2	2.5 E+02	9.5 E+01	6.2 E+03	7.3 E-05	3.0 E-06	1.9 E-06	1.8 E-04
Δ*janB* pBAD *janC* 3	1.5 E+3	1.6 E+05	8.6 E+03	3.6 E+04	1.3 E-04	1.9 E-03	1.8 E-04	1.0 E-03
Δ*janB* pBAD *janC Ara* 1	2.7 E+7	8.3 E+06	3.3 E+05	4.3 E+07	3.0 E+00	9.8 E-02	6.8 E-03	1.4 E+00
Δ*janB* pBAD *janC Ara* 2	2.4 E+7	8.3 E+06	2.4 E+05	5.0 E+07	2.5 E+00	9.8 E-02	5.0 E-03	1.6 E+00
Δ*janB* pBAD *janC Ara* 3	2.4 E+7	1.0 E+07	3.2 E+05	4.9 E+07	2.5 E+00	1.2 E-01	6.6 E-03	1.6 E+00
wild type 1	1.9 E+6	3.2 E+05	6.0 E+05	3.4 E+05	1.6 E-01	3.8 E-03	1.2 E-02	9.7 E-03
wild type 2	2.3 E+6	4.2 E+05	6.6 E+05	3.5 E+05	2.0 E-01	4.9 E-03	1.4 E-02	9.8 E-03
wild type 3	2.6 E+6	5.1 E+05	9.2 E+05	3.9 E+05	2.3 E-01	6.0 E-03	1.9 E-02	1.1 E-02
wild type Ara 1	1.5 E+7	3.0 E+06	9.1 E+04	7.3 E+04	1.4 E+00	3.5 E-02	1.9 E-03	2.1 E-03
wild type Ara 2	1.5 E+7	3.2 E+06	8.4 E+04	1.1 E+05	1.4 E+00	3.7 E-02	1.7 E-03	3.0 E-03
wild type Ara 3	2.0 E+7	3.6 E+06	1.2 E+05	9.4 E+04	2.0 E+00	4.2 E-02	2.4 E-03	2.7 E-03

Table S8. Relative quantification of janustatins (1-4) in *G. sunshinyii* complementation strains.
Values are color-coded for each compound (**1-4**) from least amount (red) to highest amount (green). 'Ara':0.25% arabinose in medium. Standard curves: Figure S39 and Table S6.

	janustatin integrated areas				janustatin concentrations			
***G. sunshinyii* strain**	**A (1) area [u]**	**D (2) area [u]**	**E (3) area [u]**	**F (4) area [u]**	**A (1) c [uM]**	**D (2) c [uM]**	**E (3) c [uM]**	**F (4) c [uM]**
Δ*janA* pBMTBX-2 empty 1	9.3E+01	9.9E+02	3.5E+04	1.1E+02	1.6E-05	2.3E-05	1.5E-03	6.4E-06
Δ*janA* pBMTBX-2 empty 2	1.3E+02	2.5E+04	2.1E+06	2.4E+02	2.3E-05	5.8E-04	8.8E-02	1.4E-05
Δ*janA* pBMTBX-2 empty 3	7.6E+01	2.0E+05	3.8E+06	2.4E+02	1.3E-05	4.7E-03	1.6E-01	1.3E-05
Δ*janA* pBMTBX-2 empty Ara 1	4.7E+01	1.0E+04	9.0E+05	1.8E+02	8.1E-06	2.4E-04	3.7E-02	9.9E-06
Δ*janA* pBMTBX-2 empty Ara 2	9.0E+00	3.5E+04	2.1E+05	1.3E+02	1.5E-06	8.2E-04	8.5E-03	7.3E-06
Δ*janA* pBMTBX-2 empty Ara 3	9.2E+01	8.5E+04	8.2E+05	9.4E+01	1.6E-05	2.0E-03	3.4E-02	5.3E-06
Δ*janA* pBMTBX-2 *janA* 1	1.5E+04	1.8E+03	2.0E+04	2.6E+02	2.5E-03	4.3E-05	8.3E-04	1.5E-05
Δ*janA* pBMTBX-2 *janA* 2	2.8E+05	4.3E+04	4.5E+05	5.1E+03	4.8E-02	1.0E-03	1.9E-02	2.9E-04
Δ*janA* pBMTBX-2 *janA* 3	1.5E+06	7.8E+04	9.7E+05	2.1E+04	2.7E-01	1.8E-03	4.0E-02	1.2E-03
Δ*janA* pBMTBX-2 *janA* Ara 1	5.0E+06	1.8E+05	9.0E+04	2.2E+05	8.8E-01	4.1E-03	3.7E-03	1.2E-02
Δ*janA* pBMTBX-2 *janA* Ara 2	3.8E+05	3.3E+04	5.4E+04	3.5E+04	6.5E-02	7.8E-04	2.2E-03	2.0E-03
Δ*janA* pBMTBX-2 *janA* Ara 3	1.4E+05	3.0E+04	7.6E+04	2.3E+04	2.3E-02	7.1E-04	3.1E-03	1.3E-03
Δ*janB* pBMTBX-2 empty 1	5.0E+03	2.9E+03	1.1E+02	3.7E+04	8.7E-04	6.8E-05	4.5E-06	2.1E-03
Δ*janB* pBMTBX-2 empty 2	1.7E+04	4.1E+03	4.6E+01	6.5E+04	3.0E-03	9.5E-05	1.9E-06	3.7E-03
Δ*janB* pBMTBX-2 empty 3	5.2E+03	3.8E+03	1.1E+02	4.2E+04	9.0E-04	8.8E-05	4.4E-06	2.4E-03
Δ*janB* pBMTBX-2 empty Ara 1	1.6E+04	1.6E+03	2.1E+02	1.3E+05	2.8E-03	3.8E-05	8.8E-06	7.6E-03
Δ*janB* pBMTBX-2 empty Ara 2	1.4E+04	4.7E+03	9.4E+01	7.5E+04	2.4E-03	1.1E-04	3.9E-06	4.2E-03
Δ*janB* pBMTBX-2 empty Ara 3	3.3E+03	4.6E+03	1.1E+02	3.2E+04	5.6E-04	1.1E-04	4.6E-06	1.8E-03
Δ*janB* pBMTBX-2 *janB* 1	1.6E+04	2.2E+03	1.5E+02	2.0E+04	2.8E-03	5.1E-05	6.0E-06	1.1E-03
Δ*janB* pBMTBX-2 *janB* 2	5.6E+03	9.5E+02	9.4E+01	1.2E+04	9.7E-04	2.2E-05	3.9E-06	6.9E-04
Δ*janB* pBMTBX-2 *janB* 3	1.4E+05	4.5E+03	1.7E+02	8.1E+04	2.4E-02	1.0E-04	7.1E-06	4.6E-03
Δ*janB* pBMTBX-2 *janB* Ara 1	3.9E+04	1.1E+04	1.5E+03	1.7E+04	6.6E-03	2.7E-04	6.0E-05	9.6E-04
Δ*janB* pBMTBX-2 *janB* Ara 2	1.7E+05	4.6E+04	2.1E+03	8.1E+04	2.9E-02	1.1E-03	8.4E-05	4.6E-03
Δ*janB* pBMTBX-2 *janB* Ara 3	2.2E+05	2.3E+04	1.5E+03	1.3E+05	3.8E-02	5.4E-04	6.0E-05	7.1E-03
wild type pBMTBX-2 empty 1	1.7E+05	1.1E+04	1.1E+04	2.6E+03	2.9E-02	2.6E-04	4.6E-04	1.5E-04
wild type pBMTBX-2 empty 2	1.4E+06	2.7E+04	4.5E+03	2.6E+04	2.4E-01	6.3E-04	1.8E-04	1.5E-03
wild type pBMTBX-2 empty 3	4.2E+05	2.1E+04	4.4E+03	1.6E+04	7.3E-02	4.8E-04	1.8E-04	8.8E-04
wild type pBMTBX-2 empty Ara 1	6.4E+05	7.7E+04	2.4E+03	1.2E+03	1.1E-01	1.8E-03	1.0E-04	6.7E-05
wild type pBMTBX-2 empty Ara 2	1.7E+06	8.6E+04	1.1E+04	9.4E+04	2.9E-01	2.0E-03	4.6E-04	5.3E-03
wild type pBMTBX-2 empty Ara 3	2.9E+06	5.8E+04	3.6E+03	1.8E+05	5.0E-01	1.4E-03	1.5E-04	1.0E-02

Table S9. Determination of janustatin A (1) titers in different *G. sunshinyii* strains.

The janustatin A (**1**) base peak in extracted ion chromatograms (*m/z* 448.2694) of organic extracts prepared from various strains was integrated. The mean of three biological replicates was used to estimate the relative janustatin A (**1**) titers (Figure 2 and Figure S6). Samples in **a** and **b** were compared to the respective wild type measurements. Since the variance of pBAD *janB* pBMTBX2-*janA* and refactored were not comparable to the respective wild type variances, a Welch's t-test was used to compare the sample groups relative to the wild type. Standard curves: Figure S39 and Table S6.

***G. sunshinyii* strains**	**integral of EIC**	**average integral**	**standard deviation**	**Integral relative to wild type**	**p-value**
**A** in Figure S43					
wild type 1	5.87E+08				
wild type 2	7.22E+08	6.46E+08	6.92E+07	1.00	
wild type 3	6.30E+08				
Δ*janA* pBAD *janB* pBMTBX2-*janA* 1	2.05E+08				
Δ*janA* pBAD *janB* pBMTBX2-*janA 2*	1.54E+08	2.18E+08	7.11E+07	0.34	0.002
Δ*janA* pBAD *janB* pBMTBX2-*janA 3*	2.95E+08				
pBAD *janB* pBMTBX2-*janA* 1	4.71E+08				
pBAD *janB* pBMTBX2-*janA 2*	7.87E+07	3.01E+08	2.01E+08	0.47	0.084
pBAD *janB* pBMTBX2-*janA 3*	3.52E+08				
**B** in Figure S43					
wild type 1	6.88E+08				
wild type 2	5.57E+08	5.83E+08	9.53E+07	1.00	
wild type 3	5.03E+08				
pBAD *janB* 1	6.17E+08				
pBAD *janB* 2	6.81E+08	6.72E+08	5.08E+07	1.15	0.247
pBAD *janB* 3	7.17E+08				
refactored 1	2.43E+08				
refactored 2	2.52E+08	2.62E+08	2.45E+07	0.45	0.022
refactored 3	2.89E+08				

Pyhton script to integrate janustatin peaks in LC-MS measurements for the mutants as well as the chemical standards.

```
import numpy as np
import pandas as pd
import os

def integrate_EIC(file_path, concentration):
    EIC = pd.read_table(file_path) #import dataframe
    #define peak retention times
    janustatinA_peak = EIC[ (EIC["Time"] > 7.02) & (EIC["Time"] < 7.15)]
    janustatinD_peak = EIC[ (EIC["Time"] > 7.20) & (EIC["Time"] < 7.40)]
    janustatinE_peak = EIC[ (EIC["Time"] > 7.70) & (EIC["Time"] < 7.90)]
    janustatinF_peak = EIC[ (EIC["Time"] > 6.87) & (EIC["Time"] < 6.96)]

    #calculate areas of peaks in current EIC
    janustatinA_area = np.trapz(y = janustatinA_peak["Intensity"], x = janustatinA_peak["Time"])
    janustatinD_area = np.trapz(y = janustatinD_peak["Intensity"], x = janustatinD_peak["Time"])
    janustatinE_area = np.trapz(y = janustatinE_peak["Intensity"], x = janustatinE_peak["Time"])
    janustatinF_area = np.trapz(y = janustatinF_peak["Intensity"], x = janustatinF_peak["Time"])

    #write data into dictionary
    data = {"c[uM]": concentration,
            "area janustatin A": [janustatinA_area],
            "area janustatin D": [janustatinD_area],
            "area janustatin E": [janustatinE_area],
            "area janustatin F": [janustatinF_area]}
    return data

def read_concentration(file_name): #read concentration from file name; this is necessary because filenames cant have a . (sometimes used p instead)
    concentration = file_name[12:-7]
    if concentration[0] == "0":
        concentration = "0." + concentration[1:]
    concentration = float(concentration.replace("p","."))
    return concentration

def integrate_standard(path): #integrate over files in directory
    df = pd.DataFrame(columns= ["c[uM]", "area janustatin A", "area janustatin D", "area janustatin E", "area janustatin F"])
    for file in os.listdir(path):
        current_path = (path + "/" + file)
        current_concentration = read_concentration(file)
        current_integral = integrate_EIC(current_path, current_concentration)
        current_df = pd.DataFrame.from_dict(current_integral)
        df = pd.concat([df, current_df])
    df = df.sort_values("c[uM]")
    return df

def integrate_EIC_sample(file_path, file):
    EIC = pd.read_table(file_path) #import dataframe
    #define peak retention times
    janustatinA_peak = EIC[ (EIC["Time"] > 7.02) & (EIC["Time"] < 7.15)]
    janustatinD_peak = EIC[ (EIC["Time"] > 7.20) & (EIC["Time"] < 7.40)]
    janustatinE_peak = EIC[ (EIC["Time"] > 7.70) & (EIC["Time"] < 7.90)]
    janustatinF_peak = EIC[ (EIC["Time"] > 6.87) & (EIC["Time"] < 6.96)]

    #calculate areas of peaks in current EIC
    janustatinA_area = np.trapz(y = janustatinA_peak["Intensity"], x = janustatinA_peak["Time"])
    janustatinD_area = np.trapz(y = janustatinD_peak["Intensity"], x = janustatinD_peak["Time"])
    janustatinE_area = np.trapz(y = janustatinE_peak["Intensity"], x = janustatinE_peak["Time"])
    janustatinF_area = np.trapz(y = janustatinF_peak["Intensity"], x = janustatinF_peak["Time"])

    current_replicate = file[-5]
    arabinose = file.__contains__("Ara")
    plasmid = "empty"
    if file[6] == "A":
        plasmid = "janA"
    elif file [6] == "B":
        plasmid = "janB"

    #write data into dictionary
    data = {"plasmid": plasmid,
            "replicate": current_replicate,
            "arabinose": arabinose,
```

```
            "area janustatin A": [janustatinA_area],
            "area janustatin D": [janustatinD_area],
            "area janustatin E": [janustatinE_area],
            "area janustatin F": [janustatinF_area]}
    return data

def integrate_sample(path): #integrate over files in directory
    df = pd.DataFrame(columns= ["plasmid","replicate","arabinose", "area janustatin A", "area janustatin D", "area janustatin E", "area janustatin F"])
    for file in os.listdir(path):
        current_path = (path + "/" + file)
        current_integral = integrate_EIC_sample(current_path, file)
        current_df = pd.DataFrame.from_dict(current_integral)
        current_df.index = [file[:-4]]
        df = pd.concat([df, current_df])
    return df

#calculate standard curves
path_janustatinA = r"C:/Users/stefanl/polybox2/ETH/janustatin_biosynthesis/EICs/standards/janustatinA"
janustatinA = integrate_standard(path_janustatinA)
path_janustatinD = r"C:/Users/stefanl/polybox2/ETH/janustatin_biosynthesis/EICs/standards/janustatinD"
janustatinD = integrate_standard(path_janustatinD)
path_janustatinE = r"C:/Users/stefanl/polybox2/ETH/janustatin_biosynthesis/EICs/standards/janustatinE"
janustatinE = integrate_standard(path_janustatinE)
path_janustatinF = r"C:/Users/stefanl/polybox2/ETH/janustatin_biosynthesis/EICs/standards/janustatinF"
janustatinF = integrate_standard(path_janustatinF)
#generate data for strains with plasmid
path_plasmids = r"C:/Users/stefanl/polybox2/ETH/janustatin_biosynthesis/EICs/pBMTBX2"
area_plasmids = integrate_sample(path_plasmids)
#generate data for mutants
path_mutants = r"C:/Users/stefanl/polybox2/ETH/janustatin_biosynthesis/EICs/mutants"
area_mutants = integrate_sample(path_mutants)
```

References

(1.) Chung, E. J., Park, J. A., Jeon, C. O., and Chung, Y. R. *Gynuella Sunshinyii* Gen. Nov., Sp. Nov., an Antifungal Rhizobacterium Isolated from a Halophyte, *Carex Scabrifolia* Steud. *Int. J. Syst. Evol. Microbiol.* **2015,** *65* (Pt_3), 1038-1043.

(2.) Le Roux, F., Binesse, J., Saulnier, D., and Mazel, D. Construction of a *Vibrio Splendidus* Mutant Lacking the Metalloprotease Gene *Vsm* by Use of a Novel Counterselectable Suicide Vector. *Appl. Environ. Microbiol.* **2007,** *73* (3), 777-784.

(3.) Gibson, D. G., Young, L., Chuang, R. Y., Venter, J. C., Hutchison, C. A., 3rd, and Smith, H. O. Enzymatic Assembly of DNA Molecules up to Several Hundred Kilobases. *Nat. Methods* **2009,** *6* (5), 343-345.

(4.) Shi, Y. M., Hirschmann, M., Shi, Y. N., Ahmed, S., Abebew, D., Tobias, N. J., Grün, P., Crames, J. J., Pöschel, L., Kuttenlochner, W., Richter, C., Herrmann, J., Müller, R., Thanwisai, A., Pidot, S. J., Stinear, T. P., Groll, M., Kim, Y., and Bode, H. B. Global Analysis of Biosynthetic Gene Clusters Reveals Conserved and Unique Natural Products in Entomopathogenic Nematode-Symbiotic Bacteria. *Nat. Chem.* **2022,** *14* (6), 701-712.

(5.) Thoma, S., and Schobert, M. An Improved *Escherichia Coli* Donor Strain for Diparental Mating. *FEMS Microbiol. Lett.* **2009,** *294* (2), 127-132.

(6.) Acebal, C., Alcazar, R., Cañedo, L. M., De La Calle, F., Rodriguez, P., Romero, F., and Fernandez Puentes, J. L. Two Marine Agrobacterium Producers of Sesbanimide Antibiotics. *J. Antibiot.* **1998,** *51*, 64-67.

(7.) Duitman, E. H., Hamoen, L. W., Rembold, M., Venema, G., Seitz, H., Saenger, W., Bernhard, F., Reinhardt, R., Schmidt, M., Ullrich, C., Stein, T., Leenders, F., and Vater, J. The Mycosubtilin Synthetase of *Bacillus Subtilis* Atcc6633: A Multifunctional Hybrid between a Peptide Synthetase, an Amino Transferase, and a Fatty Acid Synthase. *Proc. Natl. Acad. Sci. U.S.A.* **1999,** *96* (23), 13294-13299.

(8.) Edgar, R. C. Muscle: Multiple Sequence Alignment with High Accuracy and High Throughput. *Nucleic Acids Res.* **2004,** *32*, 1792-1797.

(9.) Ueoka, R., Sondermann, P., Leopold-Messer, S., Liu, Y., Suo, R., Bhushan, A., Vadakumchery, L., Greczmiel, U., Yashiroda, Y., Kimura, H., Nishimura, S., Hoshikawa, Y., Yoshida, M., Oxenius, A., Matsunaga, S., Williamson, R. T., Carreira, E. M., and Piel, J. Genome-Based Discovery and Total Synthesis of Janustatins, Potent Cytotoxins from a Plant-Associated Bacterium. *Nat. Chem.* **2022,** *14*, 1193-1201.
